# Mechanistic insights into the competition between electrochemical CO_2_ reduction and hydrogen evolution on Ag-based electrocatalysts *via operando* Raman spectroscopy

**DOI:** 10.1039/d5sc04774a

**Published:** 2025-10-30

**Authors:** Kinran Lau, Muhammad Adib Abdillah Mahbub, Nini Zhang, Anirudha Shekhawat, Xin Wang, Sabine Seisel, Ridha Zerdoumi, Wolfgang Schuhmann

**Affiliations:** a Analytical Chemistry—Center for Electrochemical Sciences (CES), Faculty of Chemistry and Biochemistry, Ruhr University Bochum Universitsätsstr. 150 Bochum 44780 Germany wolfgang.schuhmann@rub.de

## Abstract

To establish electrochemical CO_2_ reduction (CO_2_RR) as a viable industrial route for fuel and chemical production, it is crucial to sustain CO_2_RR over the competing hydrogen evolution reaction (HER) even at high current densities. However, the underlying mechanism of HER dominance at higher overpotentials remains poorly understood. Here, using *operando* Raman spectroscopy, we first probe the CO_2_-to-CO pathway on Ag catalysts modified with alkaline earth metals (AgMg, AgCa, AgSr, AgBa) in a Na^+^-containing electrolyte. These modified catalysts exhibit more pronounced Raman features than pure Ag, enabling the detection of key CO_2_RR intermediates. Notably, AgBa shows the clearest progression of intermediates with increasing cathodic potential: CO_2_ → *COO^−^ → *COOH → *CO, providing direct spectroscopic evidence for the proposed CO formation mechanism. At potentials more negative than −0.3 V *vs.* RHE, CO_2_RR-related signals diminish, but this is accompanied by the emergence of a broad band at ∼532 cm^−1^, which is assigned to the libration of interfacial water. This feature strongly correlates with the visible occurrence of the HER current, suggesting its role in HER initiation. We propose that an increasingly negatively charged electrode drives the reorientation of interfacial water molecules into an “H-down” configuration, creating a favorable geometry to trigger HER. The accumulation of this ordered interfacial water structure may represent the molecular origin of HER dominance at high overpotentials. We hope that these insights provide a framework for designing strategies to suppress HER and promote CO_2_RR by controlling interfacial water reorientation.

## Abstract 2

Operando Raman spectroscopy captures the gradual transition from CO_2_ reduction to hydrogen evolution under increasingly cathodic potentials, revealing the pivotal role of interfacial water reorientation in initiating HER.

## Graphical abstract

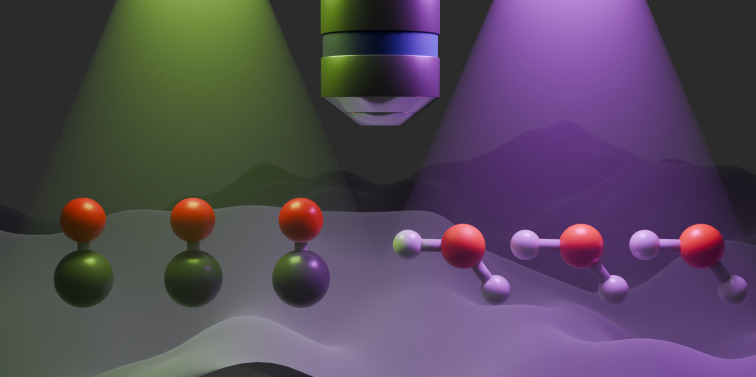

## Introduction

The urgent need to tackle rising atmospheric CO_2_ levels has driven extensive research into the electrochemical CO_2_ reduction reaction (CO_2_RR) as a sustainable route for fuel and chemical production.^1^ For practical applications, CO_2_RR has to be operated at industrially relevant current densities (hundreds of mA cm^−2^) with high selectivity and long-term stability for the generation of valuable products such as CO, ethylene, or ethanol.^2,3^ However, the competing hydrogen evolution reaction (HER) often undermines CO_2_RR efficiency.^4,5^ To enhance CO_2_ mass transport to the catalyst interface and suppress HER, gas-fed flow cells using gas diffusion electrodes (GDEs) have largely replaced conventional liquid-fed H-cells.^6,7^ Despite this advancement, HER often outcompetes CO_2_RR at higher overpotentials and eventually becomes the dominant reaction, which is usually accompanied by flooding of the GDE.^8,9^ When flooding happens, electrolyte penetrates the hydrophobic gas diffusion layer (GDL), reducing CO_2_ availability at the catalyst interface further promoting HER over CO_2_RR.^10^ Hence, to design better CO_2_RR catalysts, it is crucial to understand the exact mechanism driving this gradual transition from the desirable CO_2_RR to the competitive HER.

However, probing this dynamic process requires techniques capable of characterizing the electrode interface under operating conditions, which is often not trivial. To this end, *operando* Raman spectroscopy has demonstrated itself as a powerful, non-invasive tool to directly observe key catalytic species during electrochemical reactions such as CO_2_RR and HER.^11–13^ A distinct advantage of Raman spectroscopy is its broad spectral range, spanning from low-frequency M–O and M–C vibrations (<1000 cm^−1^, where M stands for metal) to mid-range COO^−^ and CO stretches (∼1350–2150 cm^−1^), and high-frequency OH stretches from water molecules (∼3400–3600 cm^−1^).^14^ This wide coverage enables a more comprehensive view of the catalytic interface compared to surface-enhanced infrared absorption spectroscopy (SEIRAS), which typically cannot access vibrations below ∼1000 cm^−1^.^15^ Due to the relatively small scattering cross-section of water,^16^ Raman spectroscopy is also well-suited for examining aqueous systems without the bulk water signals overwhelming the spectrum. Moreover, common CO_2_RR catalysts such as Ag, Au, and Cu exhibit surface-enhanced Raman scattering (SERS) on rough surfaces, greatly amplifying the Raman signals near the interface up to a factor of 10^5^–10^6^,^17^ which is beneficial for detecting reaction intermediates. For Ag nanoparticles, it has been estimated that the SERS effect can be probed up to 5 nm away from the surface.^18^ Overall, these advantages make *operando* Raman spectroscopy a valuable method for elucidating the competition between CO_2_RR and HER under reaction conditions.

In this study, contrary to the popular choice of Cu capable of yielding C_2+_ products,^19^ we focus on Ag as a simple model system for *operando* Raman analysis. Unlike Cu, which produces a complex mixture of products and may contain Cu^+^ species that complicate mechanistic interpretation,^20–23^ Ag follows a well-defined CO_2_RR pathway (CO_2_ → *COO^−^ → *COOH → *CO, where * denotes an adsorbed species), with CO being the dominant product:^24–27^
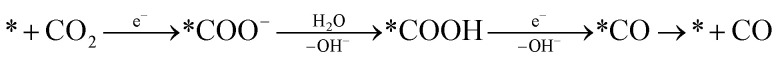


This provides a straightforward readout, where the gradual transition from CO_2_RR to HER at more cathodic potentials is marked by a decrease in CO production and a corresponding increase in H_2_. However, a key challenge of using Ag is its weak CO binding energy, which is even lower than that of Au and Cu,^26,28,29^ making it potentially difficult to detect CO_2_RR intermediates.

To address this problem, we draw inspiration from Cu-based catalysts, where the incorporation of alkaline earth metals has been demonstrated as an effective strategy to increase the Raman detectability of CO and improve CO_2_RR performance.^30–33^ For instance, Xie *et al.* screened 109 Cu-based bimetallic combinations and identified Cu–Mg as the most active catalyst with up to 80% C_2+_ faradaic efficiency (FE) at −1 A cm^−2^.^30^ On a similar note, Xu *et al.* reported a Cu/BaO catalyst achieving 61% FE for C_2+_ alcohols at −400 mA cm^−2^, which was attributed to the metal/oxide interface stabilizing the hydroxyl-containing CO_2_RR intermediates.^32^ Motivated by these findings, we investigate whether depositing small amounts of Group 2 metals (Mg, Ca, Sr, Ba) onto Ag can similarly strengthen CO binding and thereby enhance CO_2_RR intermediate detection by Raman spectroscopy. Notably, while the Group 2-modified catalysts do not surpass pure Ag in overall CO_2_RR activity, they significantly improve the Raman visibility of surface intermediates. On pristine Ag, CO_2_RR species are barely detectable, whereas AgBa shows distinct peaks corresponding to *COOH and *CO, with additional shoulder features attributable to *COO^−^. Interestingly, these CO_2_RR signals become negligible beyond −0.3 V *vs.* RHE, but this disappearance coincides with the emergence of a broad peak at ∼532 cm^−1^, which is assigned to the librational mode of interfacial water. This spectroscopic signature aligns with a sharp rise in current associated with HER. Taken together, we propose that as the electrode becomes increasingly negatively charged, more interfacial water molecules reorient their hydrogen atoms towards the surface into an “H-down” configuration, creating a favorable geometry for HER initiation. This interfacial water reorganization may represent the molecular origin behind the gradual transition from CO_2_RR to the competitive HER. Overall, our work highlights the capability of *operando* Raman spectroscopy to directly probe surface intermediates and offer mechanistic insights into the interplay between CO_2_RR and HER.

## Results and discussion

### Synthesis and characterization of modified Ag catalysts

**Fig. 1:**
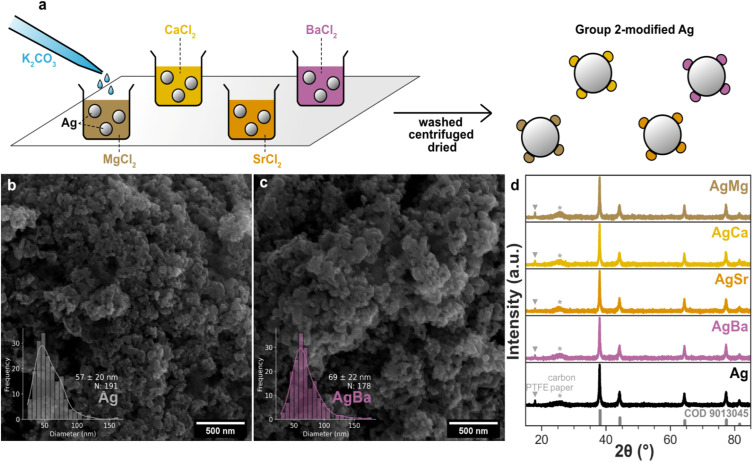
(a) Schematic of the synthesis of Group 2-modified Ag catalysts *via* carbonate precipitation. SEM images and particle size distributions of (b) Ag and (c) AgBa catalysts. (d) XRD patterns of Group 2-modified Ag catalysts drop-cast on carbon paper.

Group 2-modified Ag catalysts (AgMg, AgCa, AgSr, AgBa) were prepared *via* a simple precipitation method. As depicted schematically in Fig. 1a, commercial Ag nanoparticles (Sigma-Aldrich, 99.5%, <100 nm) were first dispersed in an aqueous solution of metal chlorides (MgCl_2_, CaCl_2_, SrCl_2_, BaCl_2_) with a metal-to-Ag molar ratio of 2%. An excess of K_2_CO_3_ was then added dropwise to precipitate the respective metal carbonates onto the Ag surface. The resulting materials were washed, centrifuged, and dried before collection (see Experimental section in SI for details). Unlike common coprecipitation methods, where Ag and dopant precursors are precipitated together, we start with the same batch of commercial Ag particles, which minimizes the variations in particle size across samples. Scanning electron microscopy (SEM) analysis shows that unmodified Ag particles have an average diameter of 57 ± 20 nm (Fig. 1b). After Group 2 addition, the primary particle size only increases slightly, reaching 69 ± 22 nm for AgBa (Fig. 1c), while other modified Ag samples also fall within a similar range of 61–69 nm (Table S1 and Fig. S1–S3). We chose carbonate rather than hydroxide precipitation for two main reasons. First, metal hydroxides readily convert to carbonates upon exposure to CO_2_ during electrochemical testing. But more importantly, the solubility product (*K*_sp_) of Group 2 hydroxides increases dramatically down the series, spanning eight orders of magnitude from Mg(OH)_2_ (5.61 × 10^−12^ mol^3^ L^−3^) to Ba(OH)_2_ (2.55 × 10^−4^ mol^3^ L^−3^) (Table S2). This means that significantly higher OH^−^ concentrations would be required to precipitate Ba(OH)_2_ compared to Mg(OH)_2_. In contrast, Group 2 carbonates have consistently low solubility, with *K*_sp_ values of 6.82 × 10^−6^ mol^2^ L^−2^ for MgCO_3_ and roughly 10^−9^ to 10^−10^ mol^2^ L^−2^ for CaCO_3_, SrCO_3_, and BaCO_3_ (Table S2), which allows for more comparable precipitation conditions across all samples. Despite this modification, X-ray diffraction (XRD) patterns (Fig. 1d) show no distinct reflections for the metal carbonates, likely due to their low loading. Only reflections corresponding to Ag are observed, along with signals from PTFE (2*θ* = 18°) and carbon paper (2*θ* = 26°).^34^ However, inductively coupled plasma mass spectrometry (ICP-MS) and X-ray photoelectron spectroscopy (XPS) confirm the successful deposition of Group 2 elements onto the Ag particles. ICP-MS analysis (Table S3) consistently shows 0.01–0.04 mol% Group 2 content relative to Ag for all samples, except for AgSr, which exhibits a notably higher value (0.9 mol% Sr). This high level of Sr likely reflects the lowest solubility constant of SrCO_3_ (5.6 × 10^−10^ mol^2^ L^−2^, Table S2) among Group 2 carbonates, though it remains lower than the nominal 2% initially added. XPS measurements (Fig. S4–S6) further reveal the distribution between bulk and surface. While Mg and Ca were not detected likely due to their trace amount, both Sr and Ba were clearly observed. As a representative example, AgBa records a bulk Ba content of 0.04 mol% by ICP-MS, but a significantly higher surface-sensitive Ba/Ag ratio of 0.55% by XPS, suggesting the presence of Ba at the surface following carbonate precipitation.

### Electrochemical CO_2_ reduction

**Fig. 2:**
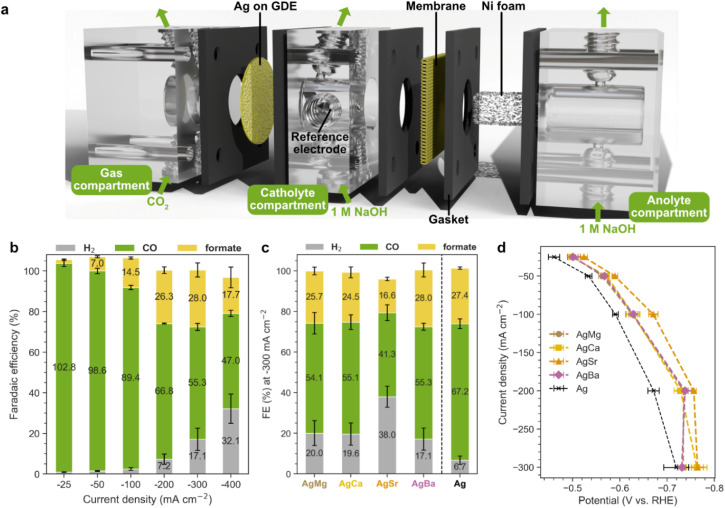
(a) Schematic of the flow cell used for electrochemical CO_2_ reduction on catalyst-coated GDEs in 1 M NaOH (pH 14). (b) Faradaic efficiencies of CO, formate, and H_2_ for the AgBa catalyst at current densities in the range from −25 to −400 mA cm^−2^. (c) Faradaic efficiencies at −300 mA cm^−2^ for all Group 2-modified catalysts (AgMg, AgCa, AgSr, AgBa) compared to pure Ag. (d) Current densities and iR-corrected potentials for all samples. Data beyond −300 mA cm^−2^ are not shown here due to vigorous bubble formation interfering with accurate resistance measurements. Error bars represent ±1 standard deviation from triplicate measurements.

Electrochemical CO_2_RR measurements were performed using a custom flow cell setup illustrated in Fig. 2a. As mentioned in the introduction, a GDE was employed to enhance CO_2_ mass transport and availability, where CO_2_ was supplied from the back side of the GDE and reached the catalyst layer at the electrolyte interface. The working electrode consisted of the Group 2-modified Ag catalysts drop-cast onto the GDE, while Ni foam served as the counter electrode. A 1 M NaOH solution was used as electrolyte, with the catholyte and anolyte compartments separated by an anion exchange membrane. Gaseous products were quantified using online gas chromatography (GC), while liquid products were sampled at each current density and analysed by high-performance liquid chromatography (HPLC). Chronopotentiometric measurements were carried out in a current density range from −25 mA cm^−2^ to −400 mA cm^−2^ for all catalysts (Ag, AgMg, AgCa, AgSr, AgBa), and only three main products were detected: CO, formate, and H_2_ (Fig. S7–S16).

Using AgBa as a representative example, Fig. 2b presents the evolution of product distribution as the current density increases. At low current densities (up to −100 mA cm^−2^), CO is the predominant product (>90% FE), with only minor amounts of formate and H_2_ formed. However, at higher current densities, FE_CO_ declines while FE_formate_ and FE_H2_ increase, suggesting that CO_2_RR becomes limited by mass transport, while the competing HER starts to take over. At −400 mA cm^−2^, FE_H2_ reaches up to 32% for AgBa. The CO_2_RR performance of all catalysts is summarized in Fig. 2c comparing their product selectivity at −300 mA cm^−2^, which is the current density when HER becomes evident. A higher FE_CO_ and a lower FE_H2_ indicate more favorable CO_2_RR performance, and *vice versa*. Among the modified catalysts, AgMg, AgCa, and AgBa with comparable Group 2 loading (Table S3) exhibit similar CO_2_RR activity at −300 mA cm^−2^, with a FE_CO_ at around 55%, a FE_formate_ of close to 25%, and a FE_H2_ below 20%. In contrast, AgSr exhibits worse selectivity, achieving only 41% FE_CO_ and 38% FE_H2_, likely due to the excessively high Sr content compared to other samples (Table S3), which appears to be detrimental to CO_2_RR activity. Notably, all Group 2-modified catalysts demonstrate decreased CO_2_RR activity compared to pure Ag, which delivers a FE_CO_ of 67% and only a FE_H2_ of 7% at the same current density.

This inferior performance of the modified catalysts is likely due to the non-conductive nature of the deposited metal carbonates, which contribute to ohmic losses and partially block the active surface sites of Ag. The current density *vs.* potential (iR-corrected *vs.* RHE) curves in Fig. 2d reveal a similar trend, where pure Ag has the lowest overpotential, followed by AgMg, AgCa, and AgBa with nearly identical potential profiles, while the worst performing AgSr displays the highest overpotential.

### Qualitative analysis of Raman spectra

**Fig. 3:**
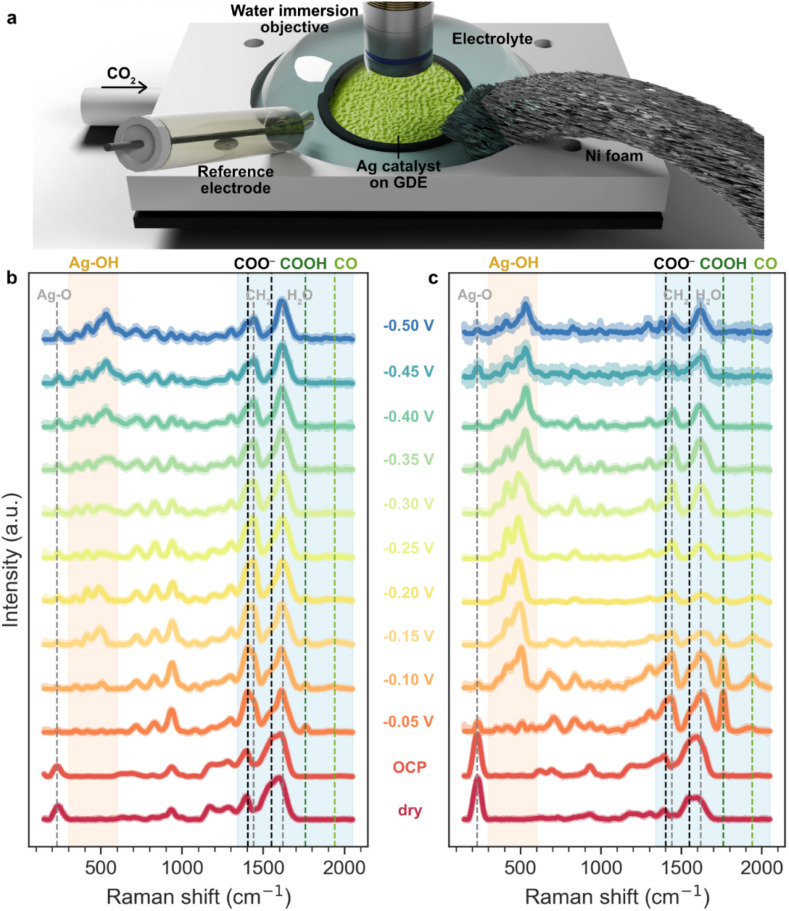
(a) Schematic of the *operando* Raman setup under CO_2_ flow, recorded from −0.05 to −0.5 V *vs.* RHE in 0.1 M NaOH + 0.5 M Na_2_SO_4_ (pH 13). *Operando* Raman spectra at different applied potentials for (b) Ag and (c) AgBa catalysts.

**Fig. 4:**
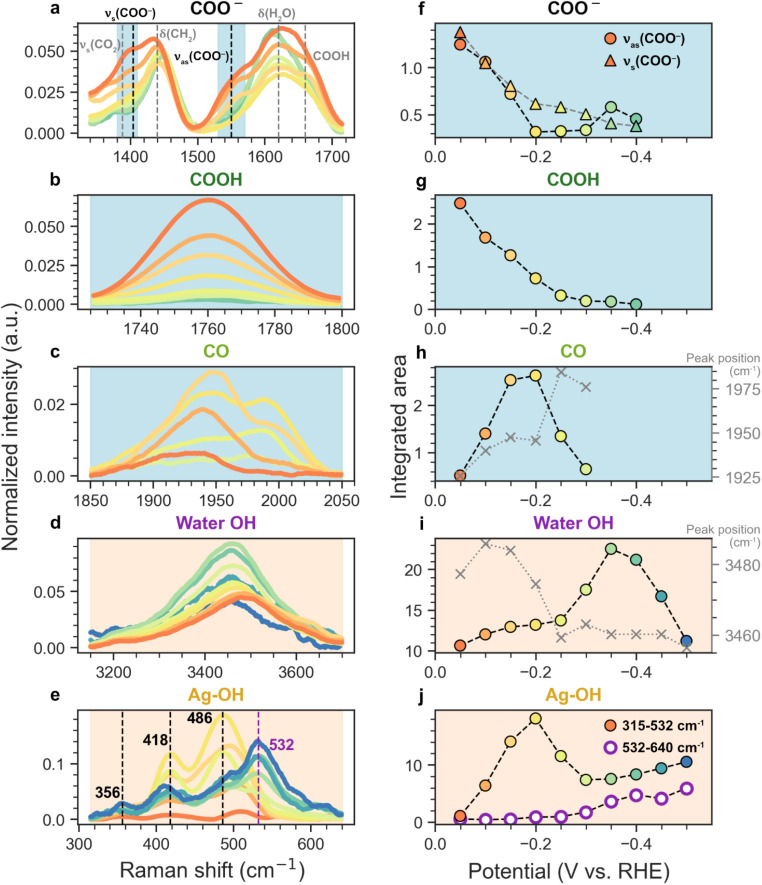
Normalized Raman intensities of AgBa measured in the potential range from −0.05 to −0.5 V *vs.* RHE for (a) *COO^−^, (b) *COOH, (c) *CO, (d) water OH, and (e) Ag–OH, with their corresponding integrated areas shown in (f–j). The shaded backgrounds in (a–e) indicate the range for integration. For clarity, only smoothed signals are plotted here.

To gain deeper insights into the CO_2_RR mechanism, we investigate the catalysts using *operando* Raman spectroscopy. As illustrated in Fig. 3a, the setup uses a 532 nm green laser and a water immersion objective to probe the catalyst-coated GDE immersed in an electrolyte, with CO_2_ supplied from the bottom of the GDE. Although these Raman measurements are performed under CO_2_ flow and cathodic potentials, we emphasize that the conditions are not directly comparable to those in the flow cell in Fig. 2. The first key difference is that the Raman setup employs a static electrolyte with both the working electrode (catalyst-coated GDE) and counter electrode (Ni foam) immersed in the same solution, whereas the flow cell has enhanced electrolyte flow, with the catholyte and anolyte compartments separated by a membrane. In addition, to avoid damaging the objective lens, the Raman electrolyte (pH 13, 0.1 M NaOH + 0.5 M Na_2_SO_4_) is less alkaline than the flow cell conditions (pH 14, 1 M NaOH). For clarity, we structure our discussion in two parts: Fig. 3 presents a qualitative overview of how the Raman spectra evolve with potential, while Fig. 4 dives into specific spectral regions for more quantitative analysis. Fig. 3b and c show the potential-dependent Raman spectra for pure Ag and AgBa, respectively, from before applying potential (dry and OCP) to after cathodic bias (−0.05 V to −0.5 V *vs.* RHE at pH 13).

Spectral acquisition beyond −0.5 V proves challenging, as bubble accumulation on the GDE significantly reduces the signal-to-noise ratio. Although the exact conditions and potentials of the Raman setup are not directly comparable to those in the electrochemical cell, complementary flow-cell measurements at low current density confirm that this regime corresponds to CO production rather than HER (Fig. S17). Hence, the *operando* Raman measurements remain valuable for providing a qualitative view of potential-dependent processes. The Raman spectra for other samples (AgMg, AgCa, AgSr) are presented in Fig. S18. Notably, common to all the samples, there are two regions exhibiting pronounced changes with potential. The low-frequency region (∼300–600 cm^−1^, orange) is dominated by Ag–OH vibrations that grow in intensity under increasingly negative potentials. The higher-frequency region (∼1350–2050 cm^−1^, blue) contains signals associated with CO_2_ reduction intermediates (*e.g.* COO^−^, COOH, and CO), which typically diminish as the potential becomes more cathodic.

However, a direct comparison between Ag (Fig. 3b) and AgBa (Fig. 3c) reveals a striking difference: AgBa shows much stronger signals for CO_2_RR intermediates. At OCP and under dry conditions, neither sample shows peaks for COOH (1760 cm^−1^) or CO (∼1900–2000 cm^−1^). But as soon as cathodic potential is applied at just −0.05 V, AgBa already exhibits a strong COOH peak together with a weaker CO signal, which are both much more prominent than on pure Ag. These features gradually fade with more negative potentials and become nearly undetectable at −0.3 V. COO^−^ species (1404 and 1550 cm^−1^) are also detected as shoulders within the CH_2_ and H_2_O bending regions in the same potential window, which will be discussed further in Fig. 4. Lastly, we observe a prominent Ag–O peak (∼230 cm^−1^), which may originate from surface oxides^35^ or binding with polyvinylpyrrolidone (PVP) stabilizers,^36,37^ together with several strong PVP-related bands across 200–1700 cm^−1^ (Table S4) under dry and OCP conditions. These signals, however, rapidly diminish once a cathodic bias is applied (−0.05 V, Fig. S19), suggesting that PVP largely desorbs under reductive conditions. The disappearance of these features appears to be crucial for catalyst activation, enabling strong and intense Raman signals for following CO_2_RR intermediates. Pure Ag follows a similar trend of declining CO_2_RR-related species (COO^−^, COOH, and CO) under the cathodic sweep, but all the signals are much weaker throughout.

Interestingly, all Group 2-modified samples (AgMg, AgCa, AgSr, AgBa) show enhanced COOH and CO signals relative to pure Ag, with AgBa displaying the strongest intensity. The stronger signals could arise from either physical SERS effects or chemical stabilization of intermediates that increase their surface coverage. Surface roughness from the deposited carbonates may contribute to some degree,^38^ but pyridine adsorption experiments (Fig. S20–S22) show that Ag and AgBa have comparable SERS activity under cathodic conditions, suggesting that physical effects alone cannot account for the difference. Thus, while both physical and chemical factors may play a role, it is likely that Group 2 species help stabilize CO_2_RR intermediates, making them more detectable by Raman spectroscopy. Similar observations have been reported for Group 2-modified Cu catalysts (*e.g.* Cu–Mg, Cu–Ba), where stronger CO Raman signals and higher FEs for C_2+_ products were attributed to increased *CO coverage, which in turn facilitated C–C coupling.^30–33^ By analogy, Group 2 metals on Ag may also enhance *CO binding similarly. However, unlike Cu, CO_2_ reduction on Ag typically terminates at CO as the main product. In this case, stronger *CO binding may hinder CO desorption and thus reduce the overall CO_2_RR performance. This may explain the lower FE_CO_ observed for Group 2-modified catalysts compared to pure Ag (Fig. 2c), apart from the metal carbonates covering some active surface areas of Ag.

### Quantitative analysis of Raman spectra

Although Fig. 3 provides an overview of species present across the chosen potential range, it does not allow us to follow their emergence or disappearance quantitatively. To enable meaningful comparison across spectra with varying absolute intensities, the same normalization and baseline correction procedure was applied to all raw spectra. Prior to the measurement, the objective lens was carefully adjusted to find the optimum working distance that yielded the highest intensity at 100 cm^−1^ (Rayleigh scattering tail), and the position was fixed during subsequent potential-dependent measurements. The normalization of each spectrum was performed by setting its maximum intensity at 100 cm^−1^ to 1 and the minimum intensity across the full recorded range (100–3700 cm^−1^) to 0. Additionally, to avoid artifacts from manual fitting, we subtracted the baseline of all the spectra using the automated Statistics-sensitive Non-linear Iterative Peak-clipping (SNIP) algorithm with a fixed half-window of 55 cm^−1^.^39,40^ Fig. S23–S27 show the spectra before and after baseline subtraction. Further details can be found in the SI.

Fig. 4 shows the normalized Raman spectra and their integrated intensities as a function of applied potential for AgBa, which is selected as a representative example due to its strongest spectral features. Panels a–e highlight the potential-dependent evolution of different species involving COO^−^, COOH, CO, H_2_O, and Ag–OH, while panels f–j display the integrated areas for the corresponding regions. Results for other samples (Ag, AgMg, AgCa, AgSr) are shown in Fig. S28–S31. While absolute intensities may vary between samples, the qualitative trends discussed below are generally observed across all cases unless otherwise noted. As established in Fig. 3, signals associated with hydrogen-containing species (H_2_O, Ag–OH) increase with more negative potentials, while carbon-containing species (COO^−^, COOH, CO) tend to first emerge at mildly cathodic potentials before diminishing afterwards.

#### COO^−^ region (Fig. 4a and f)

The symmetric (*ν*_s_) and antisymmetric (*ν*_as_) stretching vibrations of COO^−^ appear as shoulders at ∼1404 and ∼1550 cm^−1^, respectively, although their interpretation is complicated by neighbouring bands from CH_2_ deformation (∼1440–1450 cm^−1^) and H_2_O bending (∼1620 cm^−1^).^41^ The assignments are consistent with literature values, including the work by Firet and Smith who reported similar COO^−^ features during CO_2_ reduction on Ag using attenuated total reflection Fourier transform infrared spectroscopy (ATR-FTIR).^42^ As the potential sweeps from −0.05 V to more negative values, both COO^−^ shoulders diminish. Integration of the 1380–1410 cm^−1^ and 1530–1570 cm^−1^ regions (shaded blue in Fig. 4a) confirms this trend (Fig. 4f): *ν*_as_(COO^−^) declines sharply and plateaus around −0.2 V, while *ν*_s_(COO^−^) decreases more gradually. The slower decay of *ν*_s_(COO^−^) may result from additional contributions by CO_2_, which has a similar Raman shift for its symmetric stretch (CO_2_: 1388 cm^−1^; COO^−^: 1404 cm^−1^).^43^ We note that formate COO^−^ stretches may also overlap with *ν*_s_(COO^−^) and *ν*_as_(COO^−^) signals used here (Table S5), but since formate is only a minor product (<10% before HER dominates, Fig. 2b), its influence on quantifying the COO^−^ intermediate is expected to be negligible. Due to its minimal spectral overlap and higher reliability, *ν*_as_(COO^−^) is used for quantification in further analysis (Fig. 6).

#### COOH region (Fig. 4b and g)

The C=O stretch of COOH appears as a distinct peak at 1760 cm^−1^, which diminishes steadily as the potential becomes more negative, eventually disappearing at −0.4 V. Integration over 1725–1800 cm^−1^ (Fig. 4g) confirms this decay. The 1760 cm^−1^ feature likely corresponds to monomeric COOH, which matches well with the C=O stretching frequency of aliphatic carboxylic acids.^14^ In contrast, when carboxylic acids form dimers or are hydrogen-bonded (H-bonded), their C=O vibration shifts to lower frequencies.^14^ Hence, we attribute the shoulder at 1660 cm^−1^ (Fig. 4a) as dimeric or H-bonded COOH species, which was also observed by Firet and Smith.^42^

#### CO Region (Fig. 4c and h)

The CO stretching band initially grows in intensity from −0.05 V and peaks at −0.2 V, before disappearing at around −0.3 V (Fig. 4h). Interestingly, during the cathodic sweep, the CO peak gradually shifts to higher wavenumbers (right axis of Fig. 4h), suggesting a transition from more highly coordinated to less coordinated adsorption sites. In general, CO bound to more metal atoms exhibits lower vibrational frequencies: *ν*(CO) on 3-fold hollow < 2-fold bridge < 1-fold atop sites.^44^ However, the exact values could vary considerably depending on the local coordination environment. Experimentally, CO vibrations on Ag have been observed between ∼1800–2100 cm^−1^.^45–50^ Density functional theory (DFT) calculations predict CO stretching frequency on Ag surfaces to be in the range of ∼1895–1961 cm^−1^ for hollow sites, ∼1935–2002 cm^−1^ for bridge sites, and ∼2050–2123 cm^−1^ for atop sites (Table S6).^44,51–53^ Moreover, the CO stretching frequency can be red-shifted in the presence of an electric field by the electrochemical Stark effect, due to an increasing amount of back donation from Ag d states into the CO 2π* antibonding orbital, which weakens the C–O bond and reduces its frequency.^54–56^ At −0.05 V, the CO peak first appears at 1926 cm^−1^ (Fig. 4c and h), likely arising from CO adsorbed at higher-coordinated sites (hollow/bridge). However, as the potential is swept to the negative direction, the CO signal intensifies and shifts to 1948 cm^−1^ by −0.15 V, accompanied by the emergence of a shoulder at 1988 cm^−1^. This suggests a growing contribution from lower-coordination sites (bridge). At −0.2 V, the spectrum shows a clear doublet at 1946 and 1987 cm^−1^ with comparable intensity, indicating the presence of at least two distinct adsorption environments. At −0.25 V, the higher-frequency component becomes dominant with a main peak at 1985 cm^−1^, suggesting a significant presence of bridge CO. Beyond −0.3 V, the CO signals diminish and become too weak for reliable interpretation.

Overall, it appears that CO undergoes a shift from more highly coordinated sites (hollow/bridge) to less coordinated configurations (bridge) as the potential becomes more negative. We hypothesize that this transition is driven by the competitive adsorption from other species (e.g.*H, *OH, *H_2_O), potentially displacing CO from its original sites, particularly *H which is known to favourably adsorb on hollow sites.^53,57,58^ Similar competitive adsorption behaviors have been reported in the literature. Schmitt and Gewirth only observed hollow/bridge-bound CO on pure Ag using SERS, but upon introducing a triazole ligand, they observed the emergence of atop and even physisorbed CO.^47^ However, we note that CO may also exhibit coverage-dependent preferences for adsorption sites in the absence of competitive adsorption.^59,60^

#### H_2_O region (Fig. 4d and i)

**Fig. 5:**
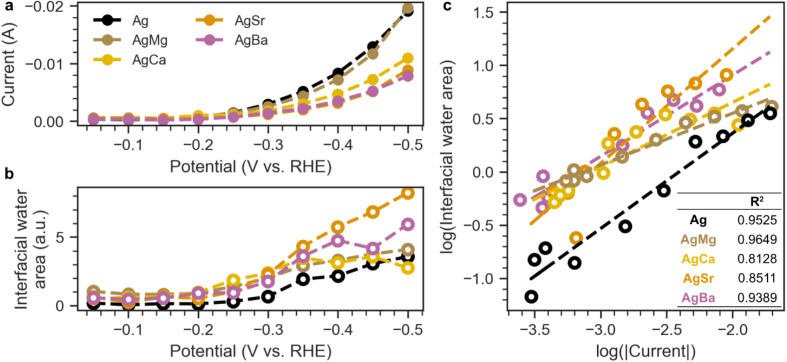
(a) Current and (b) interfacial water area (532–640 cm^−1^) as a functional of potential for all samples (Ag, AgMg, AgCa, AgSr, AgBa). (c) Correlation between log(|current|) and log(interfacial water area).

The O–H stretching band (3150–3700 cm^−1^) grows with increasing cathodic potential, reaching a maximum at −0.35 V before declining thereafter (Fig. 4i). However, for pure Ag, the water intensity continues to increase past −0.35 V with no signs of dropping (Fig. S28). Apart from the intensity changes, the peak position shifts gradually to lower frequencies from 3486 cm^−1^ at −0.1 V to 3456 cm^−1^ at −0.5 V (right axis of Fig. 4i). This indicates a growing population of H-bonded water molecules during the cathodic sweep, which is a common observation even in the absence of CO_2_.^61–63^ More interestingly, the sudden redshift to ∼3460 cm^−1^ at −0.25 V coincides with the sharp increase in water intensity, which also aligns with the spike in the current response (Fig. 5a). Taken together, these observations suggest that this abrupt rise in H-bonded water from −0.25 V is closely related to the onset of HER, which would be discussed extensively in the next section.

#### Ag–OH region (Fig. 4e and j)

The four peaks at 356, 418, 486, and 532 cm^−1^ in the low-frequency region demonstrate two distinct groups of behaviors: the first three already appear at less negative potentials, while the 532 cm^−1^ peak only emerges beyond −0.25 V. The 356–486 cm^−1^ peaks are tentatively assigned to Ag–OH species, as they fall within the expected range of Ag–O modes,^64–68^ while the higher-frequency 532 cm^−1^ peak is attributed to interfacial water (*vide infra*). To better separate their contributions, we integrated the 315–532 cm^−1^ region to represent Ag–OH, and the 532–640 cm^−1^ range to isolate the higher-frequency feature (Fig. 4j).

For the first group of peaks (356, 418, 486 cm^−1^), which is denoted as Ag–OH, another plausible assignment could be Ag–CO modes. However, DFT studies predict Ag–CO vibrations to appear near 200 cm^−1^ (Table S6),^44,51,53^ which would be too low to account for these features. Due to the weak binding of CO on Ag, direct experimental observation of Ag–C vibrations is relatively scarce, with a notable exception from Abe *et al.* who reported a peak at around 160 cm^−1^.^45^ To further rule out CO-related origins, we conducted a control experiment in CO_2_-free, N_2_-purged electrolyte (Fig. S32), where similar peaks (345, 418, 490 cm^−1^) remain observable in the low-frequency region. Interestingly, the integrated intensity of the Ag–OH region (315–532 cm^−1^, Fig. 4j) closely follows the trend of CO (Fig. 4h): both rising from −0.05 V to −0.2 V, then decreasing until −0.3 V. This correlation can be rationalized by the fact that CO and OH^−^ are co-products of *COOH reduction (*COOH + e^−^ → *CO + OH^−^), further supporting our assignment of Ag–OH.

In contrast to other Raman features, the 532 cm^−1^ peak exhibits a distinctly different behavior. As shown by the integrated intensity from 532–640 cm^−1^ (Fig. 4j), this band only begins to emerge at around −0.25 V and continues to rise throughout the cathodic sweep. To visualize the emergence of this feature more clearly, the Raman spectra at each potential are plotted separately in Fig. S33. Notably, this onset at −0.25 V coincides with the abrupt increase in the water OH stretching band (Fig. 4i) and the spike in current (Fig. 5a), strongly suggesting a link between this species and the accumulation of H-bonded water as HER begins. Initially, in the absence of an applied potential, interfacial water molecules are more randomly distributed. However, as the electrode becomes negatively polarized, they reorient into a more ordered structure, with their hydrogen atoms pointing towards the electrode surface, resulting in an “H-down” configuration.^69–74^ When these interfacial water molecules experience strong electrostatic interactions near the negatively charged electrode, their movement is restricted and can result in frustrated rotation (“libration”), giving rise to a broad Raman band between ∼400–700 cm^−1^. Such features have been repeatedly observed in CO_2_-free electrolytes on Ag^64,74–76^ as well as on electrodes such as Au and Pd.^70,71^ Notably, Chen *et al.* observed a broad peak at around 515 cm^−1^ on Ag in 1 M Na_2_SO_4_ in the potential range from −1.30 to −1.60 V *vs.* SCE (−0.29 to −0.59 V *vs.* RHE at pH 13). Upon deuterium substitution, this peak shifted to ∼370 cm^−1^ by a factor of 1.39, confirming its involvement of hydrogen atoms.^74^ In our N_2_-purged control of AgBa without CO_2_ supply (Fig. S32), we also observe a similar broad feature near 450–600 cm^−1^ appearing at more negative potentials, while no appreciable signals are detected in the CO range (1850–2050 cm^−1^), supporting the assignment that its origin is not related to CO_2_. Notably, this 532 cm^−1^ feature appears broader and more pronounced on unmodified Ag (Fig. S28) than on carbonate-modified samples, likely due to the greater polarizability of pure metal to attract more interfacial water under cathodic bias.

To further rule out the possibility that this 532 cm^−1^ feature arises from carbonate residues, we measured potential-dependent Raman spectra on bare Ag in 1 M Na_2_CO_3_ (Fig. S34), which only show the characteristic carbonate C–O stretch at around 1070 cm^−1^, with no observable signals in the 400–700 cm^−1^ region. In addition, Raman spectra of solid Group 2 carbonates are not expected to display peaks in this range.^77^

More importantly, the emergence of this interfacial water band has been correlated with the onset of HER current.^71,74,76^ To illustrate this, we plot the current (Fig. 5a) and the integrated intensity between 532–640 cm^−1^ (Fig. 5b) as a function of potential. For all samples (Ag, AgMg, AgCa, AgSr, AgBa), both the current and the 532 cm^−1^ band begin to rise at around −0.25 V, aligning well with the HER onset. Fig. 5c shows the direct relationship between log(current) and log(interfacial water area), with *R*^2^ values of at least 0.8 across all samples.

Given its strong correlation with HER current, we assign this broad 532 cm^−1^ band to the libration of interfacial water molecules. In this H-down configuration, the shortened distance between the electrode and the hydrogen atoms promotes electron transfer into the antibonding orbital of water, facilitating the Volmer step (* + H_2_O + e^−^ → *H + OH^−^) and initiating HER.^71^ While *H is not directly observed here presumably due to its low polarizability,^78,79^ this increased population of interfacial water could serve as a spectroscopic proxy for growing *H coverage. Since *H preferentially binds to hollow sites,^57^ its accumulation may displace *CO to lower-coordination sites, potentially explaining the CO shift to higher wavenumbers observed in Fig. 4h.

To further examine the relationship between interfacial water and HER, we compared Na^+^ with K^+^ electrolytes. Previous studies using scanning tunneling microscopy (STM) and SEIRAS demonstrated that cations have a strong impact on the interfacial water structure of Au electrodes: “structure-making” cations such as Li^+^ remain more solvated and stay further away from the surface, resulting in an ordered, ice-like layer of interfacial water, whereas “structure-breaking” cations such as K^+^ or Cs^+^ are less hydrated and can approach closer to the electrode, thereby disrupting the H-bonding network of interfacial water.^63^ This ion-specific interfacial water structure correlates well with HER activity, which decreases from Li^+^ to Cs^+^. Based on this, we expect K^+^ to suppress both HER and water-related Raman signals. Indeed, electrochemical flow cell measurements with AgBa in 1 M KOH show that HER remains minor until −600 mA cm^−2^ (40% FE, Fig. S35), whereas in 1 M NaOH HER already dominates at −400 mA cm^−2^ (32% FE, Fig. 2b). *Operando* Raman spectra recorded in 0.1 M KOH + 0.5 M K_2_SO_4_ (Fig. S36–S37) further reveal that the 400–700 cm^−1^ features are strongly diminished relative to Na^+^. Similar suppression of the water libration band has also been reported for Cs^+^ electrolytes.^75,76^ Together, these observations suggest that disrupting the H-bonded interfacial water makes HER less favorable.

### Raman-derived mechanism for CO_2_RR and HER

**Fig. 6:**
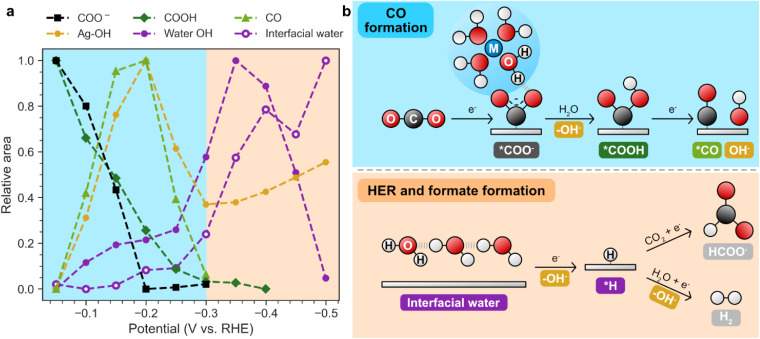
(a) Potential-dependent evolution of different species (COO^−^, COOH, CO, Ag–OH, water OH, and interfacial water) for AgBa. The relative area for each species is obtained by scaling the integrated areas in Fig. 4 to 0–1. (b) Proposed mechanism for CO, formate, and H_2_ formation. CO primarily derives its hydrogen from solvent water, while formate and H_2_ production mostly rely on surface adsorbed *H intermediates, which only become more abundant at increasingly cathodic potentials.

Having examined the emergence of each Raman-active species individually for AgBa, we now compile their potential-dependent profiles into a unified plot to facilitate direct comparison (Fig. 6a). The integrated intensity for each species is scaled to 0–1 to give its relative area. Similar plots for other samples (Ag, AgMg, AgCa, AgSr) are shown in Fig. S38. Fig. 6b summarizes the proposed mechanism consistent with our data and the established literature.

We begin with CO_2_ reduction in Fig. 6a, where a clear progression of the intermediates is observed: CO_2_ → *COO^−^ → *COOH → *CO. Both *COO^−^ and *COOH appear from the initial potential of −0.05 V, but their intensities decline with further cathodic bias. Notably, *COO^−^ diminishes at −0.2 V, while *COOH persists until −0.3 V. This trend supports a stepwise mechanism, where CO_2_ is first activated to form *COO^−^*via* electron transfer (ET), followed by proton transfer (PT) from water to yield *COOH (Fig. 6b). In contrast, *CO does not show a substantial presence initially at −0.05 V. It gradually increases and peaks at −0.2 V before declining until −0.3 V. Importantly, the decrease in *COO^−^/*COOH between −0.05 and −0.2 V mirrors the rise in *CO, providing a strong confirmation that CO is formed by further reducing *COOH (*COOH + e^−^ → *CO + OH^−^) (Fig. 6b).

Beyond −0.3 V, all CO_2_RR-related species only show negligible signals in the spectra, likely due to the growing dominance of the competitive HER, as indicated by the simultaneous spike in current (Fig. 5a). While CO_2_RR may still occur past −0.3 V, its contribution is likely minor on the several-second timescale of our Raman measurements.

We complement this CO_2_RR to CO mechanism with additional insights from the literature (Fig. 6b). First, the activation of CO_2_ to *COO^−^ is generally considered the rate-determining step (RDS) at most potential windows (<−0.7 V *vs.* SHE for Au).^26,80^ Notably, this ET step only involves electrons but not protons as in concerted proton-coupled electron transfer (PCET), implying a weak pH dependence of CO_2_ reduction to CO. This is consistent with electrochemical studies showing that CO current densities on Ag and Au remain nearly constant across acidic and neutral pH when plotted on the SHE scale.^80,81^ Second, at alkaline conditions where water is expected to be the primary proton donor, the subsequent PT step (*COO^−^ → *COOH) likely proceeds *via* an Eley–Rideal mechanism, as *COO^−^ can engage in hydrogen bonding with nearby water molecules *via* its O atom.^24,82^ Since CO formation only requires solvent water as its main hydrogen source (as opposed to *H), this explains why CO is the dominant product at low overpotentials before HER becomes competitive (which requires *H).

Turning to hydrogen-related signals (Fig. 6a), both the OH stretching peak (3150–3700 cm^−1^) and the interfacial water libration band (532–640 cm^−1^) show modest increase from −0.05 to −0.25 V, followed by a sharp rise beyond −0.25 V. For AgBa, the OH stretching signal peaks at −0.35 V before declining, whereas on unmodified Ag both bands continue growing until −0.5 V (Fig. S38). This transition at −0.25 V coincides with the current spike in Fig. 5a, marking the onset of HER. This supports a picture where increasing cathodic bias promotes the accumulation of interfacial water molecules with their hydrogen atoms oriented towards the electrode, which provides a favorable configuration for the Volmer step (H_2_O + e^−^ → *H + OH^−^) (Fig. 6b). Subsequently, HER can proceed *via* the Heyrovsky (*H + H_2_O + e^−^ → H_2_ + OH^−^) or Tafel (*H + *H → H_2_) steps, but experimental and computational studies suggest that the Heyrovsky step is favored on Ag electrodes.^24,83^

The Ag–OH band (Fig. 6a) mirrors the behavior of *CO, increasing up to −0.2 V and decreasing afterwards. This is consistent with both species being products of *COOH reduction (*COOH + e^−^ → *CO + OH^−^) (Fig. 6b). However, unlike *CO, Ag–OH continues to rise slightly beyond −0.3 V, as OH^−^ is also formed during HER *via* both Volmer and Heyrovsky steps.

Overall, our Raman observations of *COO^−^, *COOH, and *CO qualitatively align with the multiscale modelling work of Bell and coworkers, who calculated potential-dependent coverages of different intermediates during CO_2_RR and HER on Ag(110).^24^ While we do not directly observe an increasing amount of *H during the cathodic sweep as predicted by their study, the growing interfacial water features in our spectra provide indirect evidence for its accumulation. Together, our results provide strong experimental support for their proposed mechanism.

Although our discussion centers on CO as the dominant CO_2_RR product, it is also informative to consider the minor product, formate. In principle, formate could be tracked using Raman spectroscopy by monitoring the C–H stretching vibrations (∼2700–3000 cm^−1^). However, strong C–H bands from the PVP stabilizers on Ag particles obscure this region,^36^ making accurate quantification challenging. Nevertheless, the overall C–H signals generally decrease at more cathodic potentials (Fig. S39), suggesting a reduction in formate formation. Mechanistically, quantum mechanical calculations by Goddard and coworkers proposed that formate arises from the reaction of physisorbed CO_2_ with *H (CO_2_ + *H + e^−^ → HCOO^−^),^82^ implying that it competes with HER for surface *H (Fig. 6b). Since *H only becomes abundant at more negative potentials, this explains why formate production typically increases alongside H_2_. For AgBa (Fig. 2b), formate FE rises from 2% at −25 mA cm^−2^ to 28% at −300 mA cm^−2^, accompanied by the growth of H_2_ from 1% to 17% in the same range.

These observations highlight how the availability of *H governs product selectivity: CO dominates at low overpotentials when *H is scarce, while formate and H_2_ emerge at higher overpotentials when *H coverage increases. Recently, Zhang *et al.* generalized this concept of disparate hydrogenation mechanisms to C_2+_ products formed on Cu. Using H_2_O/D_2_O isotope experiments coupled with DFT calculations, they deduced that C–H bonds primarily form *via* *H (Langmuir–Hinshelwood), while O–H bonds mainly originate from direct protonation by solvent water (Eley–Rideal).^84^ This suggests that not all CO_2_RR products compete equally with HER: those less reliant on *H (*e.g.* ethylene) may be favored over those requiring more *H (*e.g.* ethanol) at moderate *H coverage before HER dominates.

While *operando* Raman spectroscopy offers valuable insights into the mechanisms of CO_2_RR and HER, it is important to acknowledge its limitations and consider how complementary techniques can provide a more complete picture of these competing reactions. In this study, we successfully tracked the potential-dependent emergence of key CO_2_RR intermediates (*COO^−^, *COOH, *CO). However, such clear detection is not always guaranteed. Spectroscopy typically only detects species with sufficiently high coverages, which are often associated with rate-limiting steps, and therefore intermediates beyond these steps may be undetected.^27^ Compared to surface-enhanced infrared absorption spectroscopy (SEIRAS), surface-enhanced Raman spectroscopy (SERS) tends to have a lower signal-to-noise ratio and is limited to SERS-active materials such as Ag, Au, and Cu. Due to different sensitivities to dipole moment (SEIRAS) and polarizability (SERS), certain species may be detectable with one technique but not the other.^15^

Moreover, while vibrational spectroscopy is effective at identifying surface-bound intermediates, it typically lacks spatial information about where CO_2_RR and HER occur on the electrode, which is an important aspect that can be better addressed with imaging techniques. For instance, Lu *et al.* used optical coherence tomography (OCT) to visualize the spatial distribution of CO and H_2_ in an optically transparent electrochemical cell with Ag as the catalyst and 3 M KHCO_3_ as both the electrolyte and CO_2_ source. Their results showed that regions of CO formation strongly correlated with triple-phase boundaries, whereas H_2_ production did not exhibit such a relationship.^85^ Similarly, Brosch *et al.* employed confocal laser scanning microscopy (CLSM) with a CO-sensitive fluorescent dye to study a microfluidic Ag-based gas diffusion electrode under CO_2_ flow. They observed that CO was predominantly formed near triple-phase boundaries, while HER occurred throughout the catalyst layer.^86^ These findings highlight the critical role of local CO_2_ availability at triple-phase boundaries in sustaining CO_2_RR and suppressing HER. Interestingly, Brosch *et al.* also noted that electrode flooding did not immediately trigger HER. At low overpotentials, CO_2_RR could still persist even after flooding.^86^ While further investigation is needed to fully understand this phenomenon, a possible explanation is that at low overpotentials, the accumulation of “H-down” interfacial water molecules (which are critical for initiating HER) is still limited, thereby allowing CO_2_RR to remain the favorable pathway.

Finally, we offer a brief perspective on strategies that suppress HER and enhance CO_2_RR activity. A common underlying principle among many of them is to reduce the local availability of water, impeding the reorientation of interfacial water into the H-down configuration favorable for the Volmer step. As an example, coating Cu electrodes with hydrophobic polydimethylsiloxane (PDMS) was demonstrated to hinder water reorientation towards the electrode, as water molecules prefer to hydrogen bond among themselves rather than aligning towards the hydrophobic surface.^62^ Another approach involves using strong H-bond acceptors such as dimethyl sulfoxide (DMSO) as the solvent. DMSO forms strong H-bonds with water molecules, thereby lowering the activity of free water. This led to nearly 100% CO FE in CO_2_RR on Au, with water concentrations up to 3 M.^87^ Similarly, water availability can be reduced by adding highly concentrated salts. In one study, gradually increasing NaClO_4_ concentration from 1 m to 17 m (molality) led to a drop in H_2_ FE from ∼60% to ∼10% at −0.75 V *vs.* RHE on Cu.^88^ While the origin of the cation effect remains debated,^89^ it can also be interpreted through the lens of interfacial water behavior. “Structure-breaking” cations (*e.g.* K^+^, Cs^+^) have weaker hydration free energies compared to “structure-making” cations (*e.g.* Li^+^, Na^+^), and can therefore shed coordinated water more easily and approach closer to the electrode surface.^63,90,91^ This displaces interfacial water molecules and disrupts the favorable water arrangement required for HER. This concept generalizes to organic alkylammonium cations, where it was shown that the CO formation rate is inversely related to the cation-electrode distance.^92^ Collectively, all these examples illustrate a unifying theme: HER suppression is often achieved by hindering the accumulation of H-down water under cathodic polarization. As a final note, water reorientation has broader implications beyond HER. Recent second harmonic generation (SHG) studies by Geiger and coworkers showed that the oxygen evolution reaction (OER) only proceeds after water flips its oxygen atoms towards the electrode,^93,94^ which is essentially the opposite scenario of HER. Clearly, these results suggest that controlling the interfacial water structure is the key to steering electrochemical reaction pathways.

## Conclusions

In this study, we employed *operando* Raman spectroscopy to investigate the mechanism of CO_2_RR and its gradual transition to HER at increasingly cathodic potentials in 0.1 M NaOH + 0.5 M Na_2_SO_4_. Although Group 2-modified Ag catalysts (AgMg, AgCa, AgSr, AgBa) exhibit lower CO_2_RR activity and FE_CO_ than pure Ag, they produce significantly stronger Raman signals for CO_2_RR intermediates. Notably, AgBa displays the most intense features, allowing us to clearly resolve the potential-dependent progression of key intermediates: CO_2_ → *COO^−^ → *COOH → *CO, providing strong experimental support for the proposed CO_2_-to-CO mechanism. At potentials more negative than −0.3 V *vs.* RHE, the signals associated with CO_2_RR intermediates diminish, while a broad band near 532 cm^−1^ emerges. This feature is attributed to the librational mode of interfacial water and highly correlates with the sharp rise in HER current, suggesting a mechanistic link between them. Consistent with prior literature, we propose that as the electrode becomes increasingly negatively charged, interfacial water molecules reorient their hydrogen atoms facing the surface. This “H-down” configuration facilitates water dissociation to yield *H *via* the Volmer step, thereby initiating HER. This framework provides an explanation for the observed product distribution as a function of potential: at low overpotentials, CO formation dominates because it can proceed mostly *via* direct protonation from solvent water with minimal dependence on *H. In contrast, formate and H_2_ production increase at higher overpotentials when *H becomes more abundant. Eventually, as *H coverage rises with increasing cathodic bias, HER becomes the dominant reaction. Overall, HER appears to be closely linked to the buildup of H-down interfacial water. These insights suggest that strategies to suppress HER while promoting CO_2_RR activity should aim at reducing local water availability and disrupting the reorientation of interfacial water to limit *H formation. We note that existing approaches, such as designing hydrophobic electrodes or employing structure-breaking cations (*e.g.* K^+^ as observed in the cation effect), can be understood through the lens of interfacial water behavior.

## Author contributions

K. L. conceived the project, performed electrochemical measurements, and led data analysis. M. A. A. M. conducted *operando* Raman experiments and contributed to data interpretation. N. N. Z., S. S., and R. Z. performed SEM, XRD, and XPS measurements, respectively. M. A. A. M., N. N. Z., A. S., and X. W. provided valuable input on electrochemical measurements. W. S. supervised the project and secured funding. K. L. drafted the manuscript and all authors contributed to the final version of the paper.

## Conflicts of interest

There are no conflicts to declare.

## Supplementary Material

SC-016-D5SC04774A-s001

## Data Availability

Data for this article are available at https://doi.org/10.5281/zenodo.15756630. Supplementary information is available. See DOI: https://doi.org/10.1039/d5sc04774a.
