# Supplementary material for: Mechanistic insights into the competition between electrochemical CO2 reduction and hydrogen evolution on Ag-based electrocatalysts via operando Raman spectroscopy
Source: Chem Sci. 2025 Oct 30;16(48):23160–73. doi: 10.1039/d5sc04774a (PMC12581200; doi:10.1039/d5sc04774a)
Supplement: SC-016-D5SC04774A-s001 [file SC-016-D5SC04774A-s001.pdf]

Supplementary Information (SI) for Chemical Science.
This journal is © The Royal Society of Chemistry 2025

Supplementary Information

# Mechanistic insights into the competition between electrochemical $CO_2$ reduction and hydrogen evolution on Ag-based electrocatalysts via operando Raman spectroscopy

Kinran Lau,‡[a] Muhammad Adib Abdillah Mahbub,‡[a] Nini Zhang,[a] Anirudha Shekhawat,[a] Xin Wang,[a] Sabine Seisel,[a] Ridha Zerdoumi[a] and Wolfgang Schuhmann*[a]

[a] Analytical Chemistry—Center for Electrochemical Sciences (CES), Faculty of Chemistry and Biochemistry, Ruhr University Bochum, Universitsätsstr. 150, 44780, Bochum, Germany.

Figure S1. SEM image of AgMg catalyst....10
Figure S2. SEM image of AgCa catalyst....11
Figure S3. SEM image of AgSr catalyst....12
Figure S4. XPS spectra of Ag showing Ag 3d, C 1s, and O 1s regions....13
Figure S5. XPS spectra of AgBa showing Ag 3d, Ba 3d, C 1s, and O 1s regions....13
Figure S6. XPS spectra of AgSr showing Ag 3d, Sr 3d, C 1s, and O 1s regions....14
Figure S7. Faradaic efficiencies of CO, formate, and $H_2$ from triplicate measurements of Ag. ....14
Figure S8. Faradaic efficiencies of CO, formate, and $H_2$ from triplicate measurements of AgMg....15
Figure S9. Faradaic efficiencies of CO, formate, and $H_2$ from triplicate measurements of AgCa. ....15
Figure S10. Faradaic efficiencies of CO, formate, and $H_2$ from triplicate measurements of AgSr....16
Figure S11. Faradaic efficiencies of CO, formate, and $H_2$ from triplicate measurements of AgBa. ....16
Figure S12. Potential profile for triplicate measurements of Ag. ....17
Figure S13. Potential profile for triplicate measurements of AgMg....18
Figure S14. Potential profile for triplicate measurements of AgCa. ....19
Figure S15. Potential profile for triplicate measurements of AgSr....20
Figure S16. Potential profile for triplicate measurements of AgBa. ....21
Figure S17. Electrochemical performance of AgBa at low current densities (-1 to -25 mA $cm^{-2}$)....22
Figure S18. Operando Raman spectra for AgMg, AgCa, AgSr from -0.05 V to -0.5 V vs. RHE....23
Figure S19. Raman spectra of AgBa recorded before and after applying cathodic potential....24
Figure S20. Raman spectra of Ag in 0.1 M NaOH + 0.5 M $Na_2SO_4$ + 1 mM pyridine....25
Figure S21. Raman spectra of AgBa in 0.1 M NaOH + 0.5 M $Na_2SO_4$ + 1 mM pyridine....26
Figure S22. Average intensity of the pyridine ring-breathing mode at 1008 $cm^{-1}$ for Ag and AgBa....26
Figure S23. Normalized Raman spectra of Ag before and after baseline subtraction....28
Figure S24. Normalized Raman spectra of AgMg before and after baseline subtraction....30
Figure S25. Normalized Raman spectra of AgCa before and after baseline subtraction....32
Figure S26. Normalized Raman spectra of AgSr before and after baseline subtraction....34
Figure S27. Normalized Raman spectra of AgBa before and after baseline subtraction....36
Figure S28. Normalized Raman intensities of Ag measured from −0.05 to −0.5 V vs. RHE. ....37
Figure S29. Normalized Raman intensities of AgMg measured from −0.05 to −0.5 V vs. RHE....38
Figure S30. Normalized Raman intensities of AgCa measured from −0.05 to −0.5 V vs. RHE. ....39
Figure S31. Normalized Raman intensities of AgSr measured from −0.05 to −0.5 V vs. RHE....40
Figure S32. Normalized Raman intensities of AgBa measured in $N_2$-purged electrolyte. ....41
Figure S33. Potential-dependent Raman spectra of AgBa plotted separately. ....42
Figure S34. Potential-dependent Raman spectra of Ag in 1 M $Na_2CO_3$....43
Figure S35. Electrochemical performance of AgBa for $CO_2$RR in 1 M KOH (-50 to -600 mA $cm^{-2}$)....44
Figure S36. Comparison of potential-dependent Raman spectra of AgBa in $Na^+$ and $K^+$ electrolytes....44
Figure S37. Normalized Raman intensities of AgBa measured in 0.1 M KOH + 0.5 M $K_2SO_4$....45
Figure S38. Potential-dependent evolution of different species for AgMg, AgCa, AgSr, and Ag....46
Figure S39. C–H stretching vibrations of AgBa at different applied potentials. ....47

Table S1. Particle diameter of Ag and modified catalysts. ....................................................................9
Table S2. Solubility product ($K_{sp}$) of Group 2 metal hydroxides and carbonates.. ......................................9
Table S3. ICP-MS of Group 2-modified Ag. ......................................................................................9
Table S4. Raman peak assignments for 2-pyrrolidone adsorbed on Ag colloids. ......................................24
Table S5. Comparison of $COO^-$ stretching frequencies for $COO^-$ intermediate and formate. .....................41
Table S6. Literature vibrational frequencies ($cm^{-1}$) of CO on Ag in different coordination environments. 41

# 1. Experimental section

## 1.1. Synthesis of Group 2-modified Ag catalysts

Commercial Ag nanoparticles (Sigma-Aldrich 576832, 99.5%, <100 nm, contains PVP as dispersant) were used as the starting material for precipitating alkaline earth metal carbonates on them. Metal chloride precursors: $MgCl_2.6H_2O$ (Carl Roth, ≥99%, p.a.), $CaCl_2.2H_2O$ (VWR Chemicals, >99%, AnalaR NORMAPUR), $SrCl_2.6H_2O$ (Carl Roth, ≥99%, p.a.), and $BaCl_2.2H_2O$ (Carl Roth, ≥99%, p.a.) were each dissolved in deionized water to prepare 0.1 M $MCl_2$ stock solutions. Potassium carbonate ($K_2CO_3$, ITW Reagents, ≥99.5%, p.a.) was used as the carbonate source. All chemicals were used as received without further purification.

To prepare the modified Ag catalysts, 100 mg of Ag nanoparticles were dispersed in 20 mL of deionized water. Then, 186 µL of the desired 0.1 M $MCl_2$ solution (corresponding to 2 mol% metal relative to Ag) was added, followed by sonication for at least 5 min. Precipitation was initiated by adding 5.56 mL of 5 mM $K_2CO_3$ solution dropwise under vigorous stirring, corresponding to 1.5 equivalents of carbonate relative to the added Group 2 metals. The resulting suspension was centrifuged at 4000 rpm for 20 min (ROTOFIX 32 A, Hettich). The supernatant was decanted, and the solid was washed twice with 40 mL of deionized water, followed by centrifugation under the same conditions. After washing, the modified Ag particles were dried in an oven at 70 °C overnight and collected the following day. The pure Ag particles used in the study were subjected to the same dispersion, washing, and centrifugation procedure, but without the addition of alkaline earth metals and carbonate.

## 1.2. Electrochemical $CO_2$RR measurements

### 1.2.1. Electrode preparation

Catalyst-coated gas diffusion electrodes (GDEs) were prepared by pressure-assisted drop-casting. A suspension containing ~2.3–2.4 mg of catalyst and 1.3 mg of polytetrafluoroethylene (PTFE, Sigma-Aldrich, powder, 1 µm particle size) was dispersed in 2 mL of ethanol. The mixture was sonicated in a bath sonicator for at least 15 min before use. The resulting ink was drop-cast onto 16 mm diameter gas diffusion layers (H23C6, Freudenberg) under vacuum. The final mass loading was in the range of ~1.3–1.5 mg $cm^{-2}$.

### 1.2.2. Electrochemical measurements

Electrochemical $CO_2$ reduction experiments were conducted in a custom flow-through electrolyzer made of poly(methyl methacrylate) (PMMA) operated in a three-electrode configuration using an Autolab potentiostat (PGSTAT302N). The electrolyte used for all measurements was 1 M NaOH (Sigma-Aldrich, ≥98%, p.a., ACS reagent), with 15 mL as the catholyte and 10 mL as the anolyte. The electrolyte was circulated using a Perimax 12 peristaltic pump with a pump speed of about 6 mL $min^{-1}$.

The working electrode (WE) was the catalyst-loaded GDE described above, the counter electrode (CE) was nickel foam, and the reference electrode (RE) was a Ag/AgCl/3 M KCl electrode with a double junction filled with 1 M NaOH. The cathode and anode compartments were separated by an anion

exchange membrane (FAA-3-PK-130, Fumatech). The WE was exposed to a constant flow of $CO_2$ (~20 mL $min^{-1}$) at the back of the GDE, while the catholyte reservoir was continuously purged with $N_2$ (~20 mL $min^{-1}$) to carry the gaseous products to the gas chromatograph (GC). Both gas flow rates were controlled using mass flow controllers (GFC171, AABOLRG).

All measurements were performed using a chronopotentiometric protocol. Initially, a galvanostatic linear sweep was conducted (GLSV) to reach a current density of -25 mA $cm^{-2}$. This was followed by sequential chronopotentiometry (CP) steps at current densities of -25, -50, -100, -200, -300, and -400 mA $cm^{-2}$. Each CP step was held for 422 s, followed by 28 s of galvanostatic electrochemical impedance spectroscopy (GEIS), totalling 450 s per current density. GEIS was performed with a sinusoidal current amplitude of 10% of the applied current, over a frequency range of 100 kHz to 2 Hz. Triplicate measurements were performed to obtain the average and standard deviation.

All potentials are reported versus the reversible hydrogen electrode (RHE), and were calculated using the following relation:

$$E_{RHE} = E_{Ag/AgCl/3M\,KCl} + 0.210 + 0.059 \times pH - iR$$

where:

| | |
|---|---|
| $E_{RHE}$ | potential vs. RHE |
| $E_{Ag/AgCl/3M\,KCl}$ | potential vs. reference electrode of Ag/AgCl/3 M KCl |
| $pH$ | pH is taken as 14 for 1 M NaOH neglecting the activity coefficients in highly concentrated solutions |
| $iR$ | ohmic drop corrected using the resistance measured in GEIS (i: current; R: resistance) |

### 1.2.3. Product analysis

Gas-phase products were quantified using gas chromatography (SRI 8610C, SRI Instruments) equipped with a thermal conductivity detector (TCD) for $H_2$ and a flame ionization detector (FID) with methanizer for CO. The carrier gas was $N_2$ and the column temperature was operated at 90 °C. The GC collected gas from both the catholyte headspace and the $CO_2$ outlet (backside of the GDE) combined through a Y-connector. Samples were injected every 7.5 minutes at the end of each applied current density during the CP measurement, and the product separation was completed by the GC in 6 min.

The Faradaic efficiency (FE) for a particular gaseous product (*n*) was calculated using:

$$FE_n = \frac{x_n \cdot z_n \cdot f \cdot F}{V_m \cdot i_t} \times 100\%$$

where:

| | |
|---|---|
| $x_n$ | concentration of product *n* in vol% (i.e. ppm measured in GC multiplied by $10^{-6}$) |
| $z_n$ | number of electrons transferred for product *n* (2 for both $H_2$ and CO) |

| | |
|---|---|
| $f$ | gas flow rate (L $s^{-1}$) |
| $F$ | Faraday constant (94685 C $mol^{-1}$) |
| $V_m$ | molar volume of an ideal gas at 25 °C (24.5 L $mol^{-1}$) |
| $i_t$ | current at injection time (A) |

Liquid-phase products were quantified using high-performance liquid chromatography (HPLC, Dionex ICS-5000, ThermoFisher). The system was equipped with an ion-exclusion column (Aminex HPX-87H, Bio-Rad), a diode array detector at 220 nm, and a refractive index detector (RefractoMax520). At the end of GLSV and after each CP step, a 540 µL aliquot was sampled from the catholyte. Prior to HPLC injection, 440 µL of the aliquot were acidified by mixing with 110 µL of 2.5 M $H_2SO_4$ and subsequently filtered using a 0.2 µm polyethersulfone syringe filter (Filtropur S, Sarstedt). During HPLC analysis, 4 mM $H_2SO_4$ was used as the eluent at a flow rate of 0.6 mL $min^{-1}$, with the column maintained at 70 °C. Formic acid/formate was detected and quantified using the refractive index detector.

The FE for a particular liquid product (*n*) was calculated using:

$$FE_n = \frac{m_n \cdot z_n \cdot F}{i_t \cdot t} \times 100\%$$

where:

| | |
|---|---|
| $m_n$ | no. of moles of product *n* produced at a particular current density |
| $z_n$ | number of electrons transferred for product *n* (2 for formate) |
| $F$ | Faraday constant (94685 C $mol^{-1}$) |
| $i_t$ | current at a particular current density (A) |
| $t$ | measurement duration for a particular current density (450 s) |

## 1.3. Operando Raman measurements

Operando Raman spectroscopy was performed using a Lab-RAM HR Raman microscopy system (Horiba Jobin Yvon HR550) equipped with a 532 nm green laser, a water-immersion objective (Olympus LUMFL, 60×, numerical aperture = 1.10), a monochromator (grating of 1200 grooves/mm), and a Synapse CCD detector. A custom PTFE open cell was employed, where $CO_2$ flowed from the bottom side of the GDE. The three-electrode configuration consisted of the GDE working electrode, a Ag/AgCl/3 M KCl reference electrode, and a Ni foam counter electrode. The electrolyte comprised 0.1 M NaOH (Sigma-Aldrich, ≥98%, p.a., ACS reagent) and 0.5 M $Na_2SO_4$ ($Na_2SO_4$ $10H_2O$, VWR Chemicals, 99.8%, AnalaR NORMAPUR). Schematic of the setup was illustrated in Figure 3a as well as in our previous publication.[1]

The GDE was prepared similarly to those used in flow cell electrochemical measurements by drop-casting a catalyst ink containing ~3.0 mg of catalyst and 1.5 mg of PTFE dispersed in 2 mL of ethanol onto a 20 mm diameter H23C6 carbon paper (Freudenberg). Raman measurements began with calibrating the Rayleigh scattering peak at 0 $cm^{-1}$. Initial spectra were recorded under dry conditions by adjusting the objective lens to the optimal working distance such that the intensity at 100 $cm^{-1}$ was the

strongest. After introducing the electrolyte, the objective lens was slightly re-focused to maintain the high intensity at 100 $cm^{-1}$. Once focused, the position was fixed, and no further adjustments were made throughout the potential-dependent measurements. Raman spectra were collected in the range of 100–3700 $cm^{-1}$. Each spectrum was acquired at least for 10 s after applying the potential to ensure the current had stabilized. Operando Raman measurements were performed from open-circuit potential (OCP) down to –0.5 V vs. RHE at pH 13, using a Gamry REF600 potentiostat.

Raw spectra were first scaled such that the minimum intensity was set to 0 and the maximum intensity at 100 $cm^{-1}$ was normalized to 1. Baseline subtraction was then performed using the SNIP (Statistics-sensitive Non-linear Iterative Peak-clipping) algorithm implemented in the pybaselines Python package, using a half-window size of 55 $cm^{-1}$. The resulting spectra were smoothed using a Savitzky–Golay filter with a window size of 40 and a polynomial order of 3, implemented using scipy.

The following spectral regions were integrated to track different species:

- Ag–OH: 315–532 $cm^{-1}$
- Interfacial water: 532–640 $cm^{-1}$
- Symmetric stretch of $COO^-$: 1380–1410 $cm^{-1}$
- Asymmetric stretch of $COO^-$: 1530–1570 $cm^{-1}$
- COOH: 1725–1800 $cm^{-1}$
- CO: 1850–2050 $cm^{-1}$
- Water O–H stretching: 3150–3700 $cm^{-1}$

## 1.4. Characterization

### 1.4.1. Scanning electron microscopy (SEM)

SEM images were acquired using a Quanta 3D FEG scanning electron microscope (FEI) operated at 30.0 kV in high-vacuum mode. Samples were prepared by drop-casting the catalyst ink onto a 16 mm diameter GDE in the same way as the electrochemical measurements.

### 1.4.2. X-ray diffraction (XRD)

XRD patterns were recorded using a Bruker D8 Discover diffractometer equipped with a Cu Kα radiation source (λ = 1.5418 Å). Measurements were carried out in the 2θ range of 5° to 90° with a step size of 0.01°. The samples were prepared by drop-casting the catalyst ink onto a 16 mm diameter GDE following the same procedure used for electrochemical testing.

### 1.4.3. X-ray photoelectron spectroscopy (XPS)

XPS was carried out using an AXIS Nova spectrometer (Kratos Analytical) equipped with a monochromatic Al Kα X-ray source (1487 eV, 15 mA emission current), operating at a chamber pressure of approximately $10^{-8}$ Torr. Powder samples were pressed onto conductive adhesive tape for analysis. High-resolution spectra were acquired in fixed transmission mode with a pass energy of 20 eV, and charge compensation was applied using an electron flood gun. For all samples, wide survey spectra were acquired, along with high-resolution scans of the Ag 3d, O 1s, and C 1s regions. For samples containing Group 2 elements, the corresponding core-level regions (e.g. Ba 3d) were also recorded. The binding energies of the core-level spectra were calibrated with the C 1*s* signal at 284.8 eV. Peak analysis was performed using the ESCApe software (Kratos) with a Shirley background subtraction. For quantification, the used relative sensitivity factors for Ag 3d, Ba 3d, and Sr 3d were 5.99, 12.45, and 1.84 respectively.

### 1.4.4. Inductively coupled plasma mass spectrometry (ICP-MS)

ICP-MS was performed using an iCAP RQ instrument (Thermo Fisher Scientific) in kinetic energy discrimination (KED) mode with helium as the collision gas and an argon plasma. Approximately 3–5 mg of catalyst powder was dissolved in 300 µL of 69% nitric acid (ROTIPURAN® Supra) by heating the mixture to ~100 °C in an Eppendorf tube. The resulting solution was then diluted to a total volume of 10 mL with ultrapure water. From this stock solution, three dilutions (0.01%, 0.1%, and 1%) were prepared. For each dilution, 300 µL of 69% nitric acid was added and the solution was made up to 10 mL, resulting in a final acid concentration of approximately 2.07% nitric acid. These three dilutions were analyzed by ICP-MS to ensure that the elemental concentrations fell within the calibration range (0–100 ppb). Calibration standards were prepared accordingly for quantitative analysis.

## 2. Supplementary data

**Table S1.** Particle diameter of Ag and modified catalysts.

| Sample | Average particle diameter (nm) | σ (nm) | N |
|---|---|---|---|
| Ag | 56.7 | 20.2 | 191 |
| AgMg | 62.2 | 19.7 | 146 |
| AgCa | 62.9 | 21.0 | 118 |
| AgSr | 61.3 | 17.5 | 215 |
| AgBa | 69.2 | 22.4 | 178 |

**Table S2.** Solubility product ($K_{sp}$) of Group 2 metal hydroxides and carbonates. All data from CRC Handbook of Chemistry and Physics, 87th Edition [2], unless otherwise specified.

| Metal | $M(OH)_2$ / ($mol^3\ L^{-3}$) | $MCO_3$ / ($mol^2\ L^{-2}$) |
|---|---|---|
| Mg | $5.61 \times 10^{-12}$ | $6.82 \times 10^{-6}$ |
| Ca | $5.02 \times 10^{-6}$ | $3.36 \times 10^{-9}$ |
| Sr | $3.2 \times 10^{-4}$ [3] | $5.60 \times 10^{-10}$ |
| Ba | $2.55 \times 10^{-4}$ | $2.58 \times 10^{-9}$ |

**Table S3.** ICP-MS of Group 2-modified Ag. Concentrations correspond to the original (undiluted) solutions, where dilution factors have already been accounted for.

| Sample | Mass (mg) | Element | ppb | µmol $L^{-1}$ | Group 2/Ag ratio |
|---|---|---|---|---|---|
| **AgMg** | 5.08 | Mg | 43.84 | 1.803 | **0.04%** |
| | | Ag | 445160 | 4126 | |
| **AgCa** | 4.97 | Ca | 24.79 | 0.618 | **0.01%** |
| | | Ag | 494080 | 4579 | |
| **AgSr** | 2.99 | Sr | 1933 | 22.058 | **0.9%** |
| | | Ag | 261950 | 2428 | |
| **AgBa** | 2.96 | Ba | 135.1 | 0.984 | **0.04%** |
| | | Ag | 270480 | 2507 | |

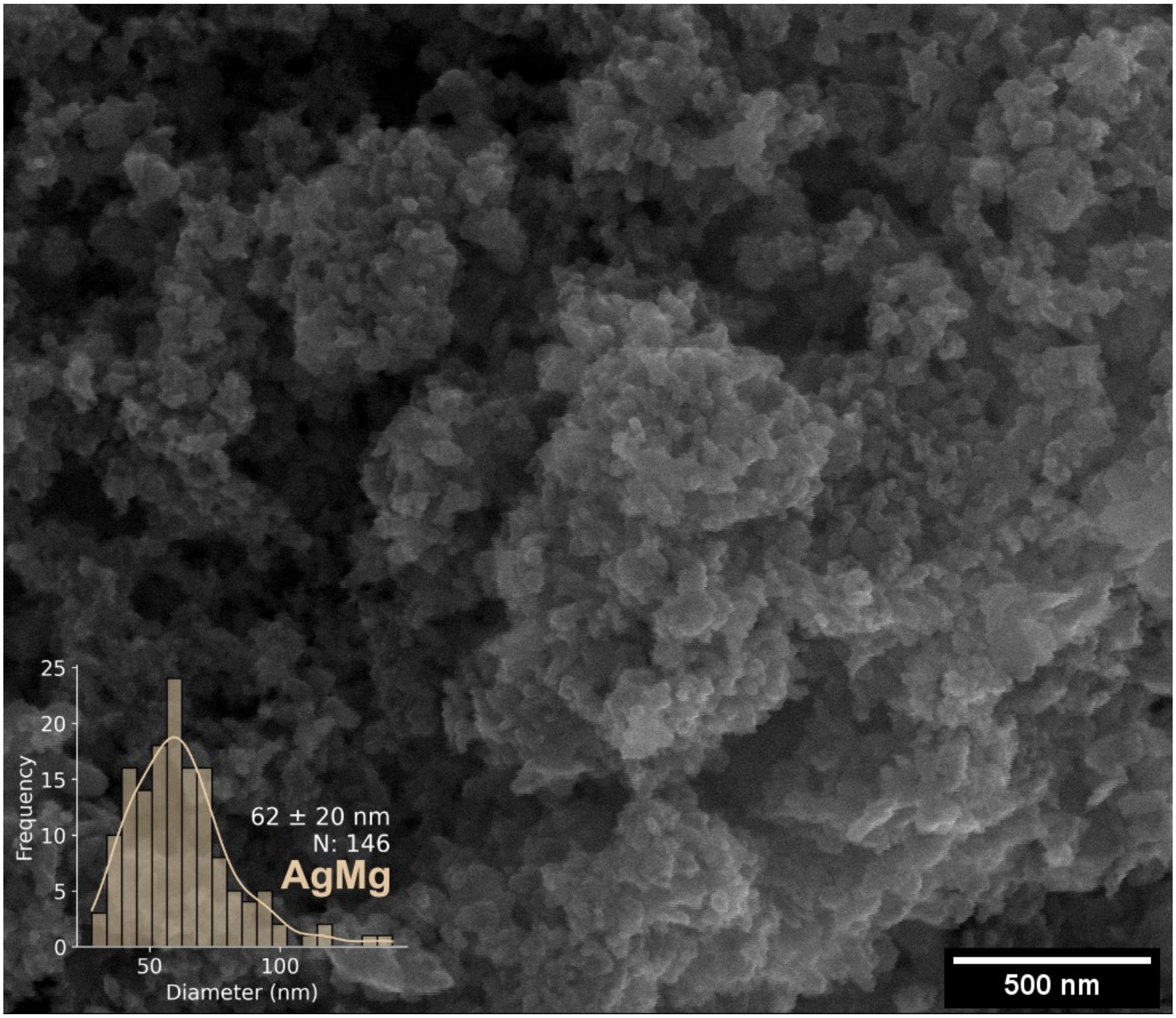


**Figure S1.** SEM image of AgMg catalyst.

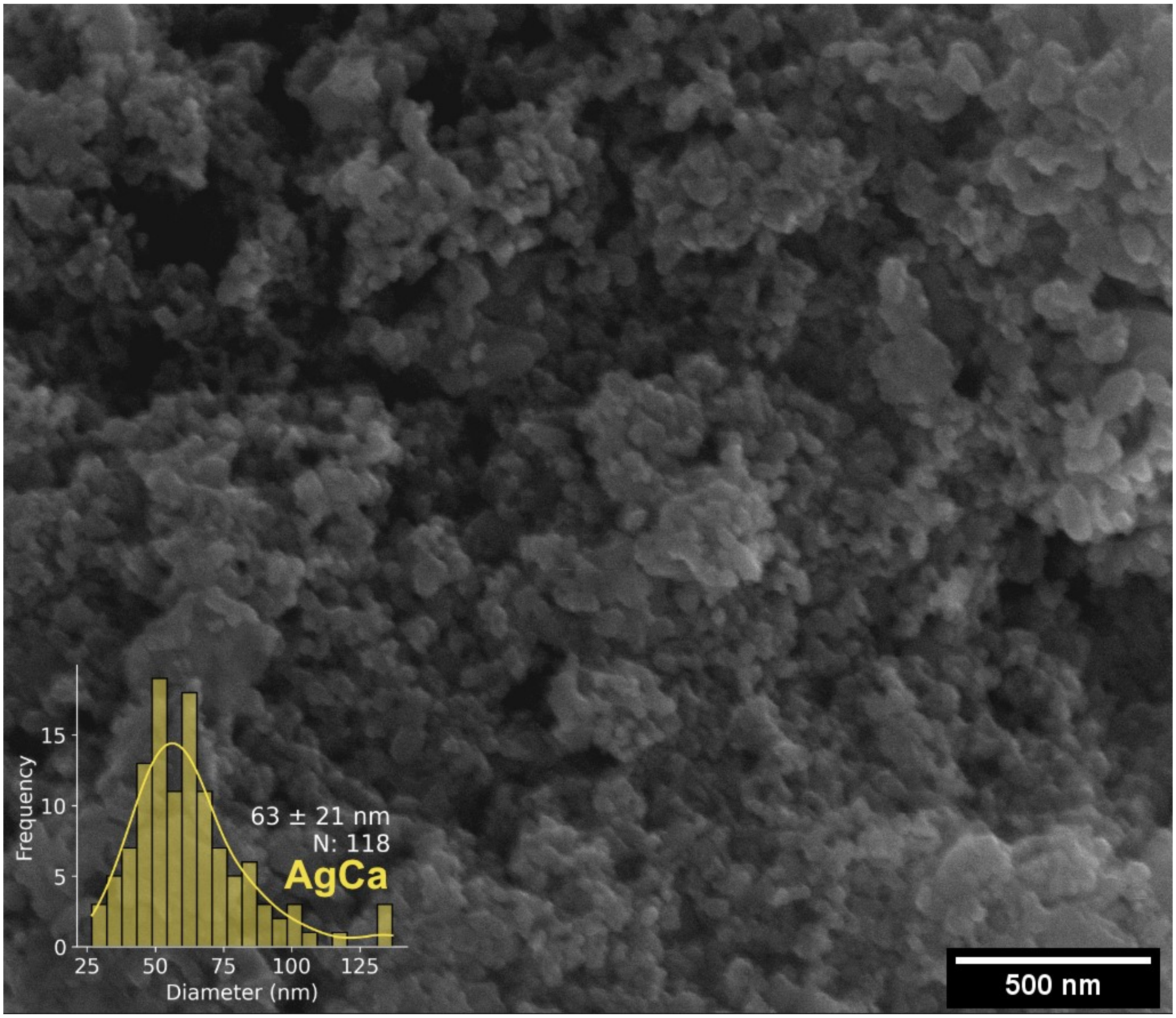


**Figure S2.** SEM image of AgCa catalyst.

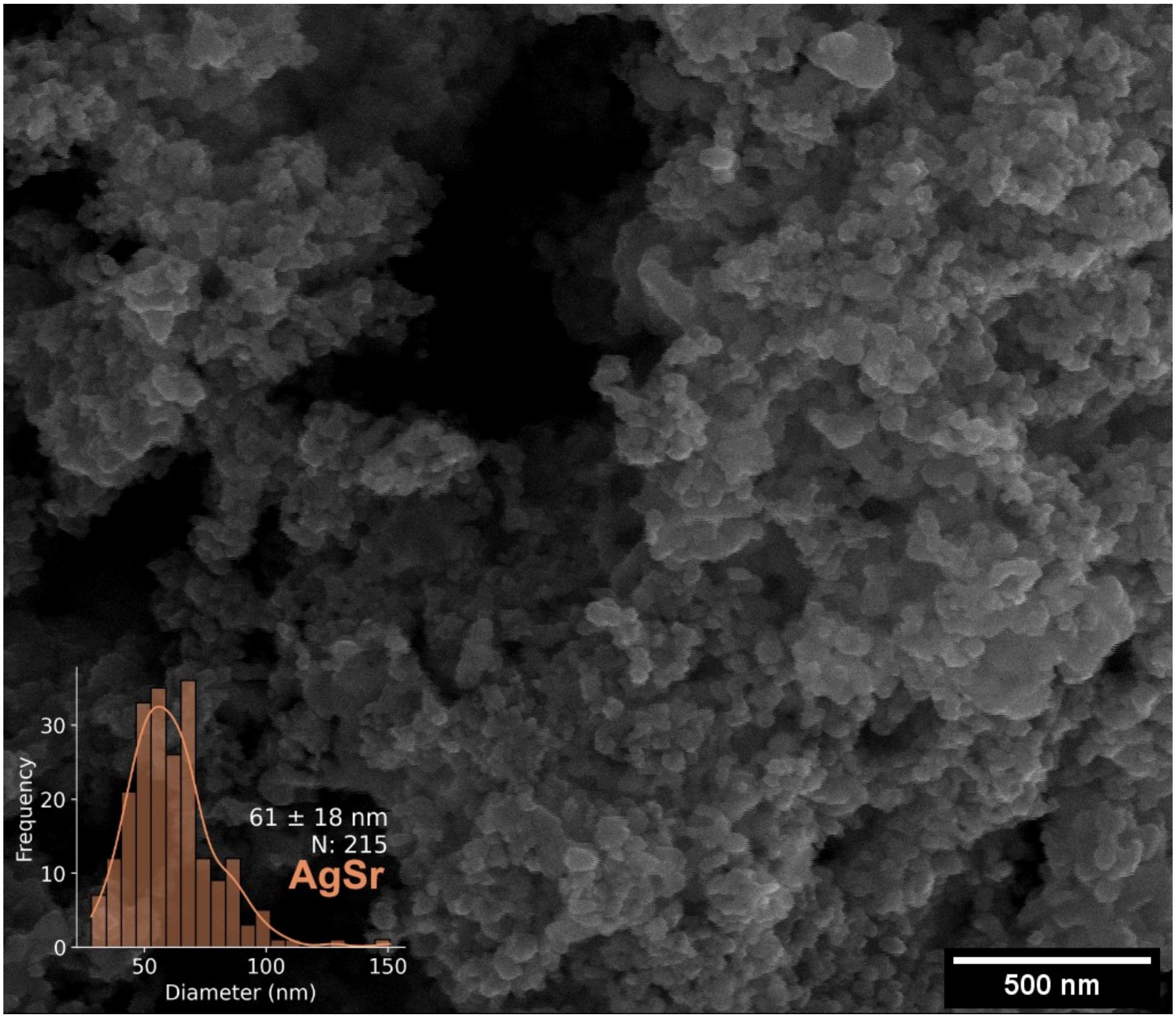


**Figure S3.** SEM image of AgSr catalyst.

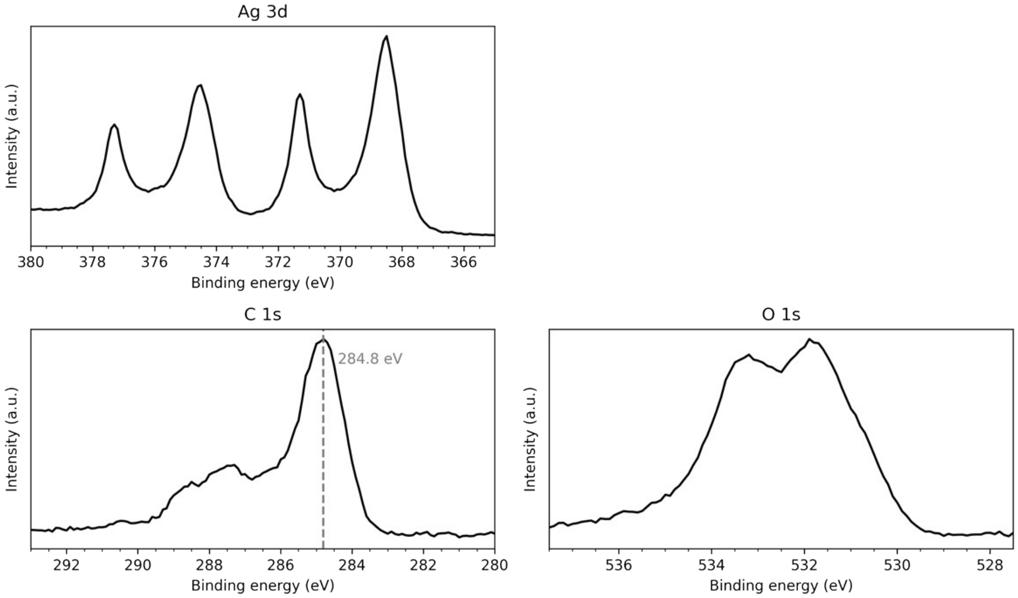


**Figure S4.** High resolution XPS spectra of unmodified Ag nanoparticles showing Ag 3d, C 1s, and O 1s regions. In the Ag $3d_{5/2}$ region, an additional peak appears at ~371 eV alongside the expected peak at around 368.5 eV peak. The additional peak cannot be attributed to silver oxides or metallic silver ($Ag_2O$: 367.9 eV; AgO: 367.3 eV). It is likely due to partial surface charging of the Ag nanoparticles caused by the presence of PVP stabilizers, which might form an insulating layer.

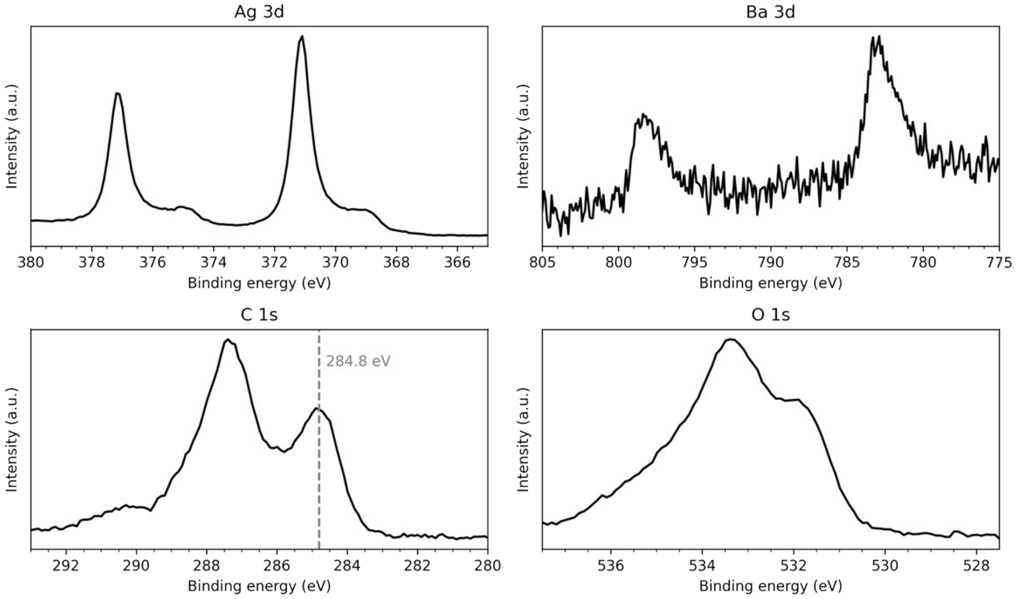


**Figure S5.** High-resolution XPS spectra of AgBa showing Ag 3d, Ba 3d, C 1s, and O 1s regions. In the Ag $3d_{5/2}$ region an additional peak appears at ~371 eV alongside the expected peak at around 368.5 eV. This feature is also present in the commercial Ag starting material (Figure S4) and cannot be attributed to silver oxides or metallic silver ($Ag_2O$: 367.9 eV; AgO: 367.3 eV). It is likely due to partial surface charging of the Ag nanoparticles caused by the presence of PVP stabilizers, which might form an insulating layer. The Ba $3d_{5/2}$ peak appears at 783 eV, which is also approximately 3 eV higher than the expected value for $BaCO_3$ (780 eV), possibly due to similar charging effects. The quantification of the Ba/Ag ratio (0.55%) was performed by integrating the Ba 3d region and normalizing to the Ag 3d region using a Shirley background and appropriate sensitivity factors.

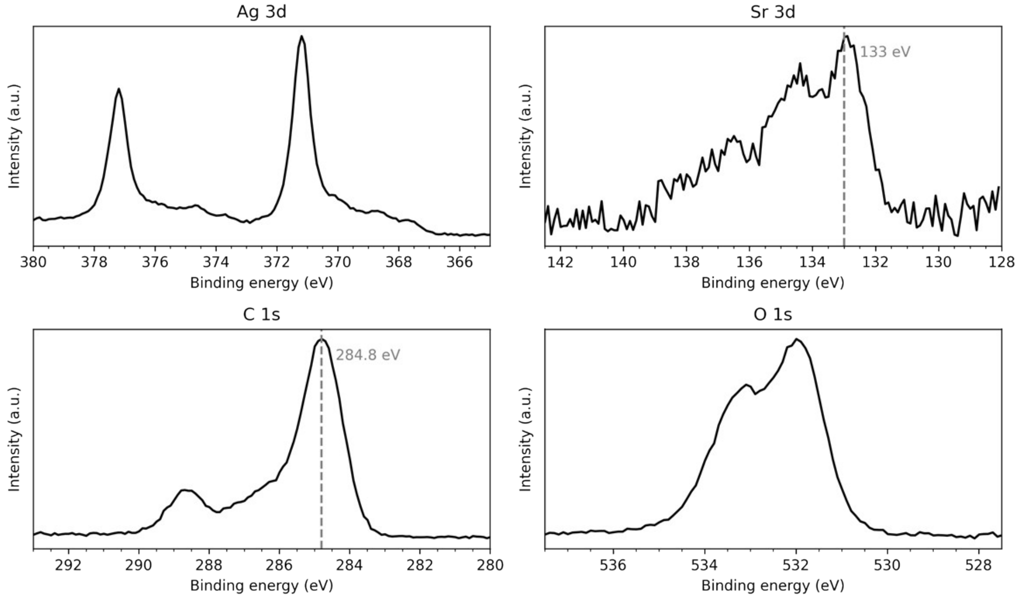


**Figure S6.** High-resolution XPS spectra of AgSr showing Ag 3d, Sr 3d, C 1s, and O 1s regions. In the Ag $3d_{5/2}$ region an additional peak appears at ~371 eV alongside the expected peak at around 368.5 eV. This feature is also present in the commercial Ag starting material (Figure S4) and cannot be attributed to silver oxides or metallic silver ($Ag_2O$: 367.9 eV; AgO: 367.3 eV). It is likely due to partial surface charging of the Ag nanoparticles caused by the presence of PVP stabilizers, which might form an insulating layer. The Sr $3d_{5/2}$ peak appears at around 133 eV, which is the expected value for SrO and $SrCO_3$. The quantification of the Sr/Ag ratio (1.91%) was performed by integrating the Sr 3d region and normalizing to the Ag 3d region using a Shirley background and appropriate sensitivity factors.

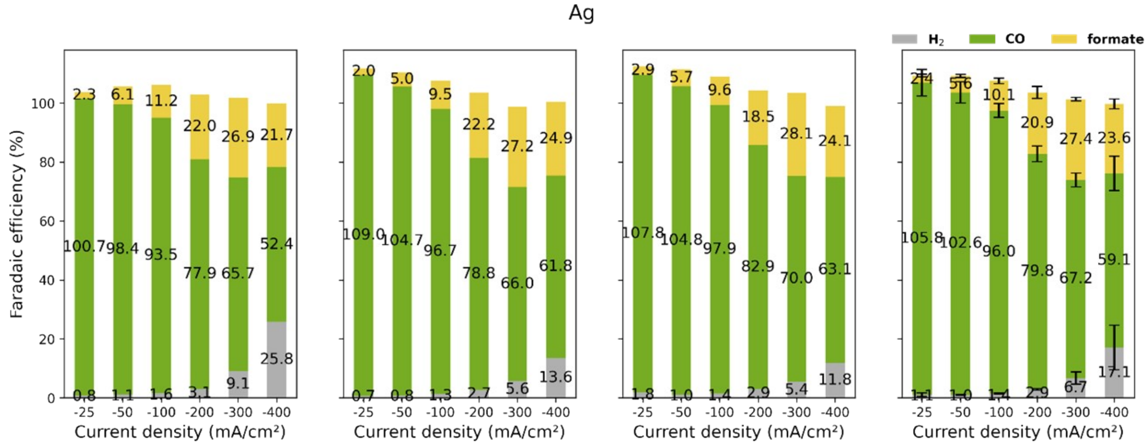


**Figure S7.** Faradaic efficiencies of CO, formate, and $H_2$ from triplicate measurements of Ag over a range of current densities (-25 to -400 mA cm$^{-2}$) in 1 M NaOH (pH 14). Error bars represent ±1 standard deviation.

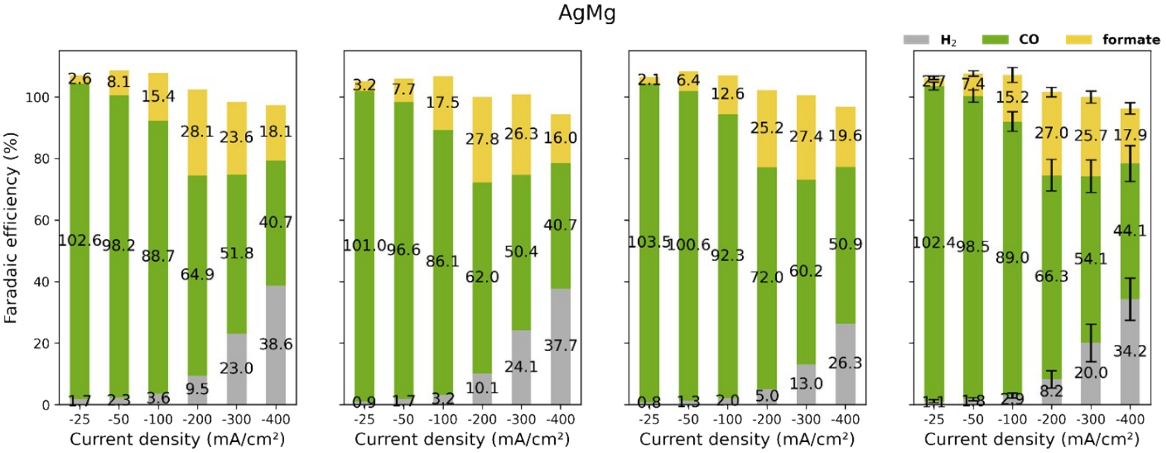


**Figure S8.** Faradaic efficiencies of CO, formate, and $H_2$ from triplicate measurements of AgMg over a range of current densities (-25 to -400 mA cm$^{-2}$) in 1 M NaOH (pH 14). Error bars represent ±1 standard deviation.

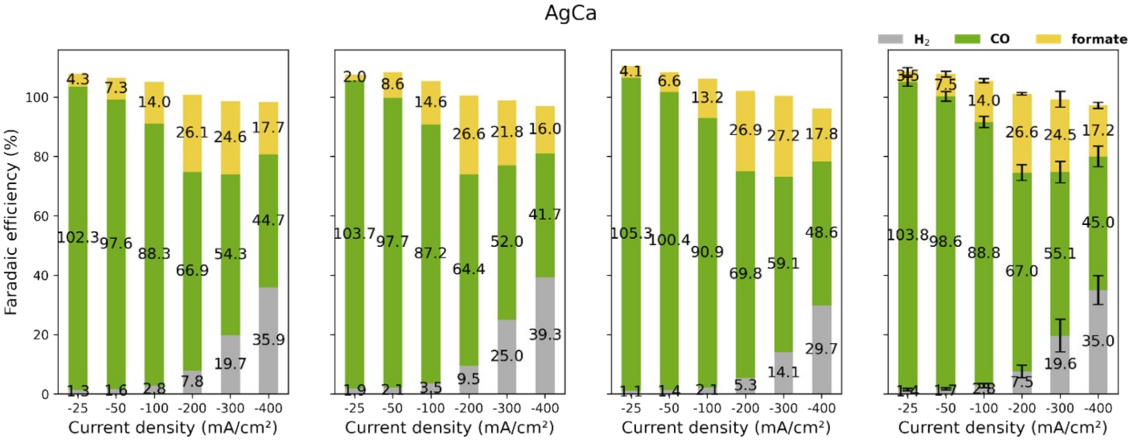


**Figure S9.** Faradaic efficiencies of CO, formate, and $H_2$ from triplicate measurements of AgCa over a range of current densities (-25 to -400 mA cm$^{-2}$) in 1 M NaOH (pH 14). Error bars represent ±1 standard deviation.

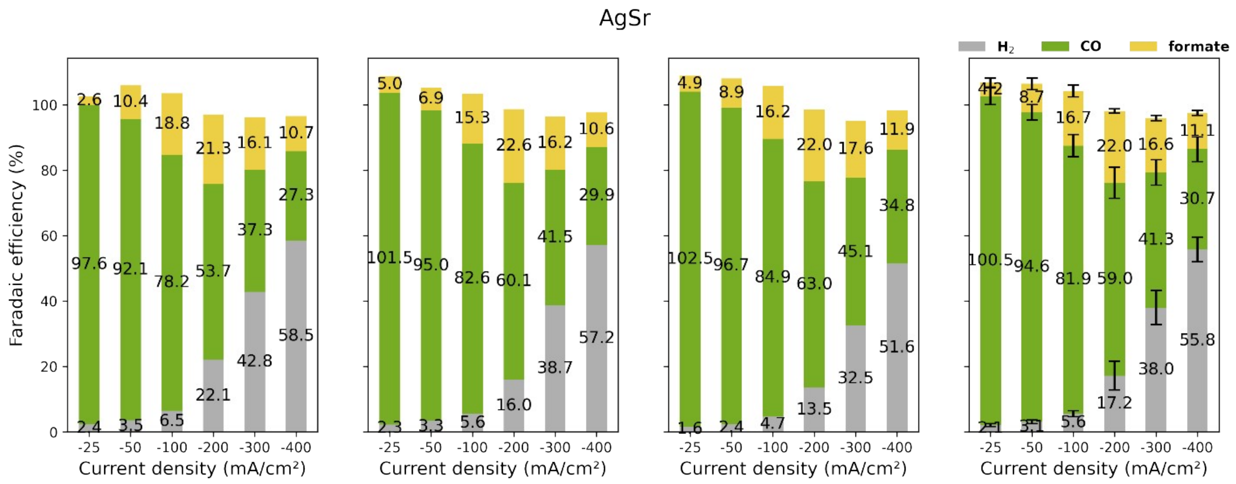


**Figure S10.** Faradaic efficiencies of CO, formate, and $H_2$ from triplicate measurements of AgSr over a range of current densities (-25 to -400 mA cm$^{-2}$) in 1 M NaOH (pH 14). Error bars represent ±1 standard deviation.

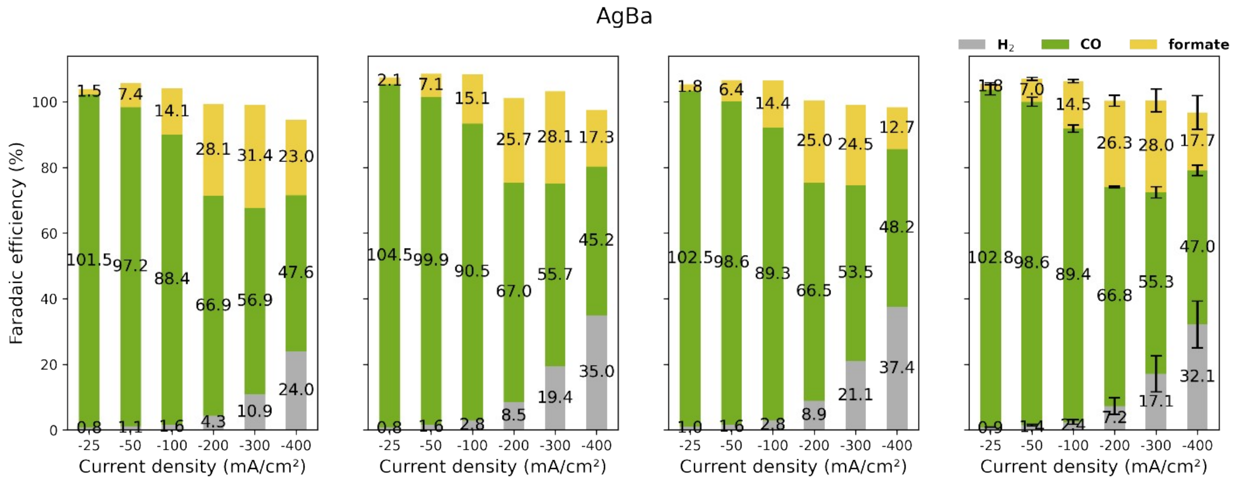


**Figure S11.** Faradaic efficiencies of CO, formate, and $H_2$ from triplicate measurements of AgBa over a range of current densities (-25 to -400 mA cm$^{-2}$) in 1 M NaOH (pH 14). Error bars represent ±1 standard deviation.

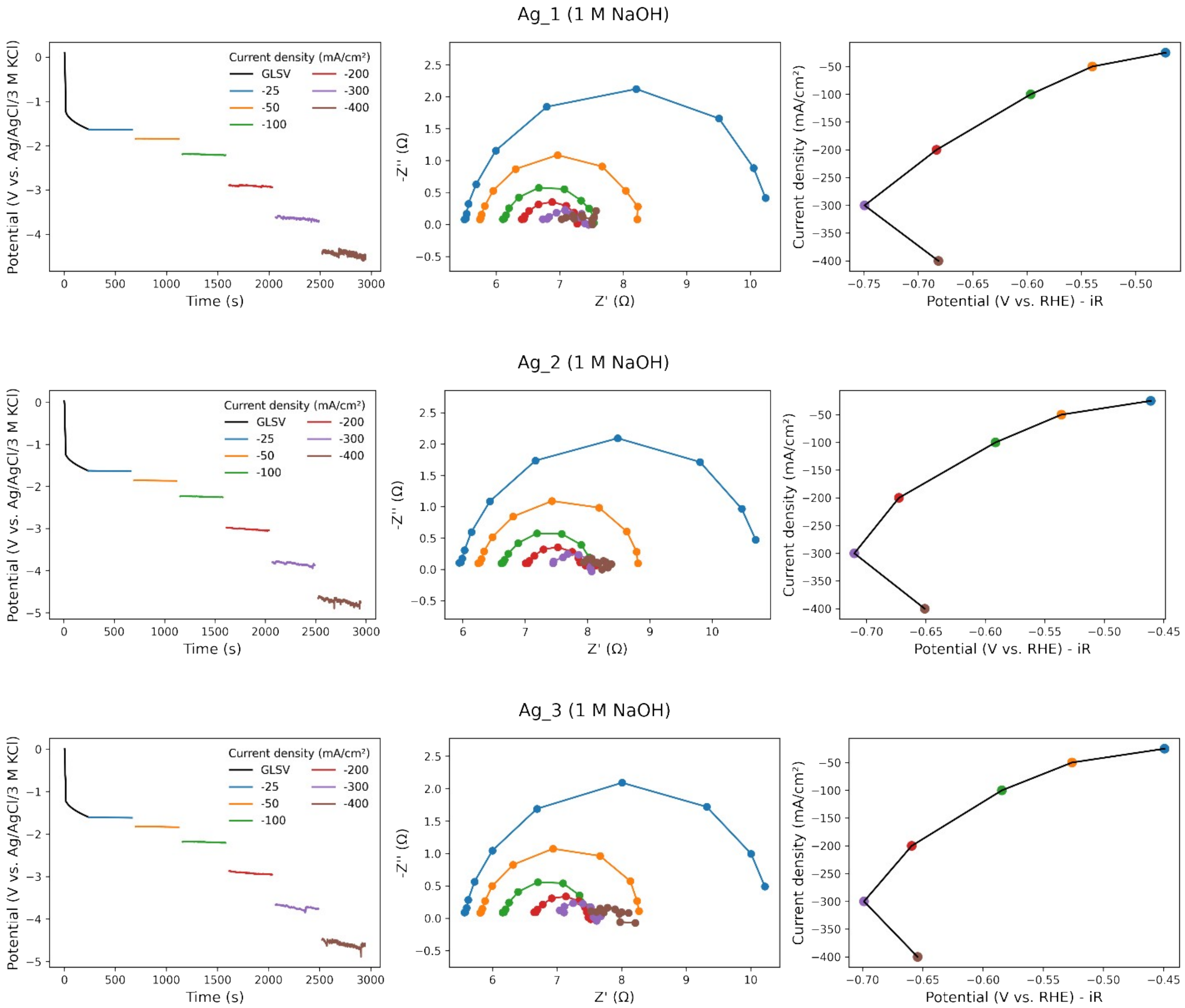


**Figure S12.** Potential profile for triplicate measurements of Ag. Left: Uncompensated potential vs. Ag/AgCl/3 M KCl as a function of time. Middle: Galvanostatic electrochemical impedance spectroscopy (GEIS) measured at the end of each current step. Right: Current density (-25 to -400 mA $cm^{-2}$) and iR-corrected potential vs. RHE. Note that the value at -400 mA $cm^{-2}$ may be unreliable due to vigorous bubble formation interfering with accurate resistance measurement. Average potentials are shown in Figure 2d.

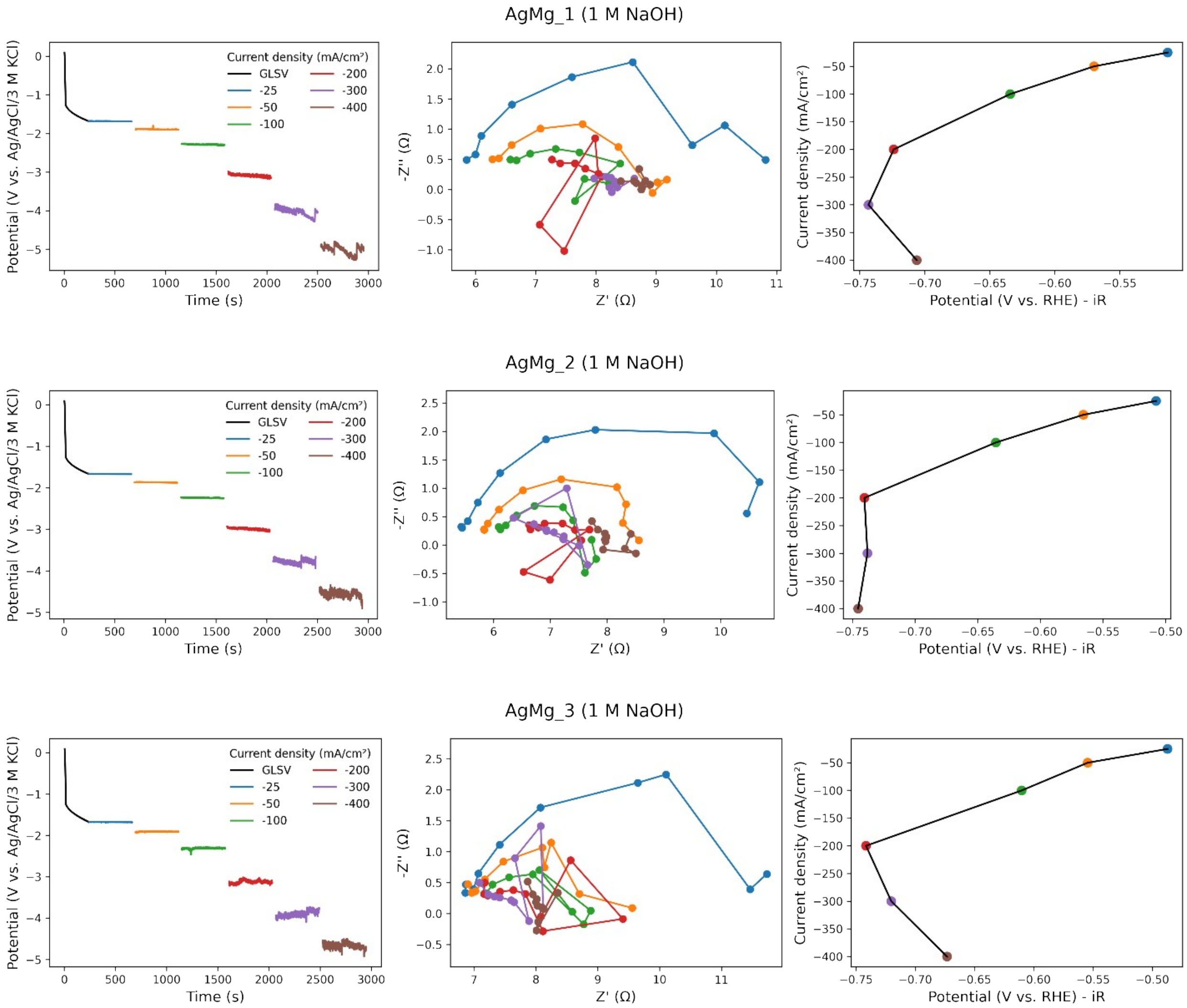


**Figure S13.** Potential profile for triplicate measurements of AgMg. Left: Uncompensated potential vs. Ag/AgCl/3 M KCl as a function of time. Middle: Galvanostatic electrochemical impedance spectroscopy (GEIS) measured at the end of each current step. Right: Current density (-25 to -400 mA $cm^{-2}$) and iR-corrected potential vs. RHE. Note that the value at -400 mA $cm^{-2}$ may be unreliable due to vigorous bubble formation interfering with accurate resistance measurement. Average potentials are shown in Figure 2d.

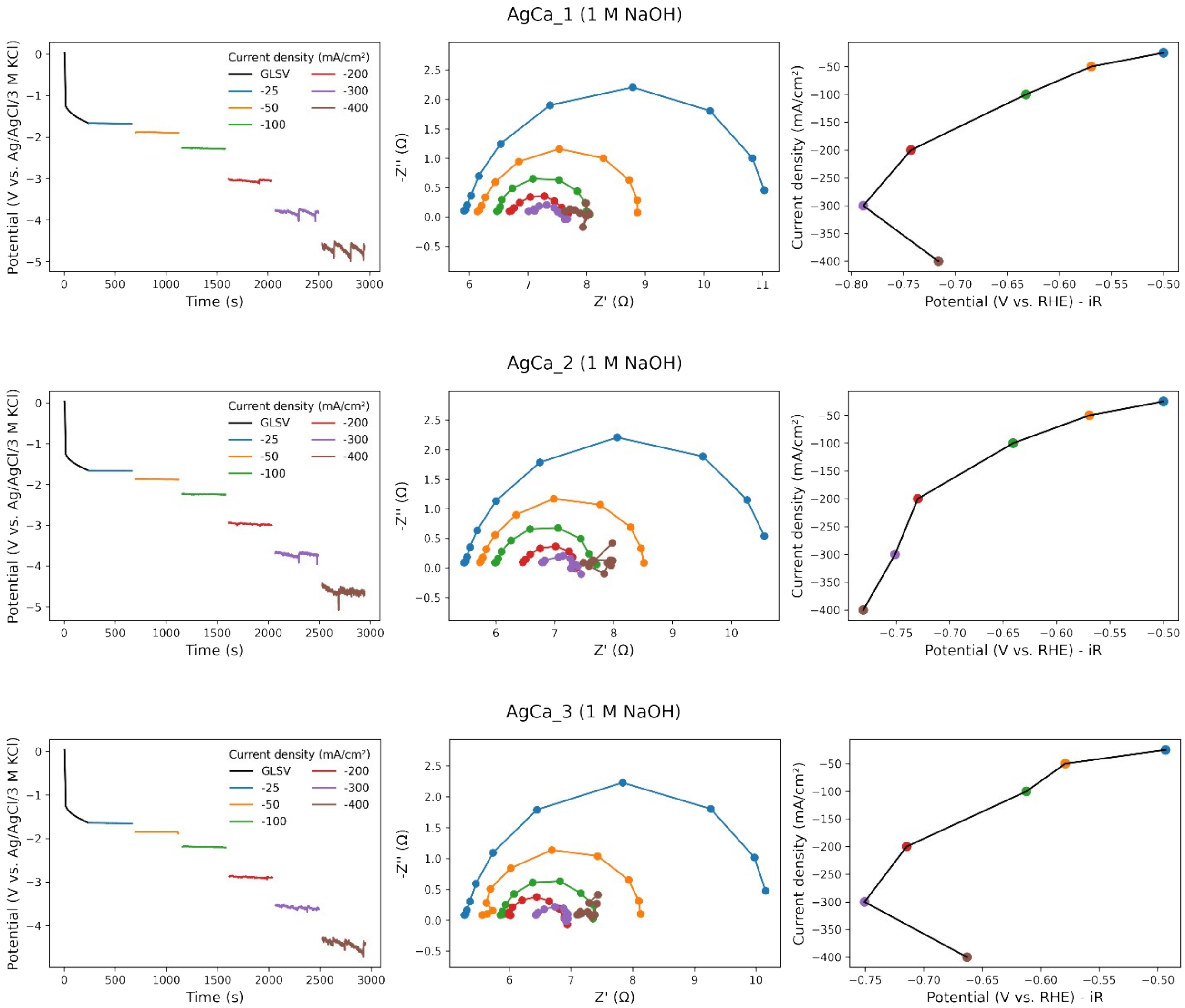


**Figure S14.** Potential profile for triplicate measurements of AgCa. Left: Uncompensated potential vs. Ag/AgCl/3 M KCl as a function of time. Middle: Galvanostatic electrochemical impedance spectroscopy (GEIS) measured at the end of each current step. Right: Current density (-25 to -400 mA $cm^{-2}$) and iR-corrected potential vs. RHE. Note that the value at -400 mA $cm^{-2}$ may be unreliable due to vigorous bubble formation interfering with accurate resistance measurement. Average potentials are shown in Figure 2d.

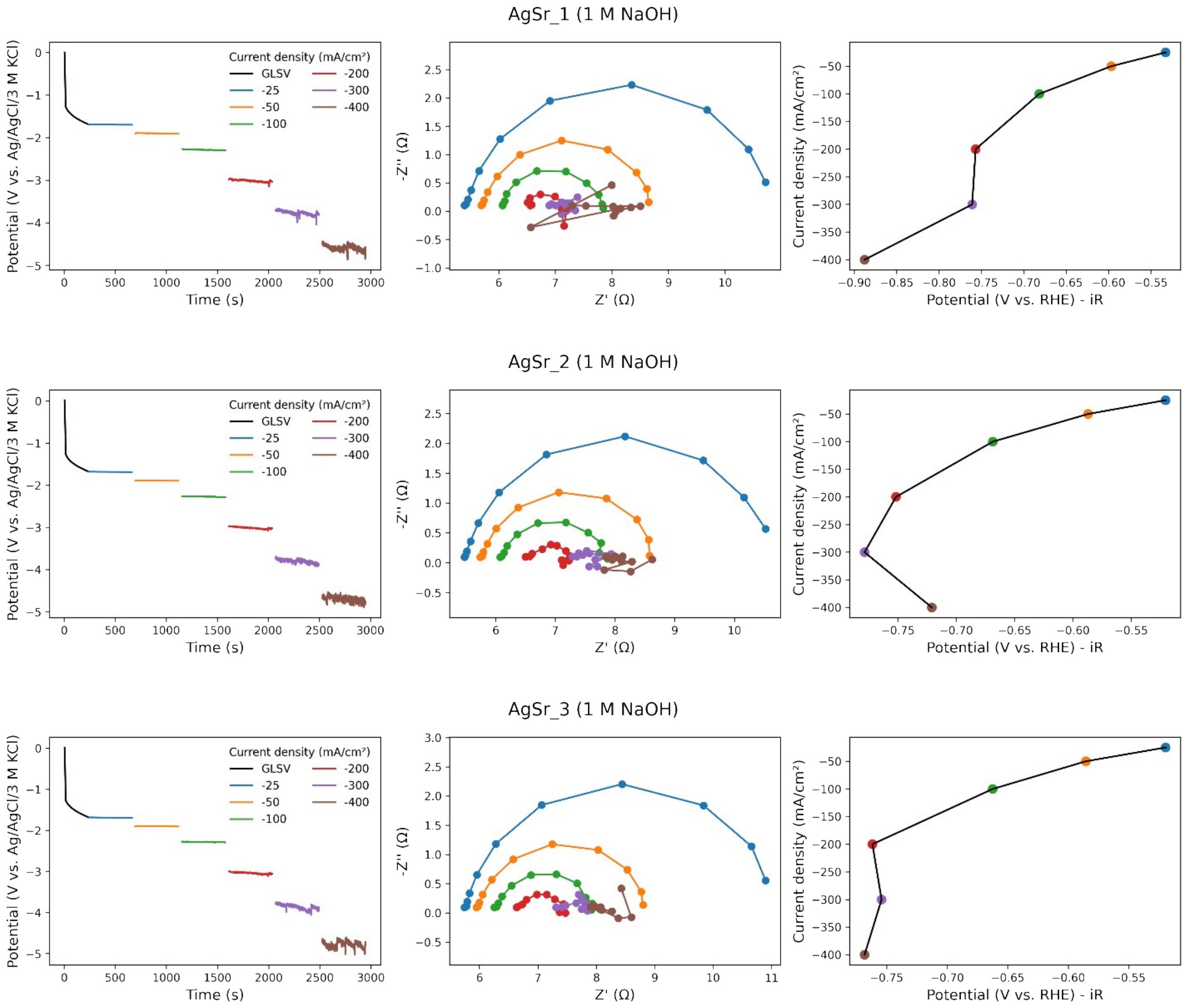


**Figure S15.** Potential profile for triplicate measurements of AgSr. Left: Uncompensated potential vs. Ag/AgCl/3 M KCl as a function of time. Middle: Galvanostatic electrochemical impedance spectroscopy (GEIS) measured at the end of each current step. Right: Current density (-25 to -400 mA cm$^{-2}$) and iR-corrected potential vs. RHE. Note that the value at -400 mA cm$^{-2}$ may be unreliable due to vigorous bubble formation interfering with accurate resistance measurement. Average potentials are shown in Figure 2d.

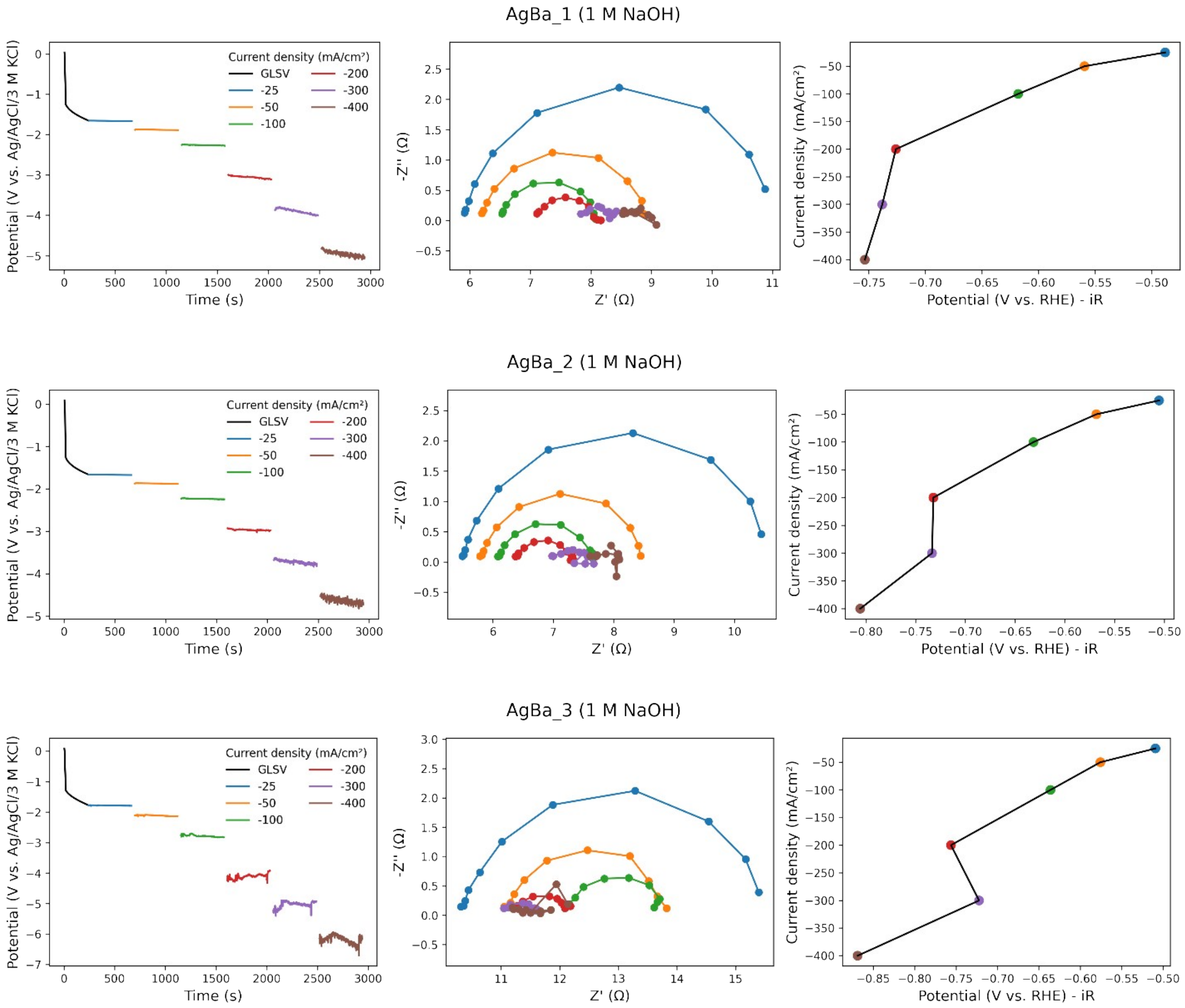


**Figure S16.** Potential profile for triplicate measurements of AgBa. Left: Uncompensated potential vs. Ag/AgCl/3 M KCl as a function of time. Middle: Galvanostatic electrochemical impedance spectroscopy (GEIS) measured at the end of each current step. Right: Current density (-25 to -400 mA $cm^{-2}$) and iR-corrected potential vs. RHE. Note that the value at -400 mA $cm^{-2}$ may be unreliable due to vigorous bubble formation interfering with accurate resistance measurement. Average potentials are shown in Figure 2d.

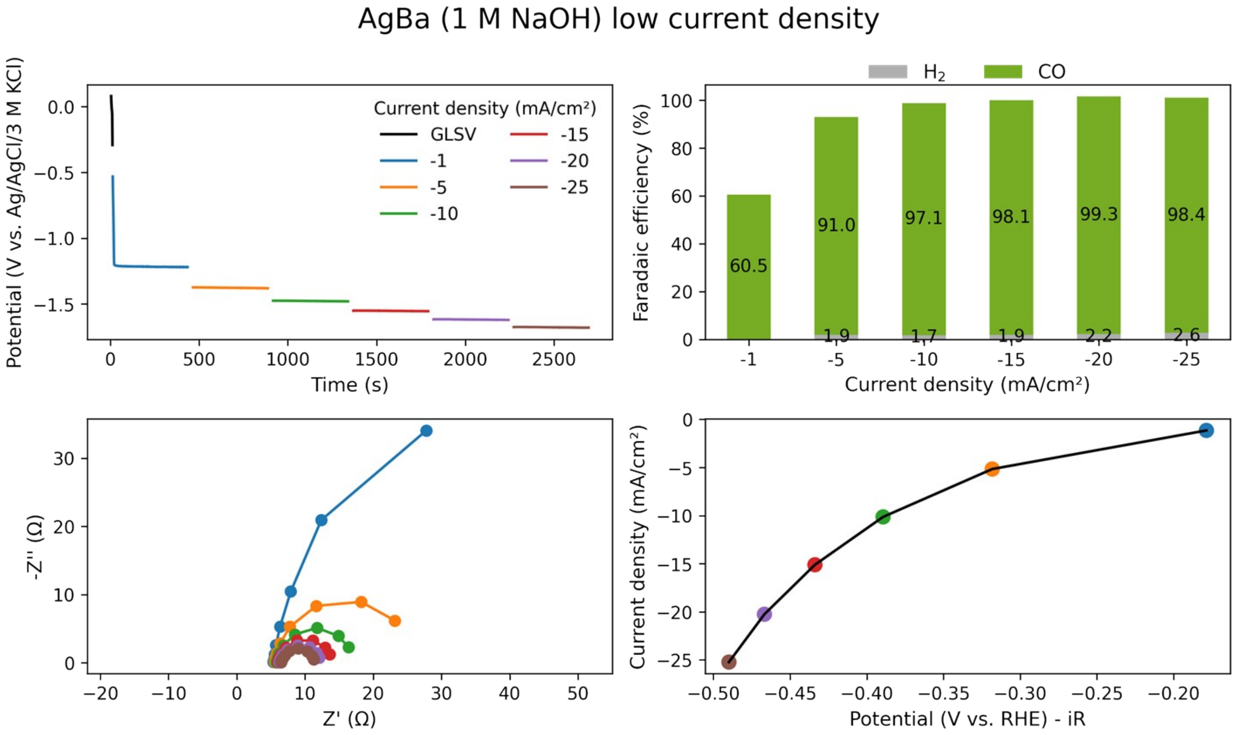


**Figure S17.** Electrochemical performance of AgBa for $CO_2$RR in 1 M NaOH at low current densities (-1 to -25 mA cm$^{-2}$), which corresponds to approximately -0.2 V to -0.5 V vs. RHE after iR correction. In this potential window, CO is essentially the only product, reaching nearly 100% Faradaic efficiency. At -1 mA cm$^{-2}$, the measured FE is only about 60% due to the low CO concentration detected by GC (127 ppm, compared to the typical thousands of ppm), potentially leading to a large measurement error. Nevertheless, $H_2$ remains barely detectable across this potential range, confirming that the observed activity corresponds to $CO_2$RR rather than HER at low current density.

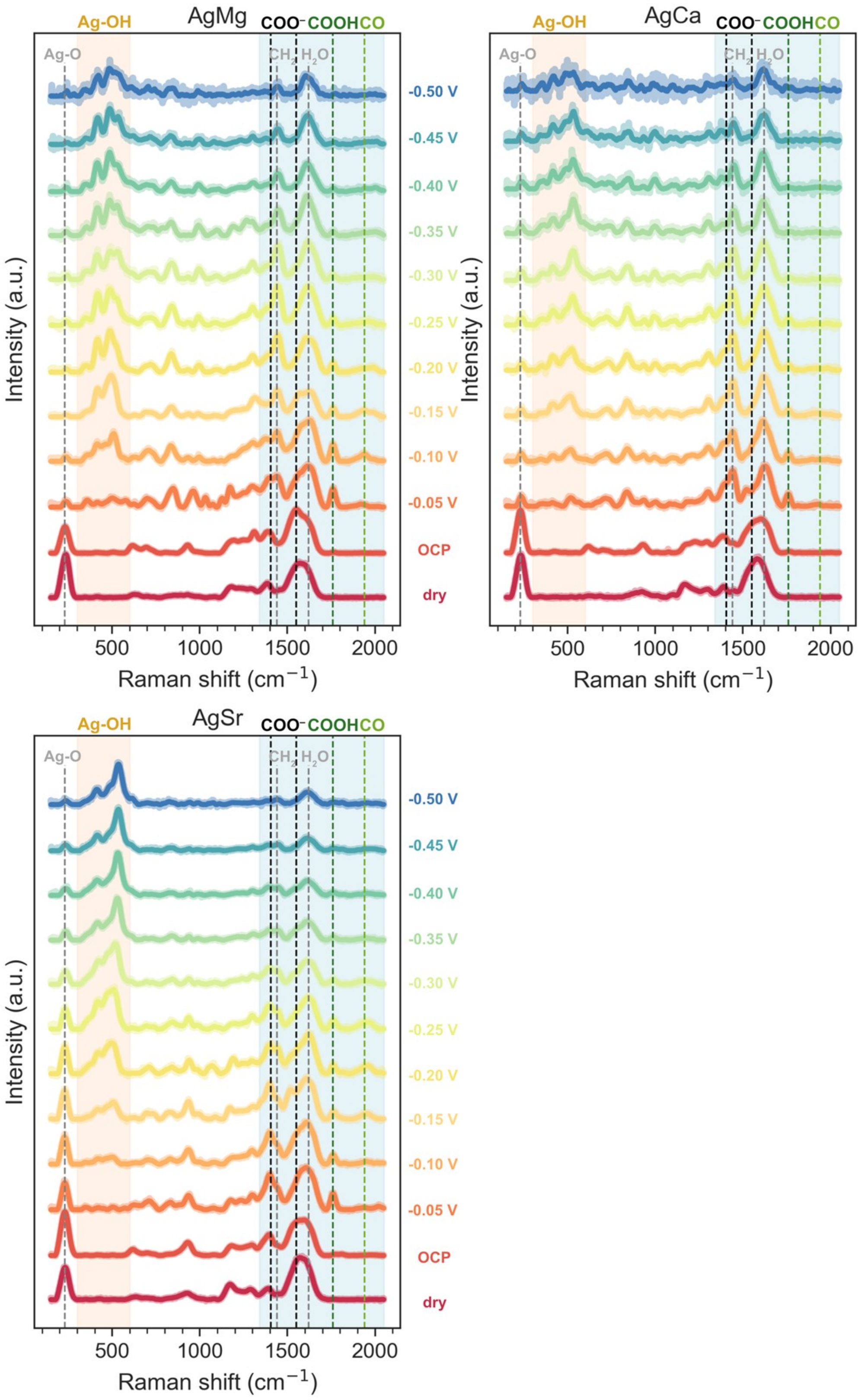


**Figure S18.** Operando Raman spectra for AgMg, AgCa, AgSr in the potential range from -0.05 V to -0.5 V vs. RHE in 0.1 M NaOH + 0.5 M $Na_2SO_4$ (pH 13). Comparable figures for Ag and AgBa shown in Figure 3.

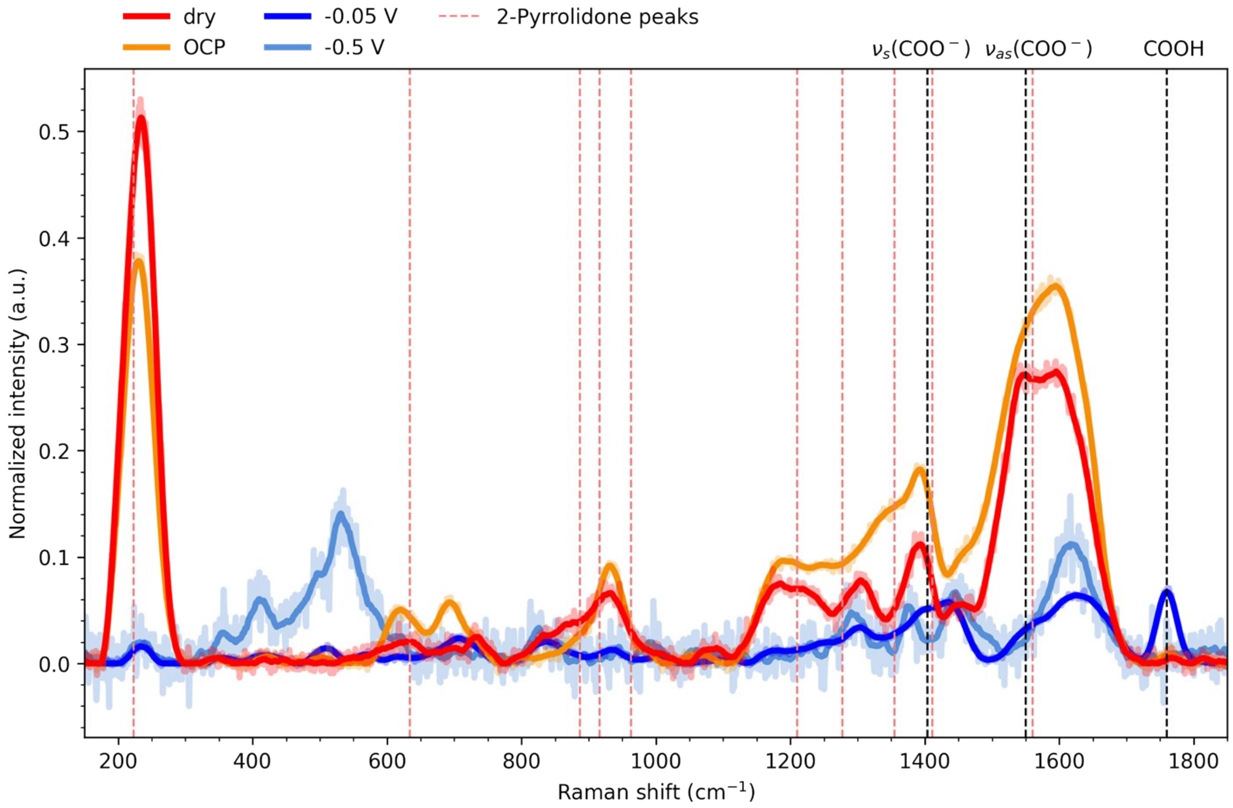


**Figure S19.** Raman spectra of AgBa recorded before applying potential (dry and OCP) and after applying cathodic potential (-0.05 V and -0.5 V). Red vertical lines indicate reference Raman bands from the adsorption of 2-pyrrolidone (the monomer of PVP) on Ag colloids, as reported by Mdluli et al.[4] The spectra of AgBa at dry and OCP conditions show peaks that closely match those of 2-pyrrolidone, suggesting that these peaks originate from the adsorption of PVP. However, as soon as a cathodic potential is applied (-0.05 V), these peaks are strongly diminished, consistent with PVP desorption and enabling the observation of Raman-active intermediates relevant to $CO_2$RR ($COO^-$, COOH, CO) and HER.

**Table S4.** Raman peak assignments for 2-pyrrolidone adsorbed on Ag colloids, extracted from Mdluli et al.[4]

| Raman shift ($cm^{-1}$) | Assignment |
|---|---|
| 223 | Ag–O stretching |
| 634 | CCC in plane bend |
| 887 | Ring breathing, in plane |
| 916 | CH out plane bend |
| 963 | Breathing |
| 1210 | β(C-C=O) |
| 1277 | CH in plane band |
| 1355 | Ring breathing, antisymmetric-scissor |
| 1411 | (CC, CN) |
| 1560 | (CC) |

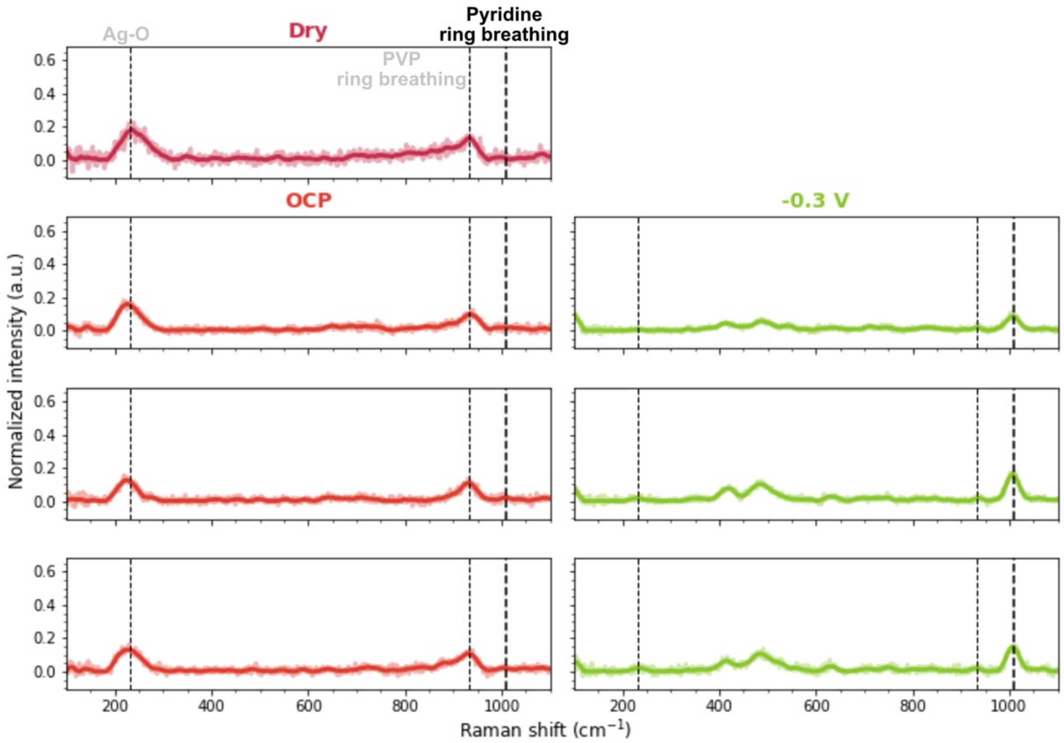


**Figure S20.** Raman spectra of Ag measured before electrolyte addition (dry) and in 0.1 M NaOH + 0.5 M $Na_2SO_4$ + 1 mM pyridine (without $CO_2$ supply) at OCP and -0.3 V vs. RHE. Spectra were recorded at three different positions of the electrode for OCP and -0.3 V, and the average intensity of the pyridine ring-breathing mode at 1008 cm$^{-1}$ is plotted in Figure S22.

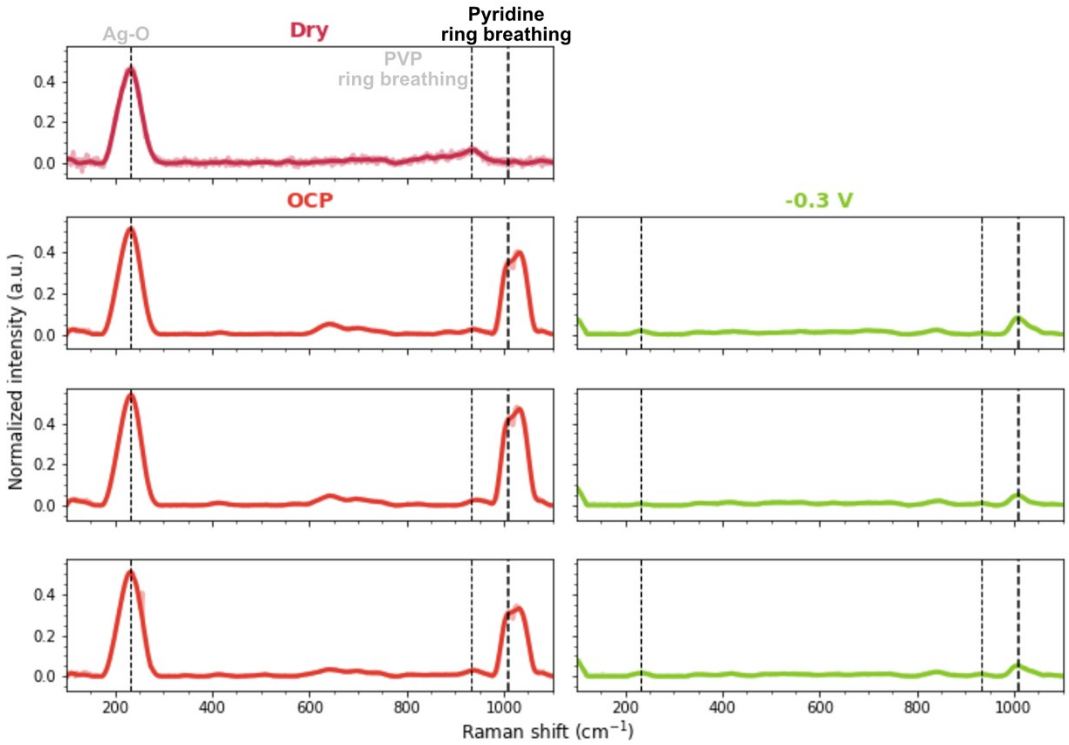


**Figure S21.** Raman spectra of AgBa measured before electrolyte addition (dry) and in 0.1 M NaOH + 0.5 M $Na_2SO_4$ + 1 mM pyridine (without $CO_2$ supply) at OCP and -0.3 V vs. RHE. Spectra were recorded at three different positions of the electrode for OCP and -0.3 V, and the average intensity of the pyridine ring-breathing mode at 1008 cm$^{-1}$ is plotted in Figure S22.

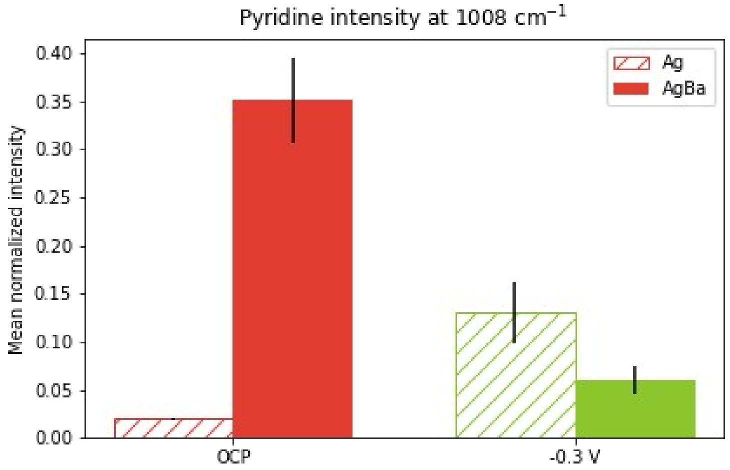


**Figure S22.** Average intensity of the pyridine ring-breathing mode at 1008 cm$^{-1}$ for Ag (Figure S20) and AgBa (Figure S21) at OCP and -0.3 V vs. RHE. While AgBa shows higher intensity than Ag at OCP, at -0.3 V the pyridine intensities are comparable. This indicates that physical SERS effects are similar for both samples under cathodic conditions, suggesting that the enhanced signals of $CO_2$RR intermediates on modified Ag are likely due to chemical stabilization.

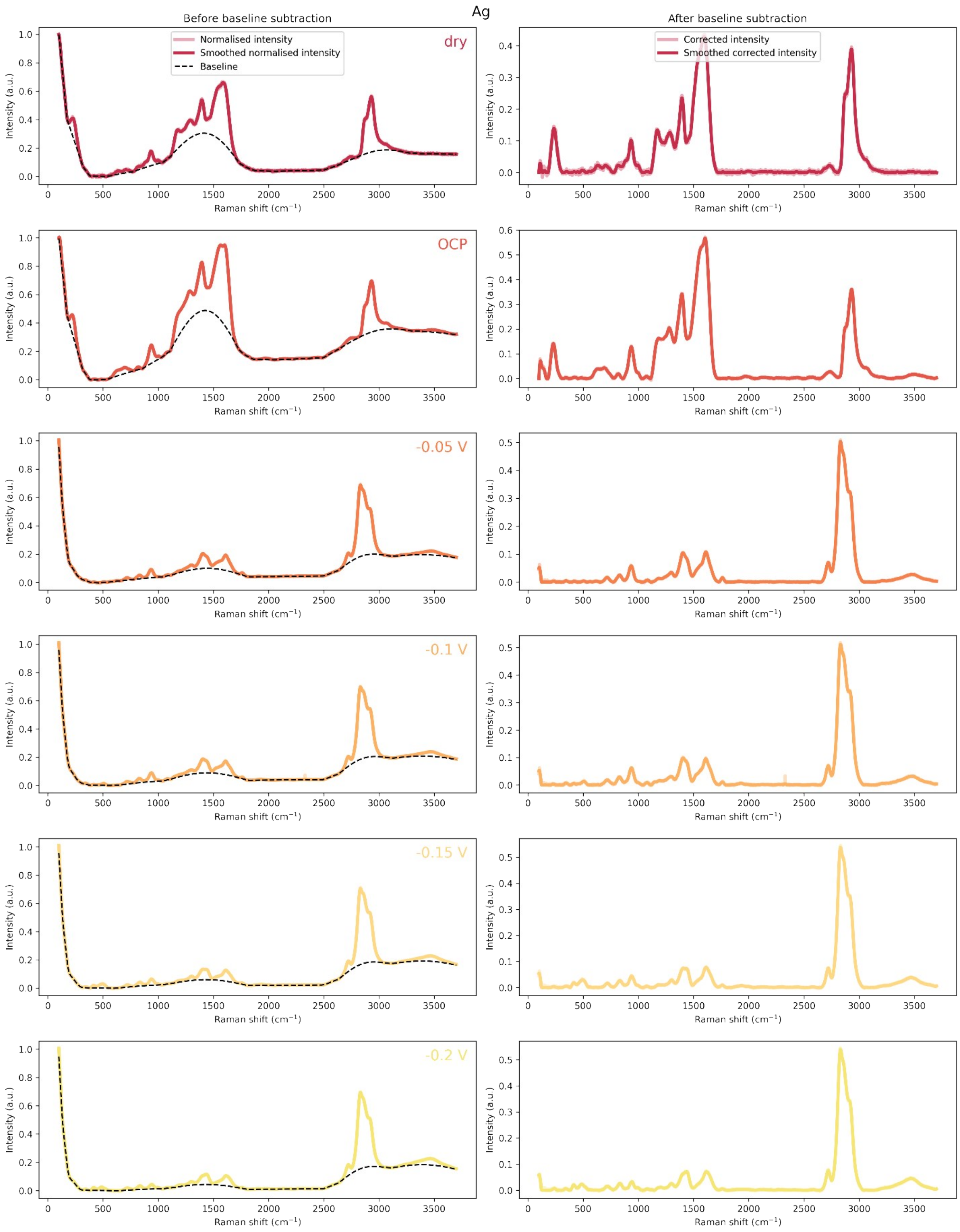

Ag
Before baseline subtraction
After baseline subtraction
Normalised intensity
Smoothed normalised intensity
Baseline
Corrected intensity
Smoothed corrected intensity
dry
OCP
-0.05 V
-0.1 V
-0.15 V
-0.2 V
Intensity (a.u.)
Raman shift (cm$^{-1}$)

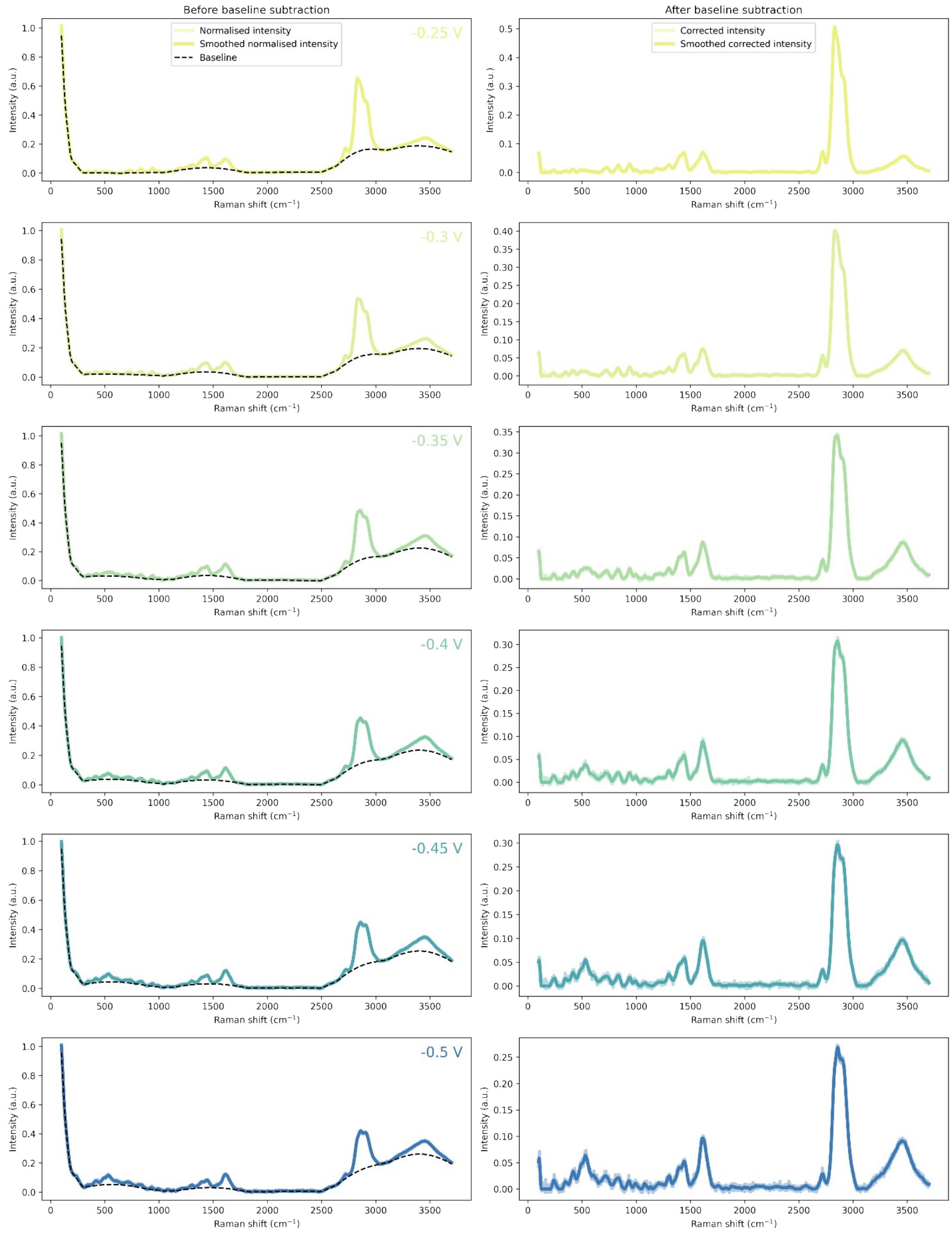


**Figure S23.** Normalized Raman spectra of Ag at various applied potentials before and after baseline subtraction using the SNIP algorithm.

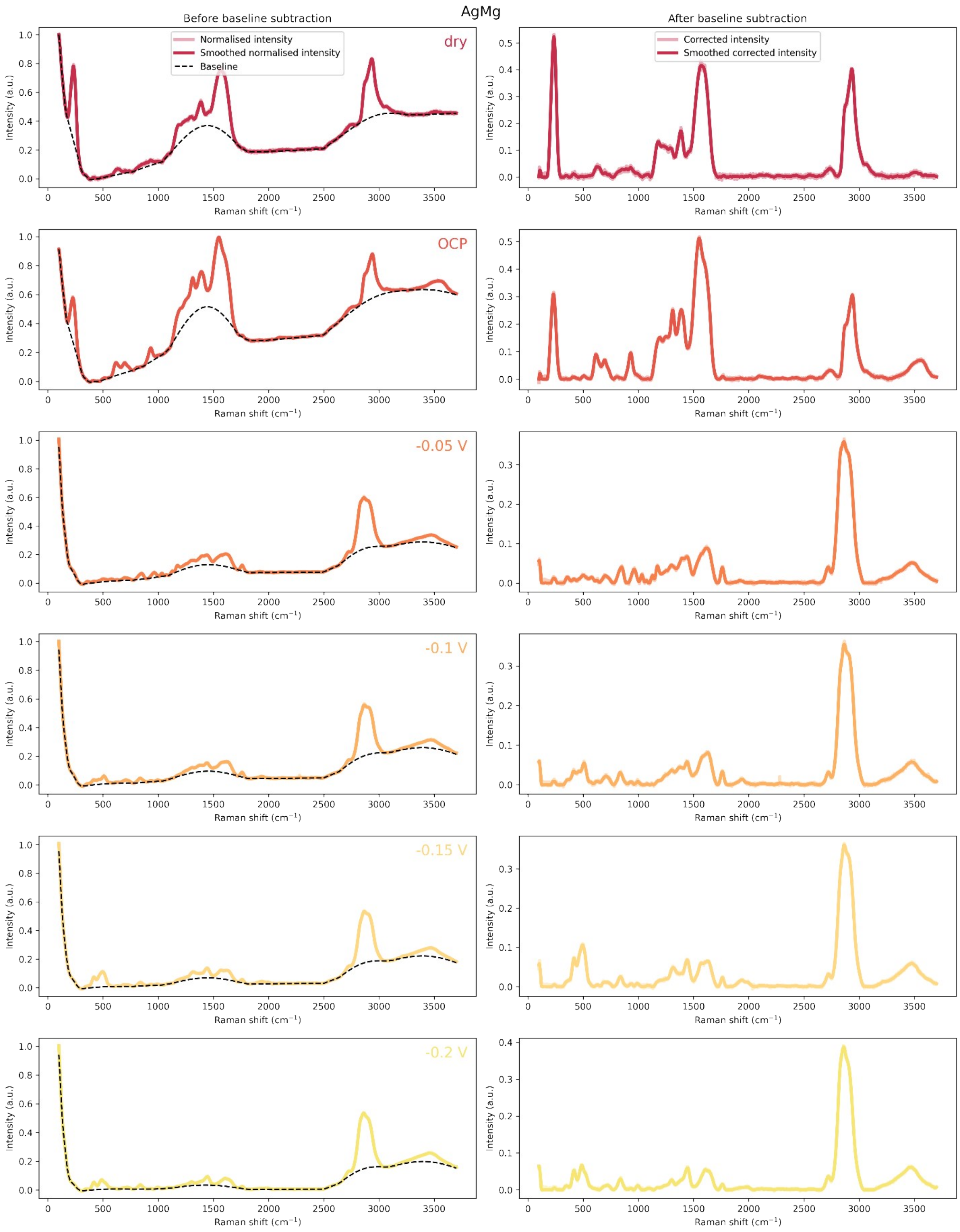

AgMg
Before baseline subtraction
After baseline subtraction
Normalised intensity
Smoothed normalised intensity
Baseline
Corrected intensity
Smoothed corrected intensity
dry
OCP
-0.05 V
-0.1 V
-0.15 V
-0.2 V
Intensity (a.u.)
Raman shift (cm$^{-1}$)

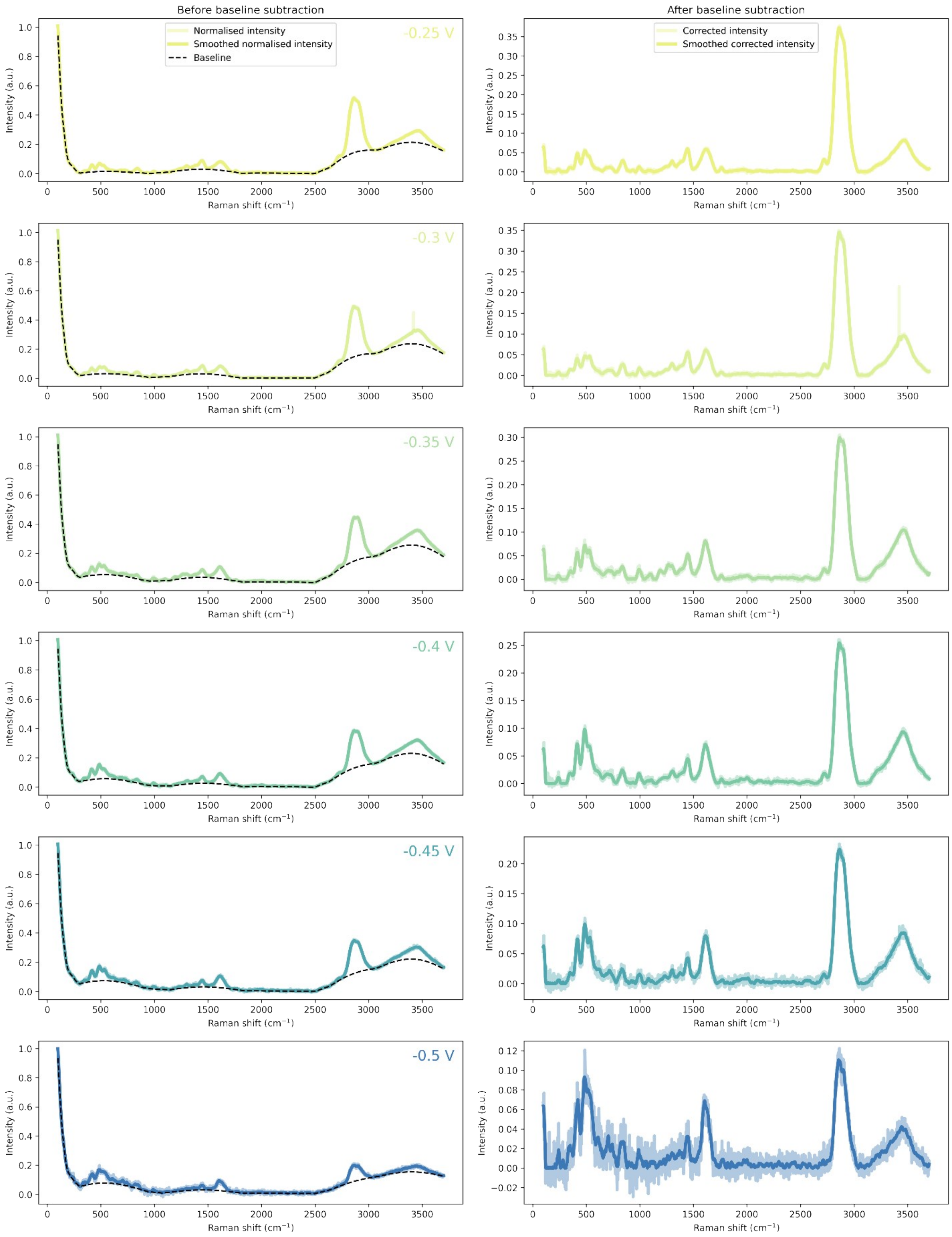


**Figure S24.** Normalized Raman spectra of AgMg at various applied potentials before and after baseline subtraction using the SNIP algorithm.

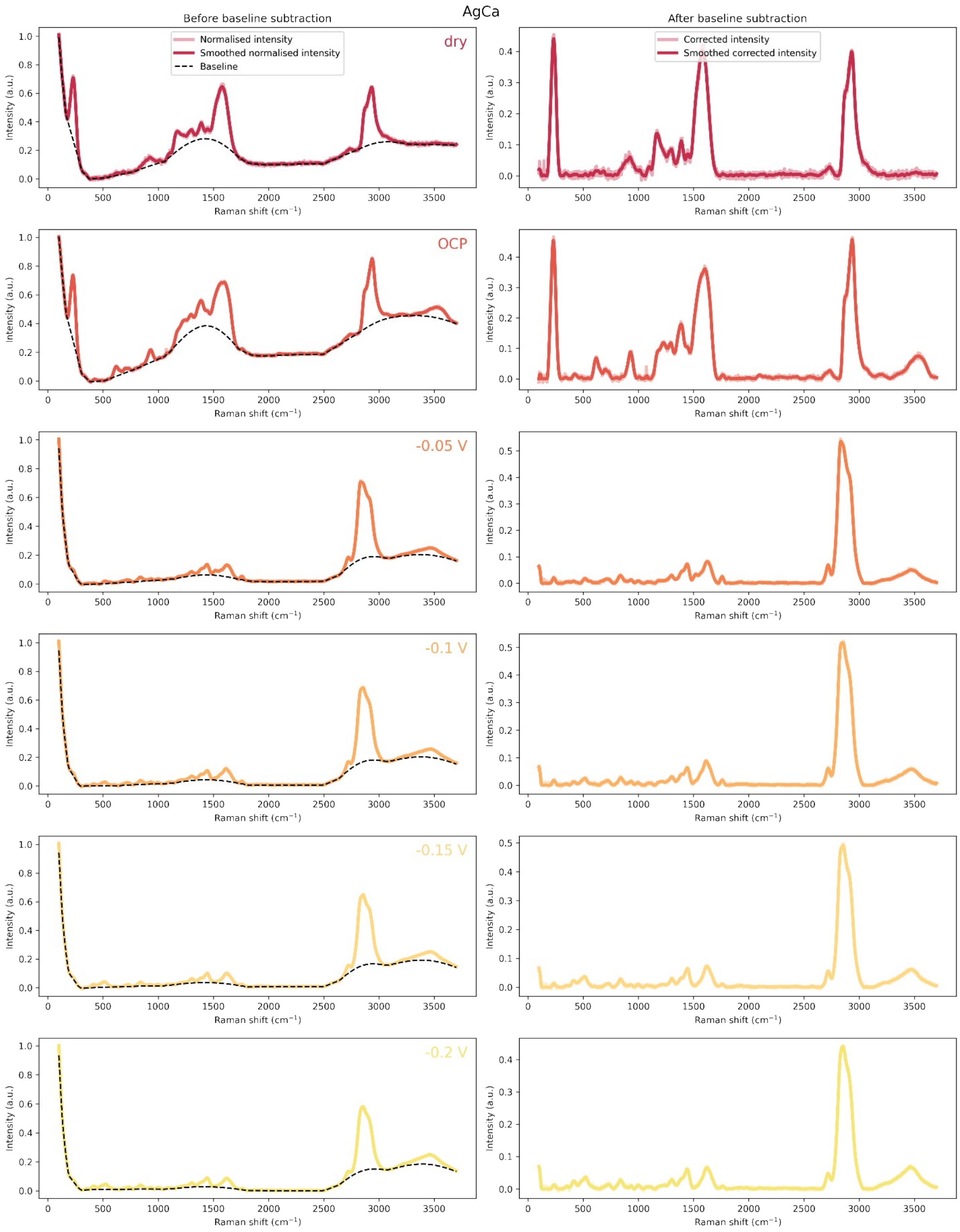
AgCa
Before baseline subtraction
After baseline subtraction
Normalised intensity
Smoothed normalised intensity
Baseline
Corrected intensity
Smoothed corrected intensity
dry
OCP
-0.05 V
-0.1 V
-0.15 V
-0.2 V
Intensity (a.u.)
Raman shift (cm⁻¹)

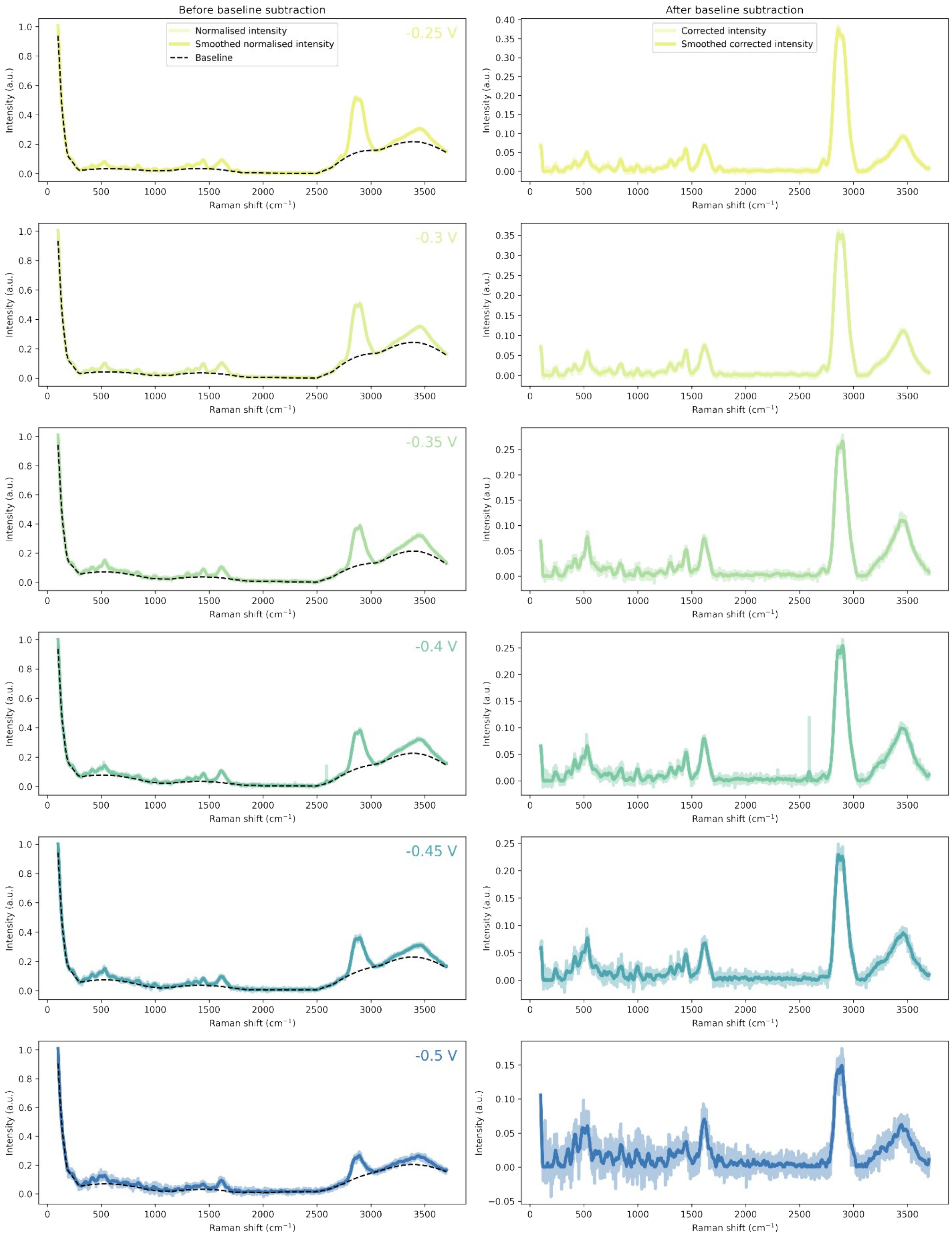


**Figure S25.** Normalized Raman spectra of AgCa at various applied potentials before and after baseline subtraction using the SNIP algorithm.

AgSr

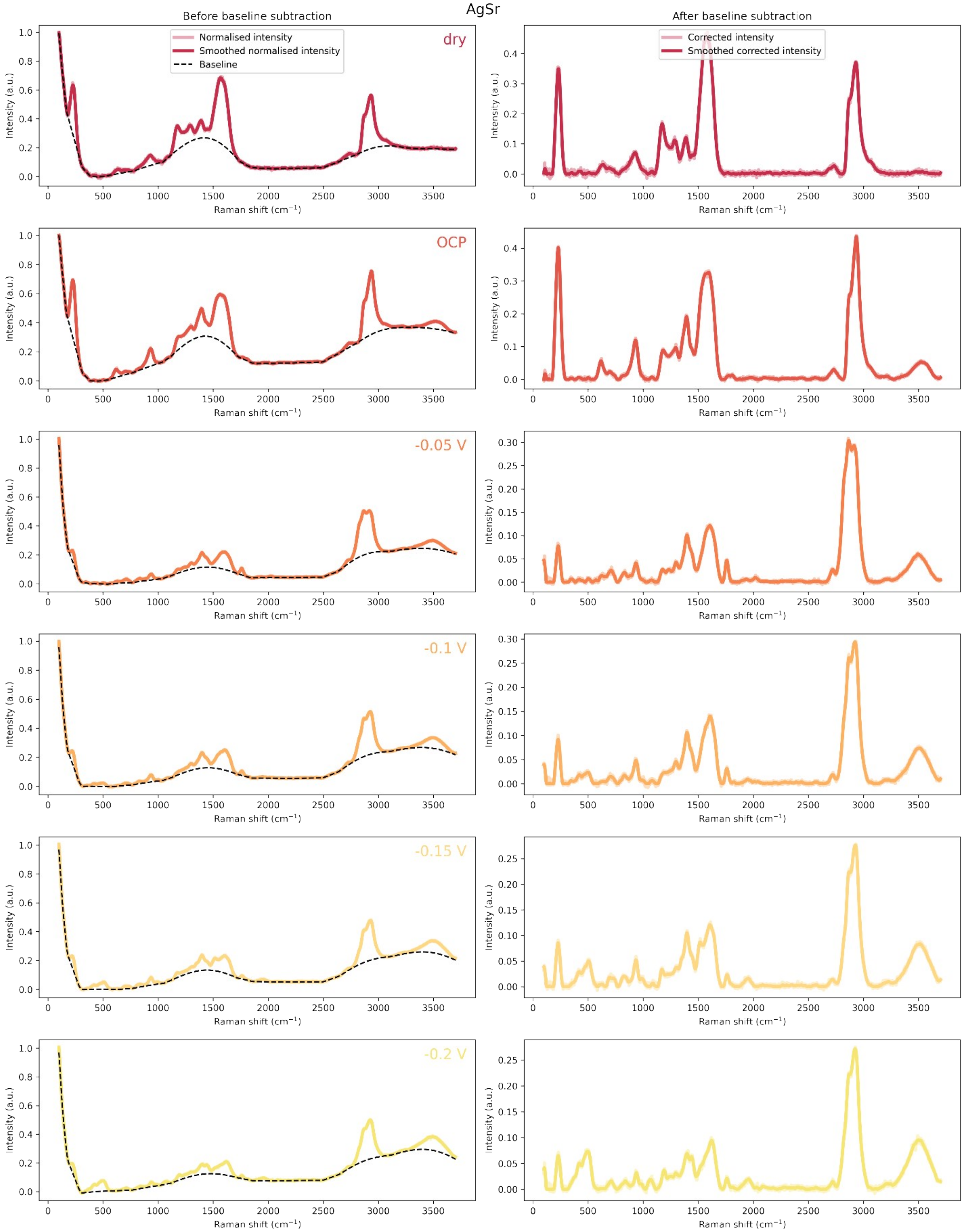

Before baseline subtraction
After baseline subtraction
Normalised intensity
Smoothed normalised intensity
Baseline
Corrected intensity
Smoothed corrected intensity
dry
OCP
-0.05 V
-0.1 V
-0.15 V
-0.2 V
Intensity (a.u.)
Raman shift (cm$^{-1}$)

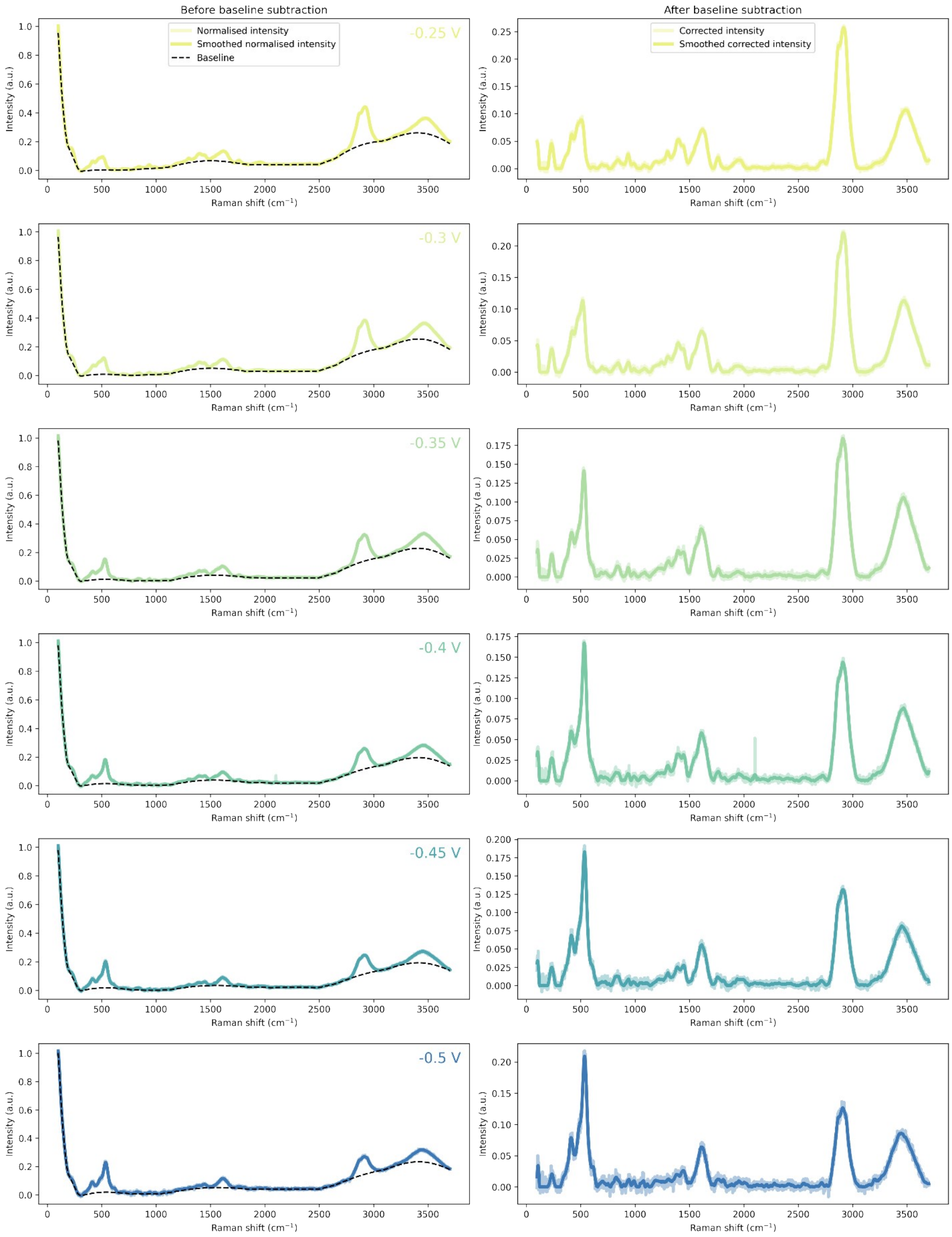


**Figure S26.** Normalized Raman spectra of AgSr at various applied potentials before and after baseline subtraction using the SNIP algorithm.

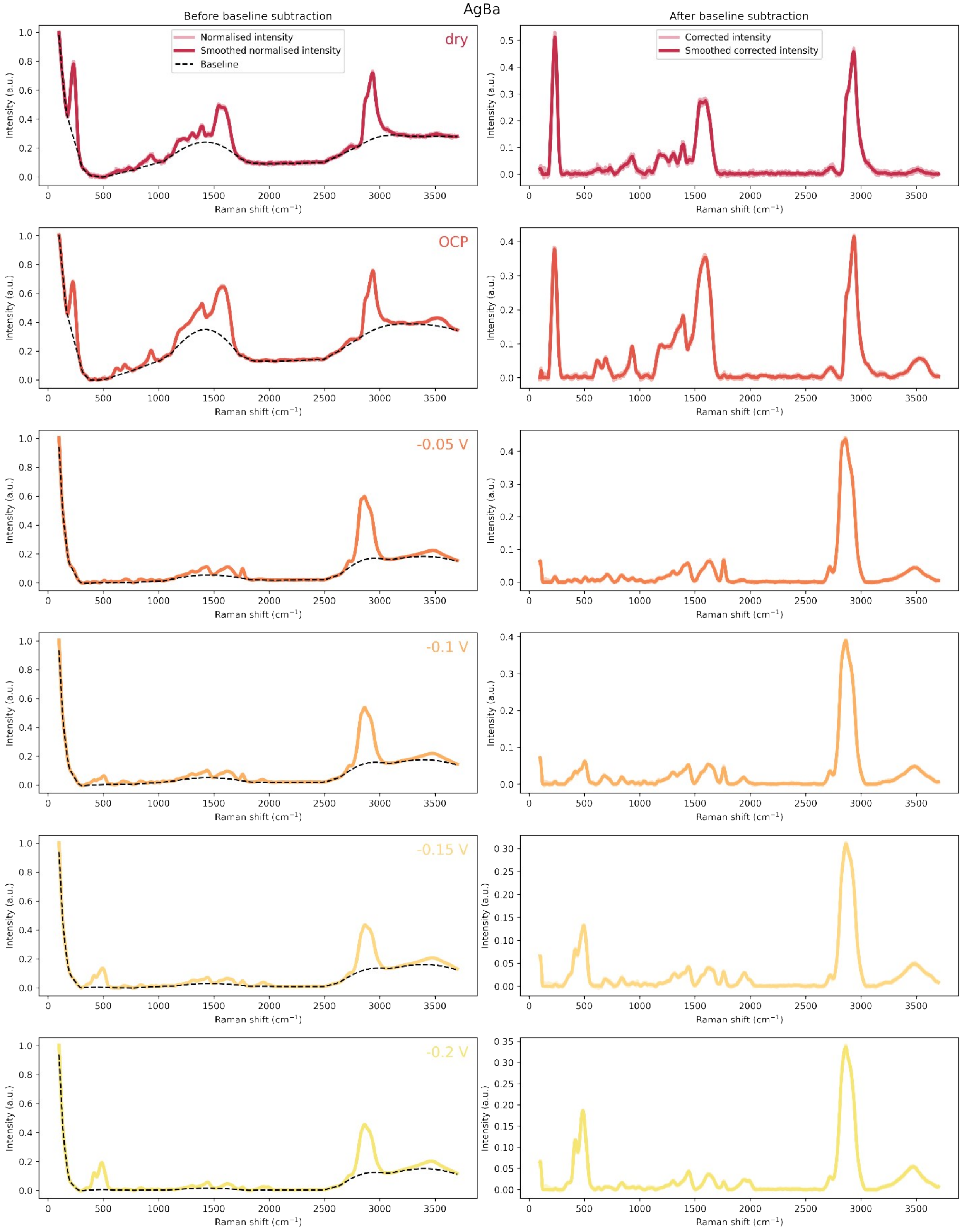
AgBa
Before baseline subtraction
After baseline subtraction
Normalised intensity
Smoothed normalised intensity
Baseline
Corrected intensity
Smoothed corrected intensity
dry
OCP
-0.05 V
-0.1 V
-0.15 V
-0.2 V
Intensity (a.u.)
Raman shift (cm$^{-1}$)

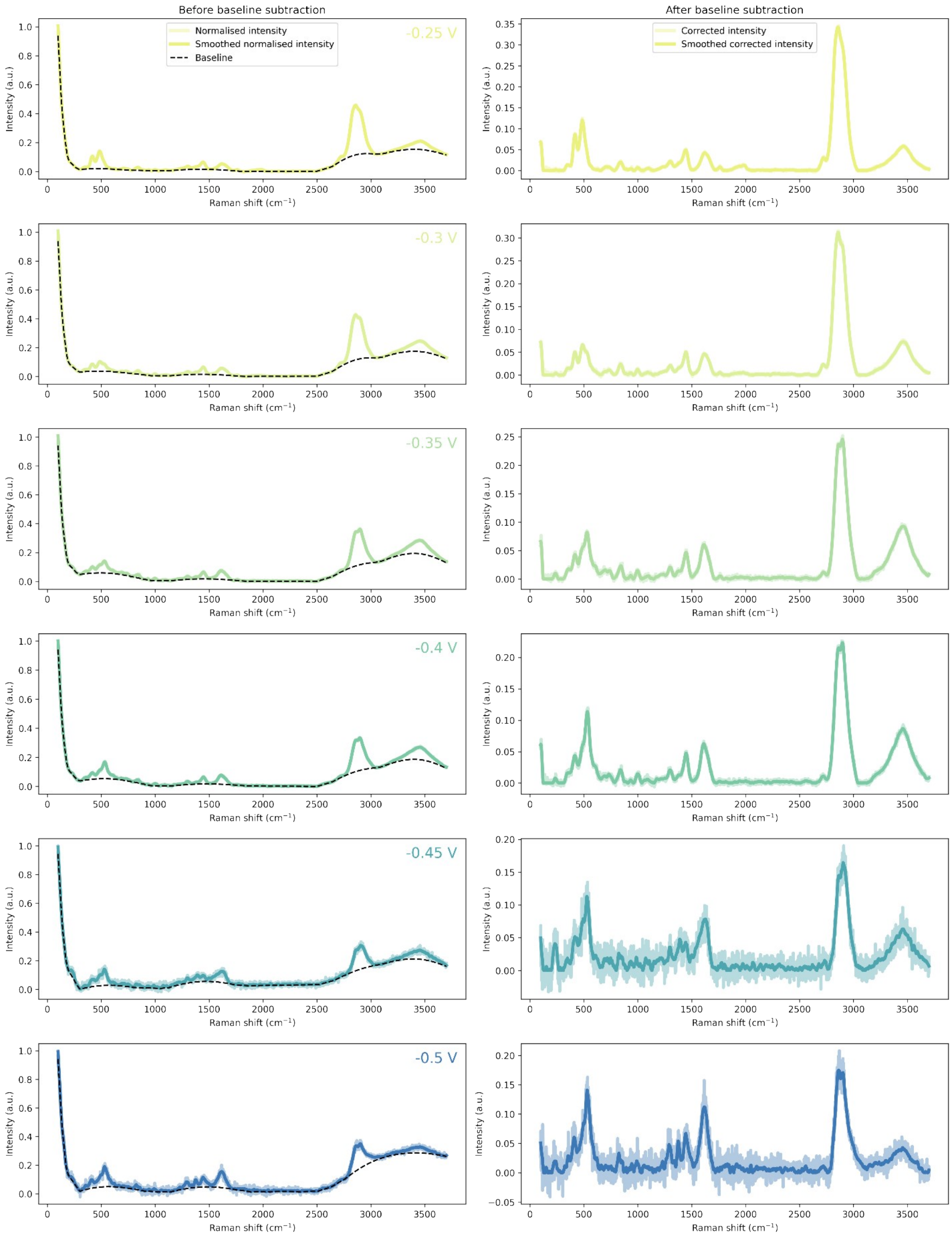


**Figure S27.** Normalized Raman spectra of AgBa at various applied potentials before and after baseline subtraction using the SNIP algorithm.

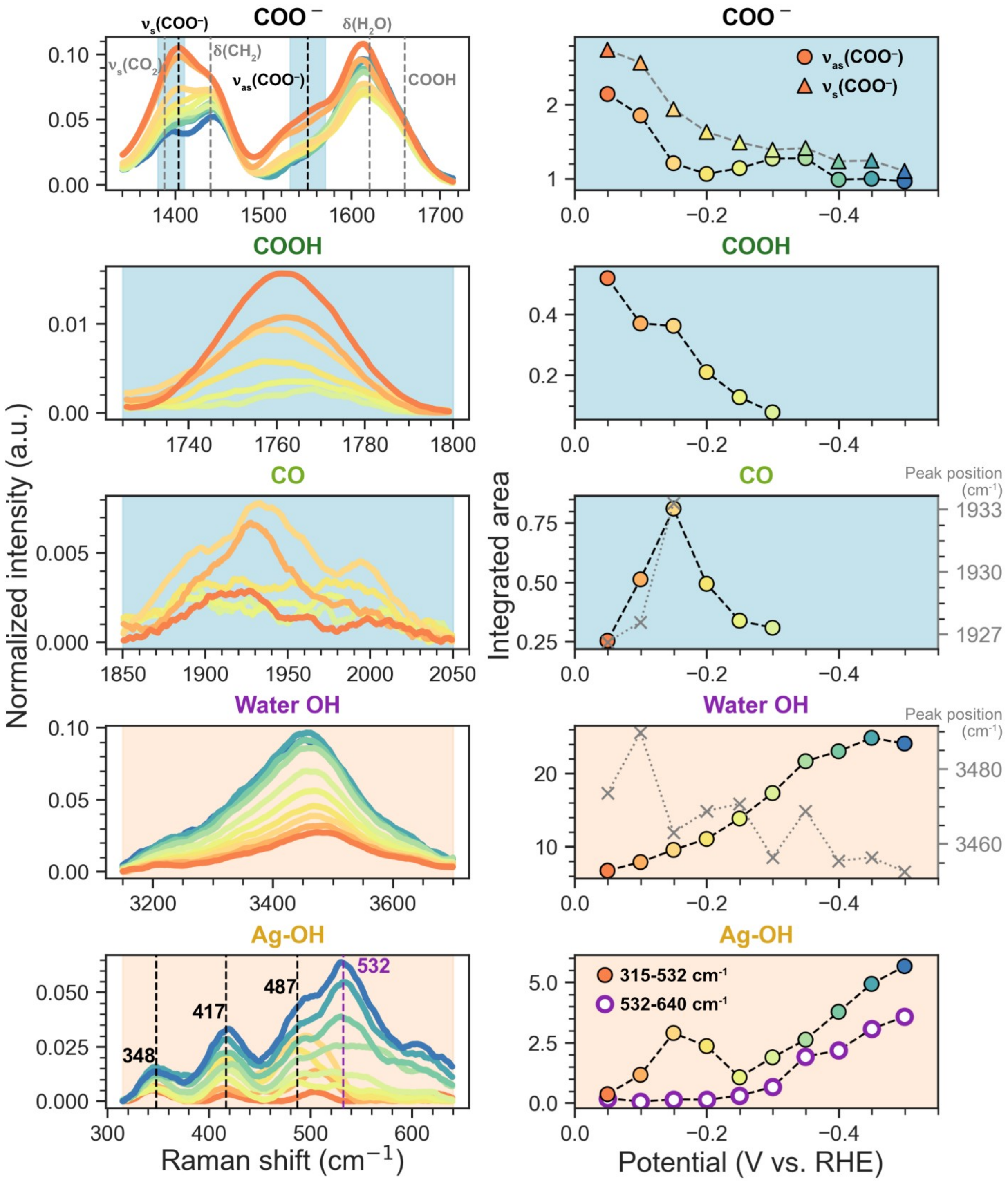


**Figure S28.** Normalized Raman intensities of Ag measured from −0.05 to −0.5 V vs. RHE for $*COO^-$, *COOH, *CO, water OH, and Ag–OH, with their corresponding integrated areas. The shaded backgrounds indicate the range for integration. For clarity, only smoothed signals are plotted here. Comparable figure for AgBa plotted in Figure 4.

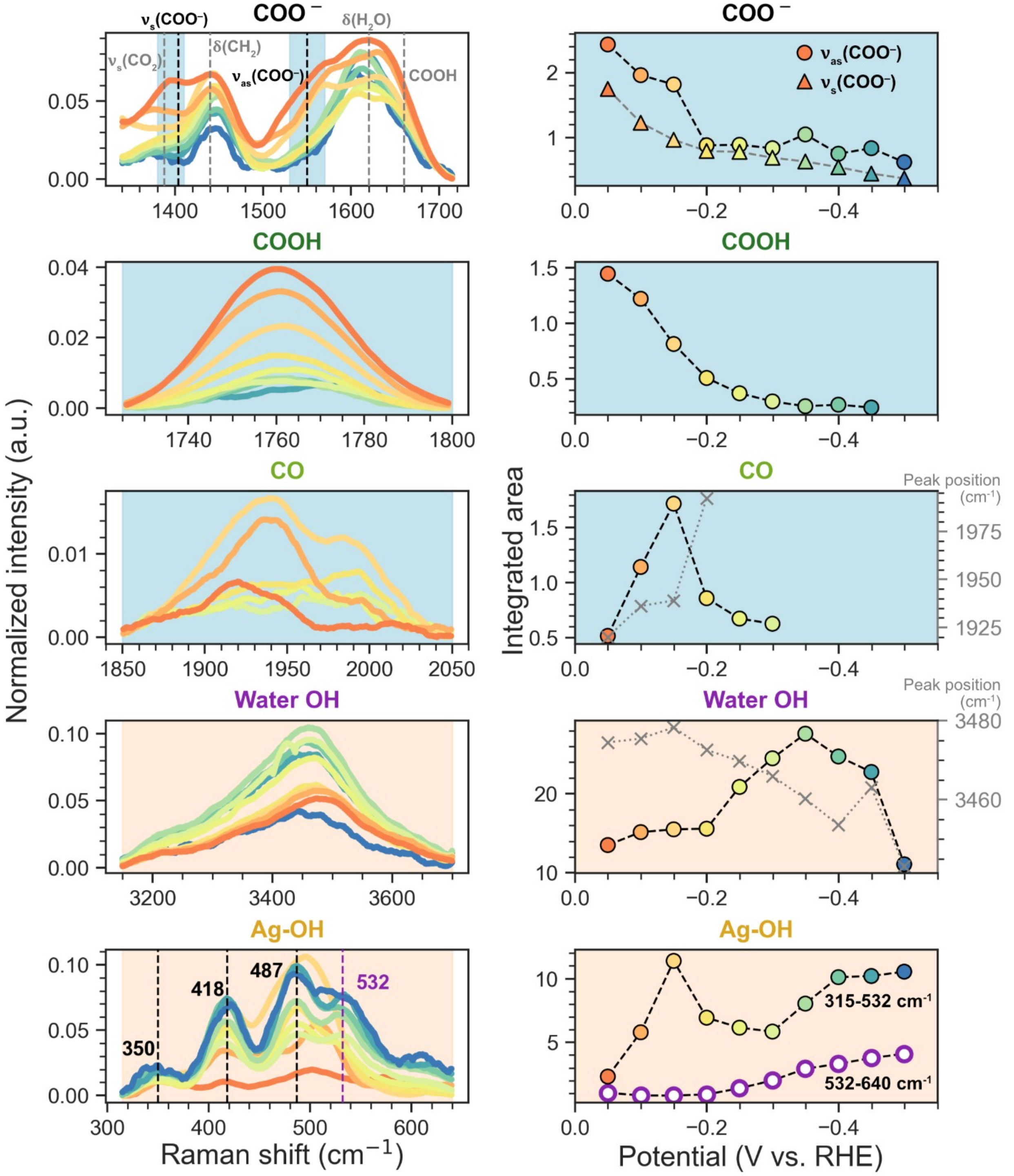


**Figure S29.** Normalized Raman intensities of AgMg measured from −0.05 to −0.5 V vs. RHE for $*COO^-$, *COOH, *CO, water OH, and Ag–OH, with their corresponding integrated areas. The shaded backgrounds indicate the range for integration. For clarity, only smoothed signals are plotted here. Comparable figure for AgBa plotted in Figure 4.

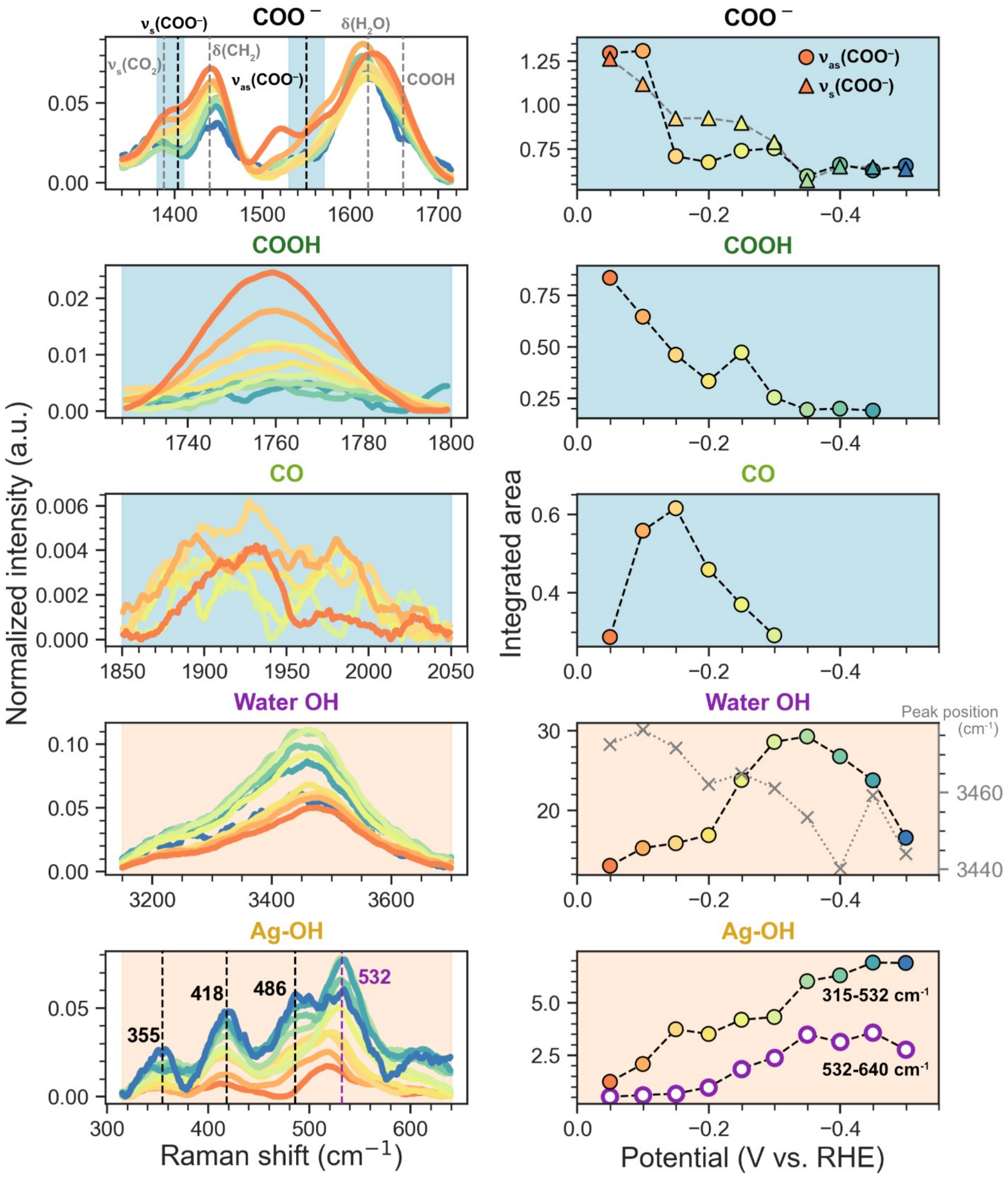


**Figure S30.** Normalized Raman intensities of AgCa measured in the potential range from −0.05 to −0.5 V vs. RHE for *$COO^-$, *COOH, *CO, water OH, and Ag–OH, with their corresponding integrated areas. The shaded backgrounds indicate the range for integration. For clarity, only smoothed signals are plotted here. Comparable figure for AgBa plotted in Figure 4.

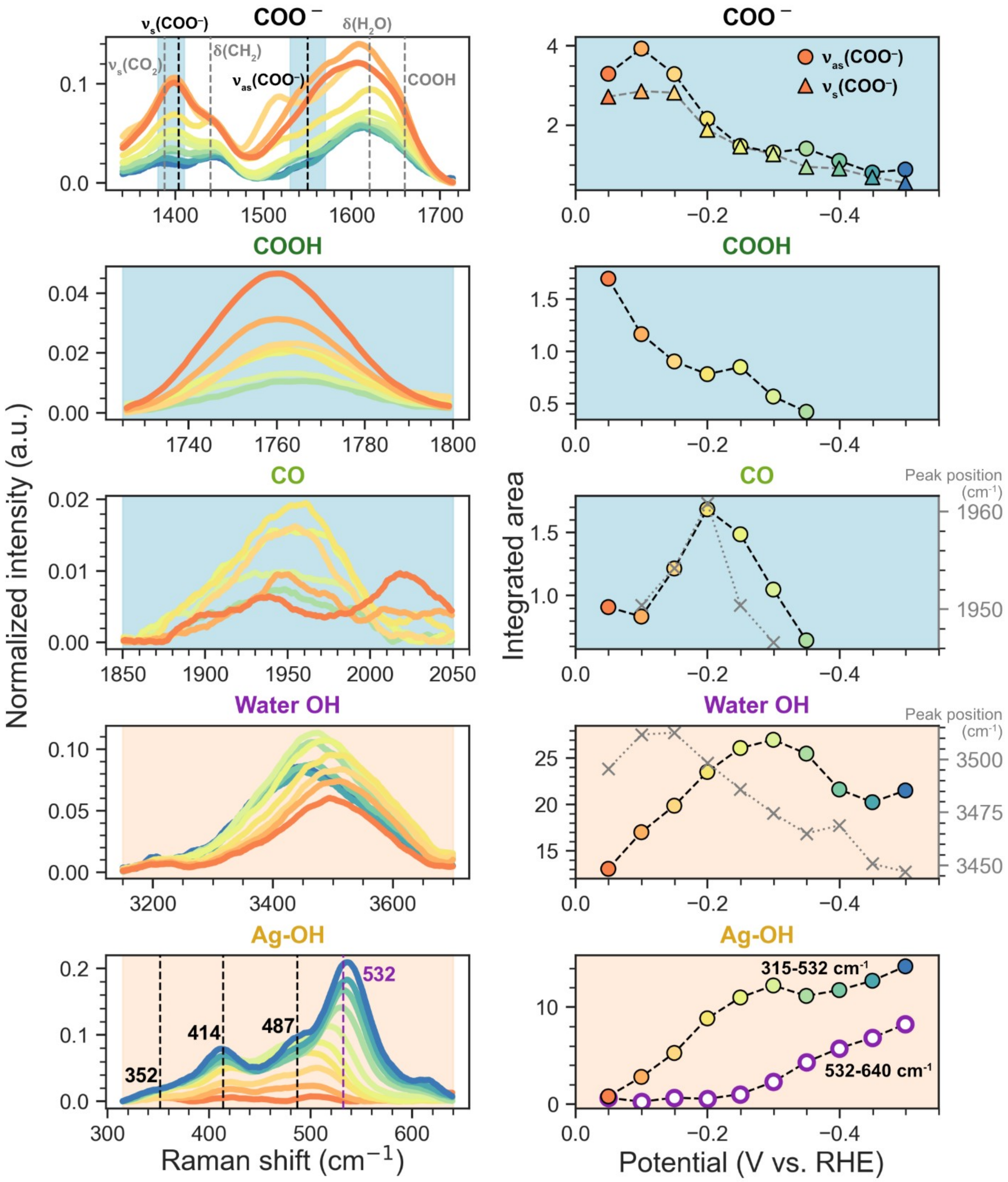


**Figure S31.** Normalized Raman intensities of AgSr measured in the potential range from −0.05 to −0.5 V vs. RHE for $*COO^-$, *COOH, *CO, water OH, and Ag–OH, with their corresponding integrated areas. The shaded backgrounds indicate the range for integration. For clarity, only smoothed signals are plotted here. Comparable figure for AgBa plotted in Figure 4.

**Table S5.** Comparison of COO⁻ stretching frequencies for COO⁻ intermediate and formate. While $\nu_s(COO^-)$ of formate generally appears at lower wavenumbers than the 1404 $cm^{-1}$ band assigned to the COO⁻ intermediate, the $\nu_{as}(COO^-)$ modes of both species fall in a similar range at about 1550 $cm^{-1}$. Nevertheless, given the small amount of formate produced, its contribution to the observed COO⁻ signals is expected to be minimal.

| | $\nu_s(COO^-)$ ($cm^{-1}$) | $\nu_{as}(COO^-)$ ($cm^{-1}$) | Ref. |
|---|---|---|---|
| **Stretches used to follow the COO⁻ intermediate** | 1404 | 1550 | |
| **Sodium formate (experiment)** | 1366 | 1567 | [5] |
| **Sodium formate (DFT)** | 1351 | 1590 | [6] |
| **Various formates** | 1311–1387 | 1550–1613 | [5] |

**Table S6.** Literature vibrational frequencies ($cm^{-1}$) of CO on Ag in different coordination environments.

| Method | Ag-CO | Atop | Bridge | Hollow | Facet | Ref. |
|---|---|---|---|---|---|---|
| DFT | 203 | 2050 | 1935 | 1895 | (111) | [7] |
| DFT | / | 2122 | 2002 | 1957 (fcc), 1961 (hcp) | (111) | [8] |
| DFT | 173 | 2123 | / | / | (100) | [9] |
| DFT | 219 | 2123 | / | / | (111) | [10] |
| Experiment | 160 | 2113 | / | / | Ag colloids | [11] |

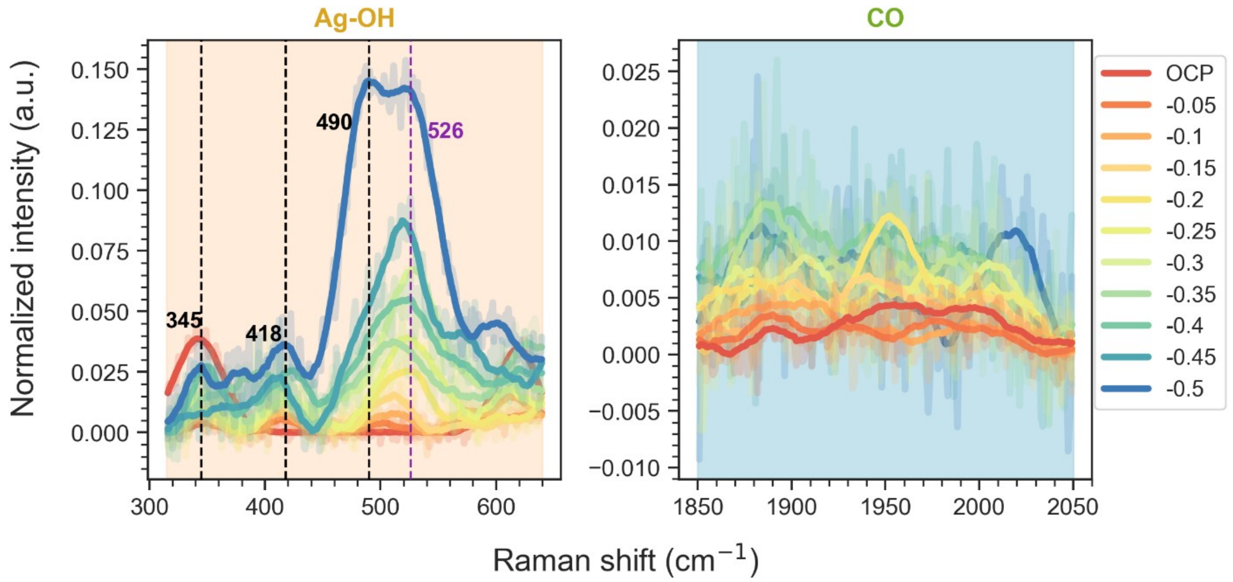


**Figure S32.** Normalized Raman intensities of AgBa in the Ag–OH and CO regions, measured in $N_2$-purged electrolyte (0.1 M NaOH + 0.5 M $Na_2SO_4$) at different applied potentials without $CO_2$ supply.

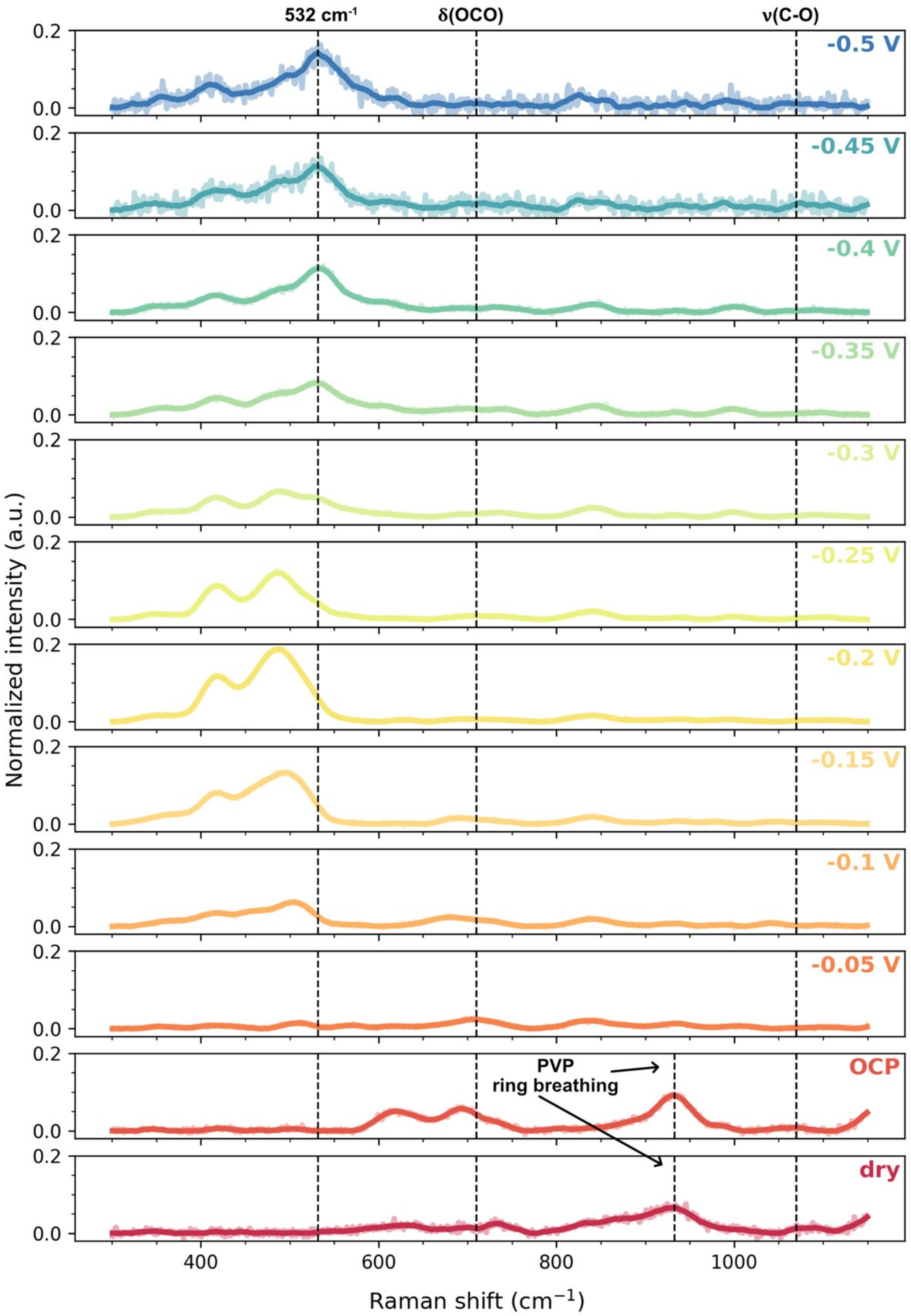


**Figure S33.** Potential-dependent Raman spectra of AgBa from -0.05 V to -0.5 V, plotted separately for clarity to highlight the emergence of the 532 $cm^{-1}$ feature (same dataset as Fig. 4e). No distinct signals are observed for the carbonate δ(O–C–O) bending mode (~700 $cm^{-1}$) and ν(C–O) stretch (1070–1080 $cm^{-1}$) throughout the cathodic sweep, supporting the assignment of the 532 $cm^{-1}$ band to interfacial water rather than carbonate residues.

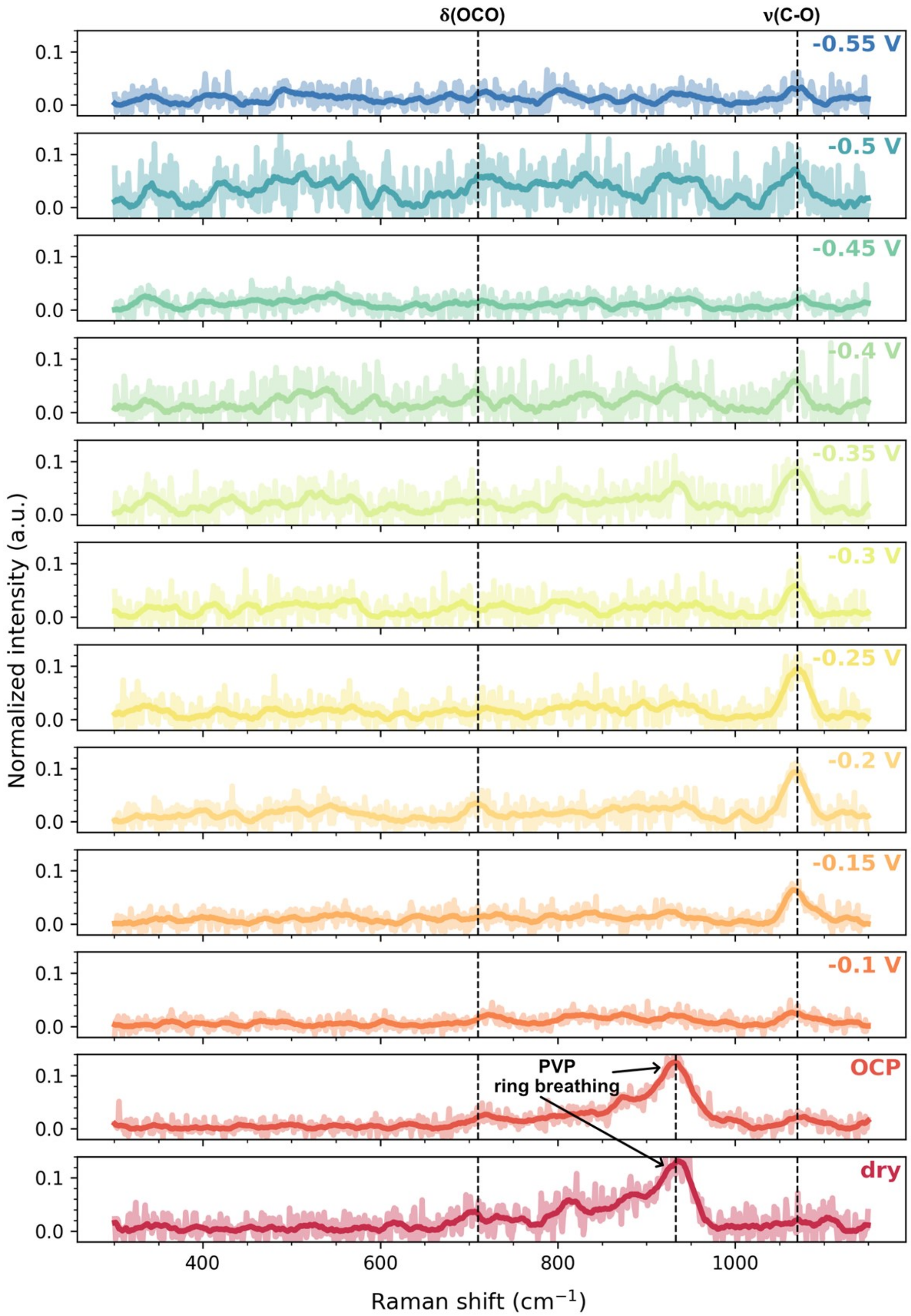


**Figure S34.** Potential-dependent Raman spectra of Ag in 1 M $Na_2CO_3$ from -0.1 V to -0.55 V vs. RHE. No features are observed in the 400–700 cm$^{-1}$ region, while the characteristic carbonate ν(C–O) stretch appears at 1070 cm$^{-1}$ without the δ(O–C–O) bending mode (~700 cm$^{-1}$). The Ag/AgCl/3 M KCl potentials applied here are the same as other Raman measurements. However, since 1 M $Na_2CO_3$ is slightly less alkaline (pH 12.2) than pH 13, the RHE conversion differs by about 0.05 V.

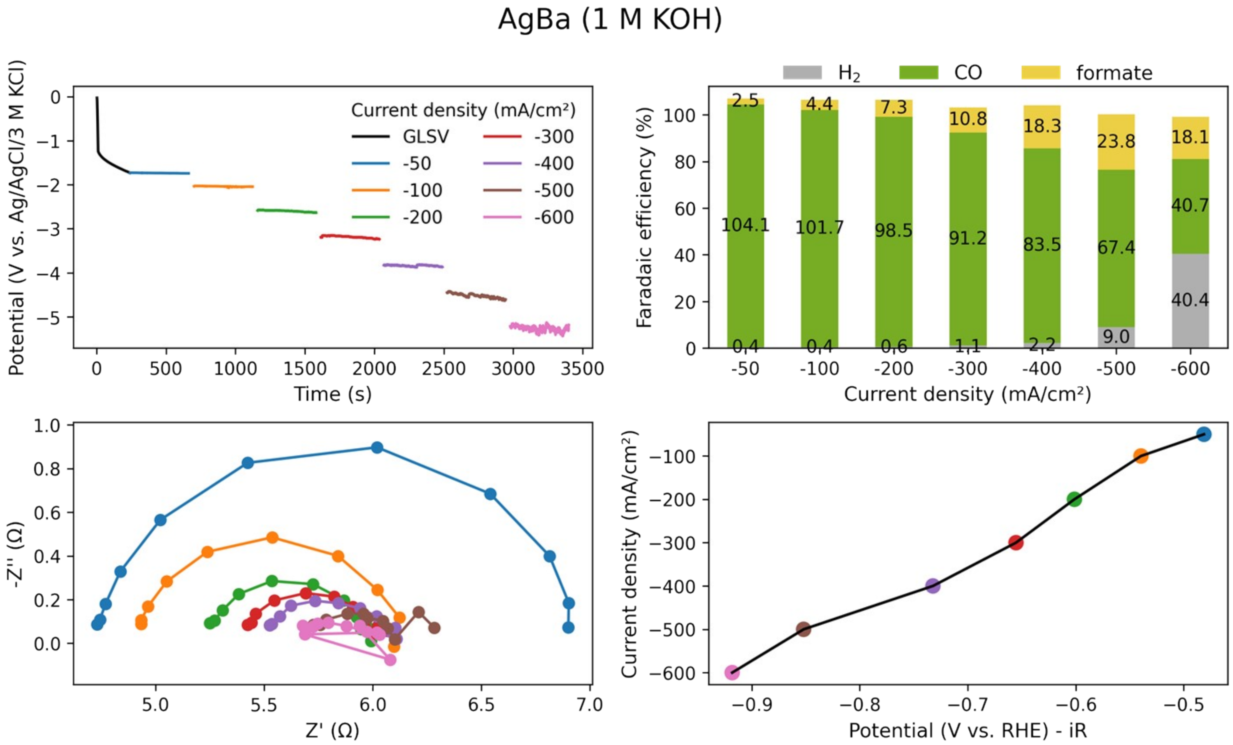


**Figure S35.** Electrochemical performance of AgBa for $CO_2RR$ in 1 M KOH (-50 to -600 mA $cm^{-2}$), which corresponds to approximately -0.5 V to -0.9 V vs. RHE after iR correction.

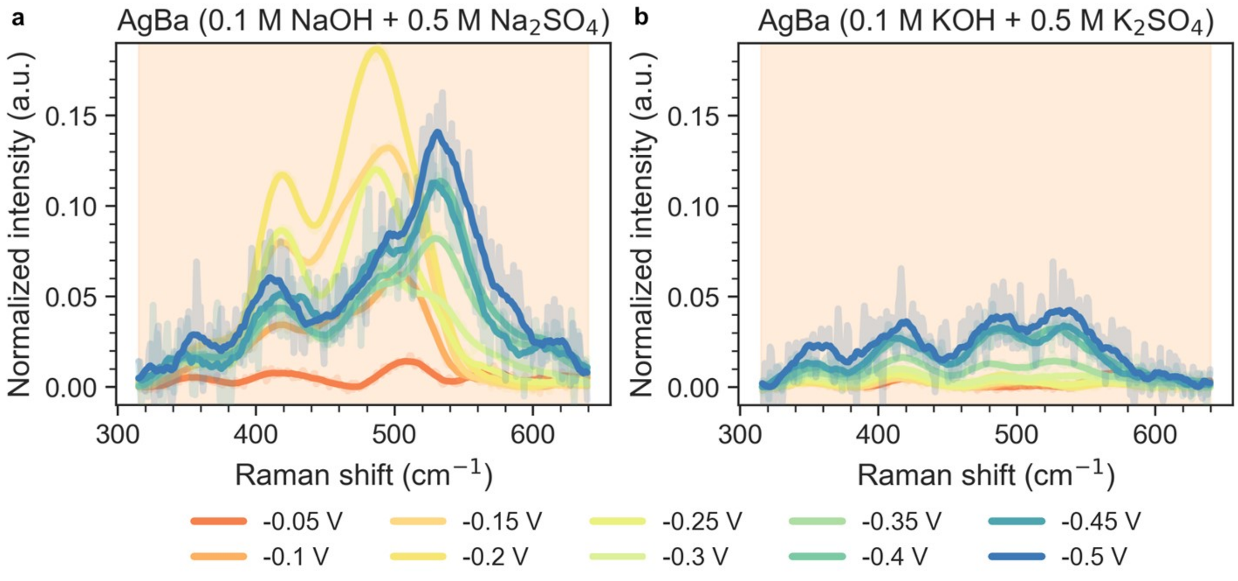


**Figure S36.** Side-by-side comparison of potential-dependent Raman spectra of AgBa in $Na^+$ and $K^+$ electrolytes in the low-wavenumber region (300–640 $cm^{-1}$), plotted on the same scale. (a) corresponds to 0.1 M NaOH + 0.5 M $Na_2SO_4$ (same as Figure 4) and (b) corresponds to 0.1 M KOH + 0.5 M $K_2SO_4$ (same as Figure S37). The signals are significantly suppressed in the $K^+$ electrolyte than $Na^+$. It is however

noted that direct comparison of Raman intensities across different samples should be treated with caution, as the signal intensity is highly sample and position sensitive.

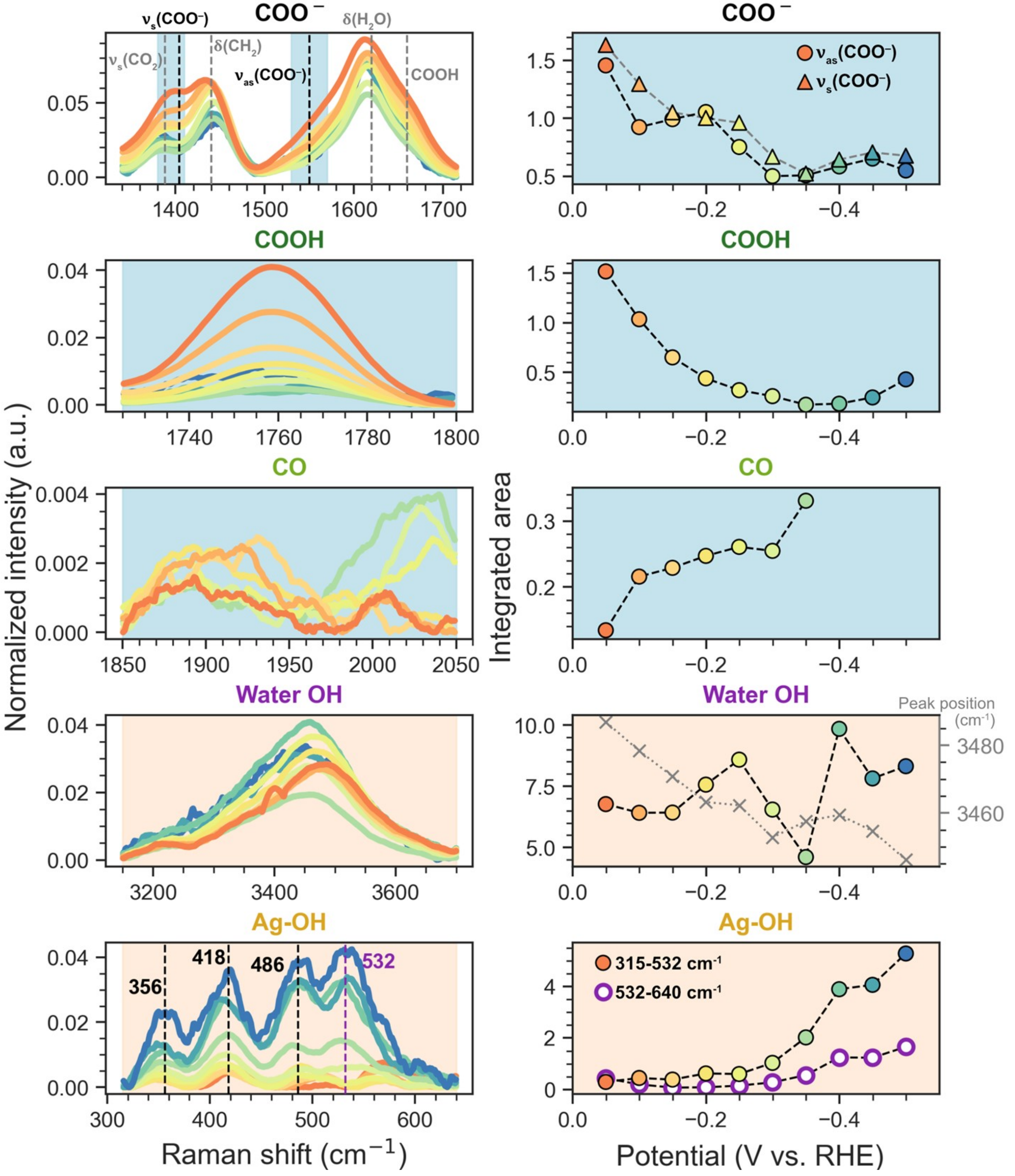


**Figure S37.** Normalized Raman intensities of AgBa measured in 0.1 M KOH + 0.5 M $K_2SO_4$ from −0.05 to −0.5 V vs. RHE for $^*COO^-$, *COOH, *CO, water OH, and Ag–OH, with their corresponding integrated areas. The shaded backgrounds indicate the range for integration. For clarity, only smoothed signals are plotted here. Comparable figure for AgBa in 0.1 M NaOH + 0.5 M $Na_2SO_4$ plotted in Figure 4.

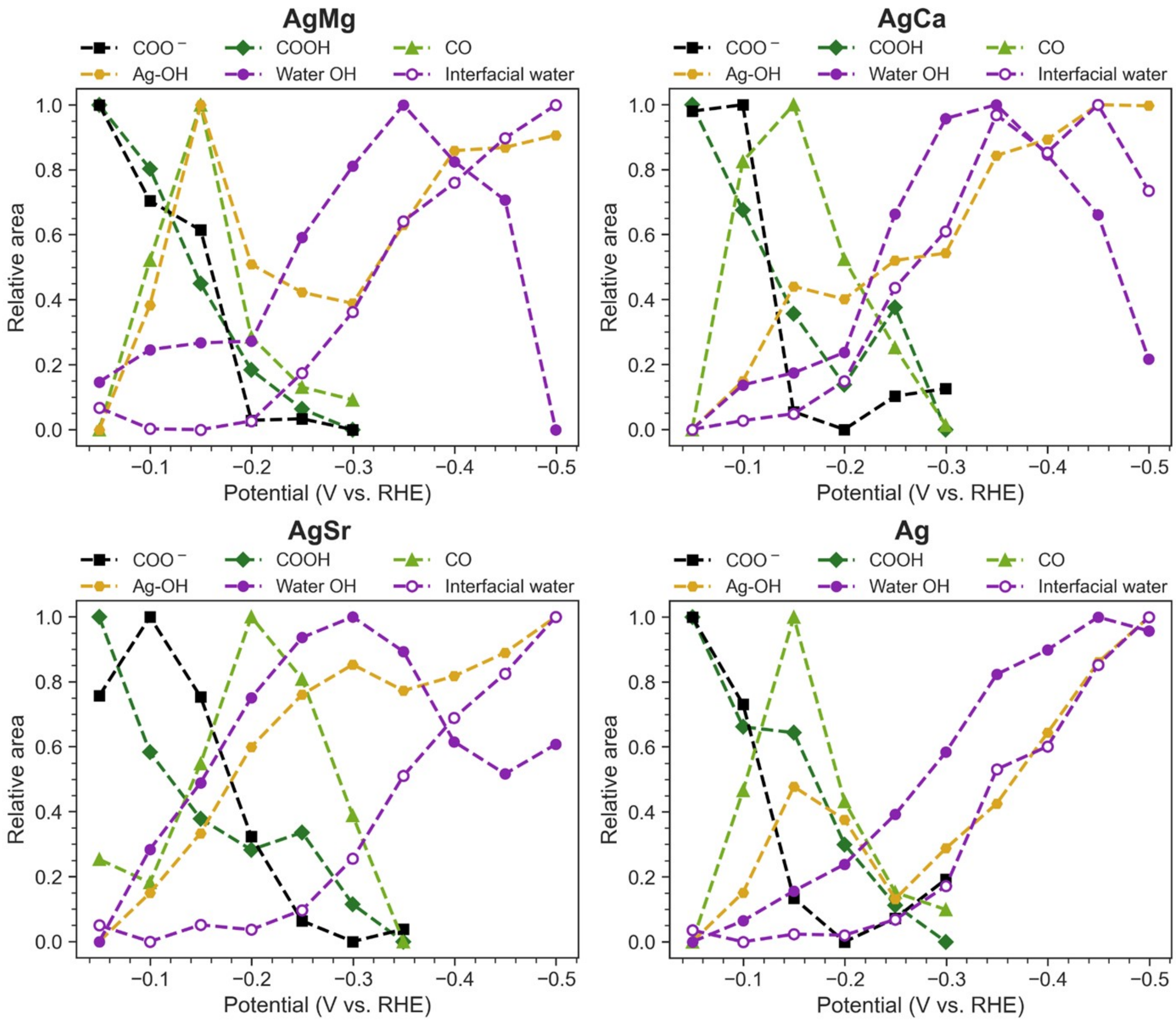


**Figure S38.** Potential-dependent evolution of different species ($COO^-$, COOH, CO, Ag–OH, water OH, and interfacial water) for AgMg, AgCa, AgSr, and Ag. The relative area for each species is obtained by scaling its integrated area to 0–1. Comparable figure for AgBa shown in Figure 6.

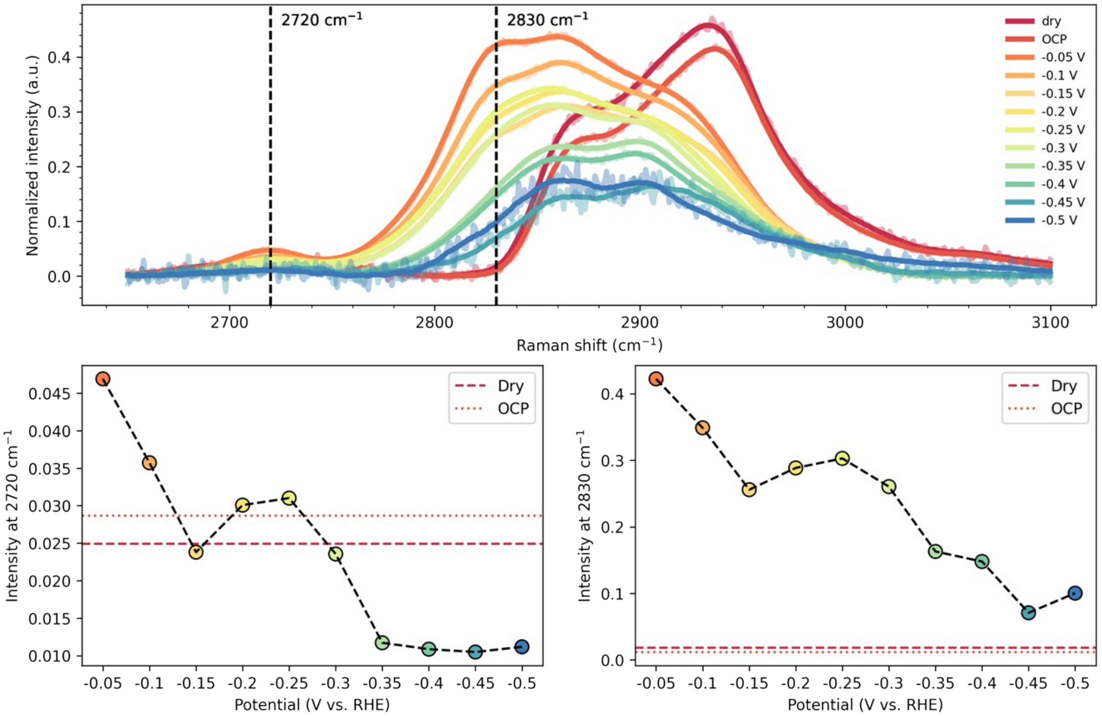


**Figure S39.** C–H stretching vibrations of AgBa at different applied potentials. Strong C–H bands are present even before applying bias due to the presence of PVP, hindering accurate quantification of formate-related C–H signals. Nonetheless, intensities at 2720 and 2830 $cm^{-1}$ generally decrease with increasing cathodic potentials.

## 3. References


1 M. A. A. Mahbub, J. R. C. Junqueira, X. Wang, J. Zhang, S. Dieckhöfer, S. Seisel, D. Das and W. Schuhmann, *Adv. Funct. Mater.*, 2024, **34**, 2307752.

2 D. R. Lide and CRC Press, eds., *CRC Handbook of Chemistry and Physics: A Ready-Reference Book of Chemical and Physical Data*, CRC, Taylor & Francis, Boca Raton, Fla., 87th edn., 2006.

3 E. Ciliberto, G. G. Condorelli, S. La Delfa and E. Viscuso, *Appl. Phys. A*, 2008, **92**, 137–141.

4 P. S. Mdluli, N. M. Sosibo, N. Revaprasadu, P. Karamanis and J. Leszczynski, *J. Mol. Struct.*, 2009, **935**, 32–38.

5 K. I. Hadjiivanov, D. A. Panayotov, M. Y. Mihaylov, E. Z. Ivanova, K. K. Chakarova, S. M. Andonova and N. L. Drenchev, *Chem. Rev.*, 2021, **121**, 1286–1424.

6 W. W. Rudolph and G. Irmer, *J. Solution Chem.*, 2022, **51**, 935–961.

7 M. Gajdo, A. Eichler and J. Hafner, *J. Phys.: Condens. Matter*, 2004, **16**, 1141–1164.

8 F. Abild-Pedersen and M. P. Andersson, *Surf. Sci.*, 2007, **601**, 1747–1753.

9 M.-S. Liao and Q.-E. Zhang, *J. Chem. Soc., Faraday Trans.*, 1998, **94**, 1301–1308.

10 B. W. J. Chen, D. Kirvassilis, Y. Bai and M. Mavrikakis, *J. Phys. Chem. C*, 2019, **123**, 7551–7566.

11 H. Abe, K. Manzel, W. Schulze, M. Moskovits and D. P. DiLella, *J. Chem. Phys.*, 1981, **74**, 792–797.
